# Synergic Catalysis: the Importance of Intermetallic Separation in Co(III)K(I) Catalysts for Ring Opening Copolymerizations

**DOI:** 10.1021/jacs.4c07405

**Published:** 2024-08-09

**Authors:** Francesca Fiorentini, Katharina H. S. Eisenhardt, Arron C. Deacy, Charlotte K. Williams

**Affiliations:** Department of Chemistry, University of Oxford, Oxford OX1 3TA, United Kingdom

## Abstract

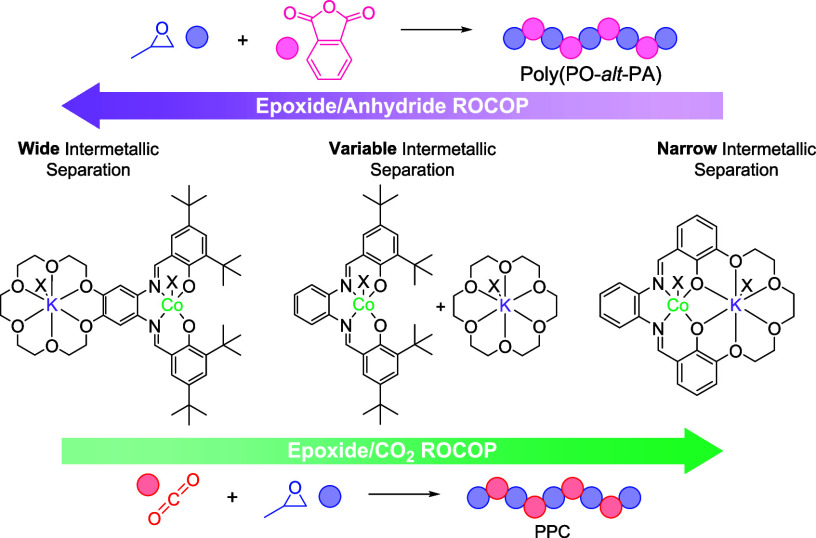

Dinuclear polymerization catalysts can show high activity and control. Understanding how to design for synergy between the metals is important to improving catalytic performances. Three heterodinuclear Co(III)K(I) catalysts, featuring very similar coordination chemistries, are prepared with different intermetallic separations. The catalysts are compared for the ring-opening copolymerization (ROCOP) of propene oxide (PO) with CO_2_ or with phthalic anhydride (PA). The catalyst with a fixed, wide intermetallic separation, L_wide_CoK(OAc)_2_ (Co–K = 8.06 Å), shows very high activity for PO/PA ROCOP, but is inactive for PO/CO_2_ ROCOP. On the other hand, the catalyst with a fixed, narrow intermetallic separation, L_short_CoK(OAc)_2_ (Co–K, 3.59 Å), shows high activity for PO/CO_2_ ROCOP, but is much less active for PO/PA ROCOP. A bicomponent catalyst system, comprising a monometallic complex L_mono_CoOAc used with an equivalent of KOAc[18-crown-6], shows high activity for both PO/CO_2_ and PO/PA ROCOP, provided the catalyst concentration is sufficiently high, but underperforms at low catalyst loadings. It is proposed that the two lead catalysts, L_wide_CoK(OAc)_2_ and L_short_CoK(OAc)_2_, operate by different mechanisms for PO/PA and PO/CO_2_ ROCOP. The new wide separation catalyst, L_wide_CoK(OAc)_2_, shows some of the best performances yet reported for PO/PA ROCOP, and suggests other catalysts featuring larger intermetallic separations should be targeted for epoxide/anhydride copolymerizations.

## Introduction

Heterodinuclear synergy is an attractive means to increase catalytic performance across a range of reactions.^1−5^ In polymerization catalysis, the use of inexpensive and abundant s-block metals as replacements for heavier transition metals is a priority in the design of synergic heterodinuclear catalysts.^3,6−12^ It is especially timely to focus on improving catalysts for polymerizations using bioderived monomers and carbon dioxide, since these sustainable raw materials may help to reduce greenhouse gas emissions associated with polymer manufacturing and reduce energy barriers to efficient recycling.^13−16^ This study seeks further insight into the design of heterodinuclear polymerization catalysts for ring-opening copolymerization (ROCOP) of epoxides with anhydrides or CO_2_ to produce polyesters and polycarbonates, respectively.^17−22^ The monomers can be bioderived, and the oxygenated polymers can be used as plastics, elastomers, coatings, adhesives, sealants, fibers, and surfactants.^9,22−26^ The polymers are amenable to mechanical recycling and chemical recycling to the monomers (for the polycarbonates), and both polymers are hydrolyzable.^15,23,27−31^

Recently, we reported a series of heterodinuclear catalysts, Co(III)M(I/II)(OAc)_2_ (M(I/II) = Group 1 or 2 metal), which were highly active for propene oxide (PO)/carbon dioxide ROCOP.^6,10^ These studies showed that the rate and selectivity of the catalysis correlated with both s-block metal Lewis acidity and the electron density at the Co(III) center.^6,32^ However, the influence of the intermetallic separation, i.e., distance between the Co(III) and M(I), has not yet been explored using the salen/crown ether systems since it is dictated by the ancillary ligand structure. Nonetheless, analysis of the previously reported ROCOP catalysts suggests that it could be a useful parameter to further control rates.^17,33 −35^

Pioneering work from Coates and coworkers established that for Zn(II) β-diimidate (BDI) catalysts, there was a non-linear correlation between the ancillary ligand steric bulk (and by extension, Zn–Zn separation) and catalytic activity, with the best catalysts forming “loosely associated” dimers with intermetallic separations between 4.0 and 4.2 Å (Figures S1 and S2).^34^ Following this landmark report, many researchers targeted homodinuclear catalysts; for example, Rieger and coworkers reported a di-Zn(II) complex, coordinated by a tetra-iminate ligand, which showed a very high activity for cyclohexene oxide (CHO)/CO_2_ ROCOP (TOF = 9130 h^–1^, 1:4000 [catalyst]_0_:[CHO]_0_, 100 °C, 40 bar CO_2_, see Figure S3 for the catalyst structure).^36^ The Zn–Zn separation, as estimated by DFT, was ∼7.77 Å, and the flexible ligand could allow for variation of the distance in the key transition states/intermediates. Indeed, DFT calculations over the entire catalytic cycle showed Zn–Zn separations of 4.50–5.66 Å with ∼5.31 Å calculated in the rate determining transition state.

Prior attempts to control intermetallic separation were not limited to homogeneous catalysts. Heterogeneous Zn(II) dicarboxylate epoxide/CO_2_ catalysts were also investigated experimentally and computationally, and it was proposed that Zn–Zn separations should be 4.3–5.0 Å for optimized performances.^41^

We analyzed data for the “best” multinuclear catalysts reported for epoxide/anhydride and/or epoxide/carbon dioxide ROCOP (Figure 2).^1,7,8,11,34−36,42−47^ To measure the intermetallic separation, data from single crystal X-ray diffraction experiments were used, or for catalysts where XRD was not reported, the distances were estimated from DFT calculations. These catalysts are evaluated for the ROCOP of CHO with phthalic anhydride (PA) or carbon dioxide; CHO is often studied due to its high ring stain and high boiling point, which allow for copolymerizations at higher temperatures and, for carbon dioxide ROCOP, its higher barriers to cyclic carbonate (byproduct) formation particularly when compared with alkylene oxide/CO_2_ ROCOP.^20−22^ Thus, we plotted the activity for high-performance bimetallic catalysts for CHO/PA and CHO/CO_2_ ROCOP against intermetallic separation. The plot reveals that the best catalysts show two distinct regions for the intermetallic separation, either between 3.0 and 4.5 or between 7.0 and 9.0 Å (Figures 2, S3, and S4, Tables S1 and S2).

**Figure 1:**
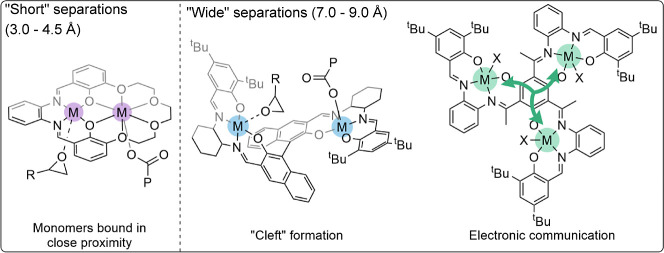
Illustration of leading catalysts by their intermetallic separations and the proposed rationale for metallic synergy.^17,37−40^.

**Figure 2:**
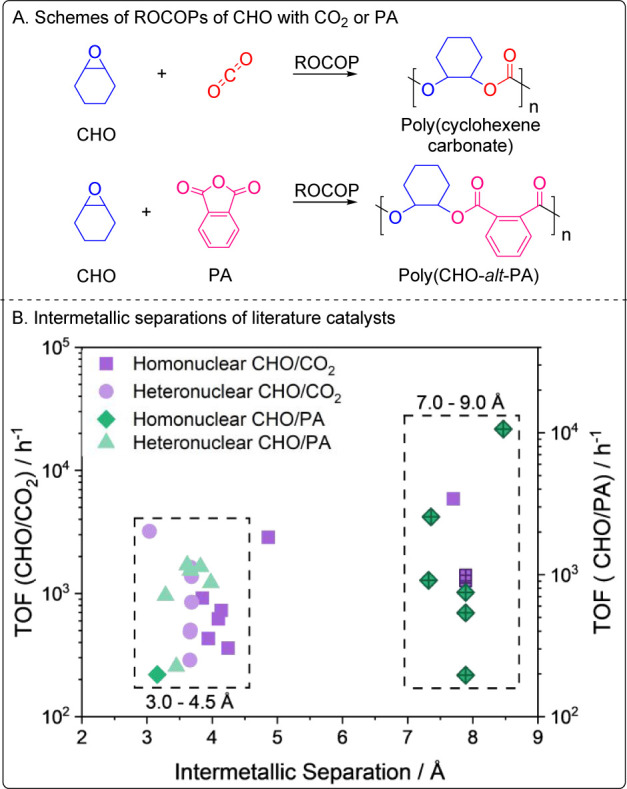
(A) ROCOP of CHO with CO_2_ or PA. (B) Analysis of literature dinuclear catalysts for CHO/CO_2_ or CHO/PA ROCOP, identifying optimal intermetallic separations within those structures. Activity (turn over frequency, TOF) for catalysts for CHO/CO_2_ ROCOP (CO_2_ pressures > 5 bar) (LHS *y*-axis, purple squares and circles) and for CHO/PA ROCOP (RHS *y*-axis, green diamonds and triangles) is plotted against intermetallic separation (from solid-state structures or calculated by DFT). Catalysts requiring cocatalysts (e.g., PPNCl) have an added cross.^1,7,8,11,34 −36,42−47^.

Rationalizing the two “separations” is challenging. Most authors rationalize high-performance “narrow” separation catalysis by a chain shuttling mechanism, where close intermetallic separations reduce barriers in the rate limiting step by cooperation between the “nucleophilic” polymer chain-ends, which attacks a monomer coordinated at the second metal (Figures 1 (LHS), S5, and S6).^17,37^ For catalysts with wider intermetallic separations, synergy explanations depend on the ligand. For conjugated planar ligands, it is claimed there may be “electronic communication” between metals, which enhances rates (Figure 1, RHS), while for nonplanar ligands, the rationale tends to invoke ligand flexibility allowing access to a catalytic “cleft” where the metals point toward each other and undergo cooperative catalysis in an analogous way to the mechanisms proposed for narrow separation catalysts (Figure 1, middle).^38 −40^ One challenge with applying these concepts to the new catalyst design is that explanations are specific to particular catalysts; there is a need for systematic investigation of the influence of metallic separation, and in particular, it would be desirable for such studies to fix the metals and coordination chemistry (ligand donors) while varying only the metal separations. Indeed, a recent evaluation of other carbon dioxide activation catalyzes identifies intermetallic distance as a key design parameter.^48^

**Figure 3:**
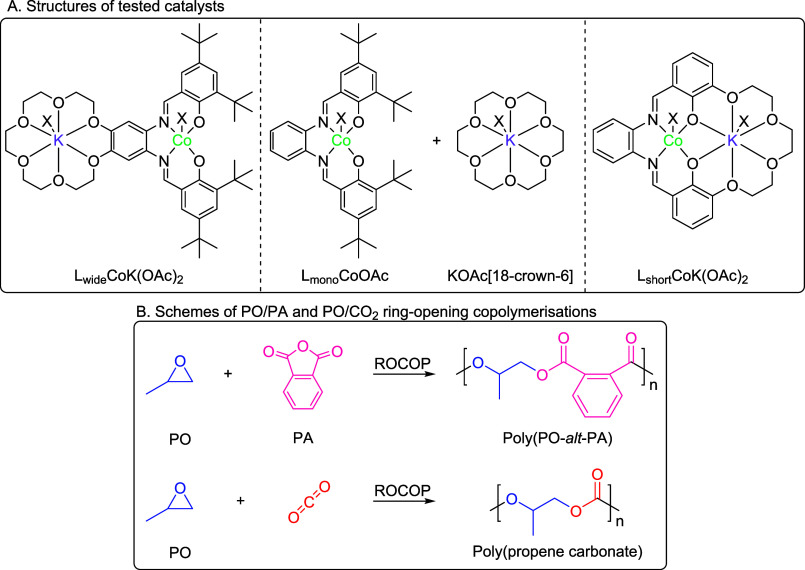
(A) Structures of the catalysts tested for PO/PA and PO/CO_2_ ROCOP. X = OAc. (B) Schemes for the tested ring-opening polymerizations of propene oxide with phthalic anhydride or CO_2_.

In this work, we compare two Co(III)K(I) heterodinuclear catalysts with fixed intermetallic separations in each of the two key intermetallic regions identified from the literature analysis, i.e., 7.0–9.0 Å (L_wide_CoK(OAc)_2_) or 3.0–4.5 Å (L_short_CoK(OAc)_2_ (Figure 3A). These catalysts should operate effectively without any cocatalyst or additive since the goal is to understand intermetallic separation influences. To investigate a catalyst with the potential for variable intermetallic separations, a bicomponent catalyst was targeted where a Co(III) complex (L_mono_CoOAc) is applied with an equivalent of a potassium carboxylate salt (KOAc[18-crown-6] (Figure 3A). These catalysts are prioritized for ROCOP using PO with PA or CO_2_ (Figure 3B). These monomers are already produced and used on a very large scale globally; have strong potential to be bioderived; or, in the case of CO_2_, is an existing waste.^49,50^ The monomer combinations are also very demanding and hence require better catalysts, with typical activities and selectivity values being lower than when using CHO (lower temperatures are necessary and CO_2_/PO ROCOP may form a propene carbonate byproduct).^17^ The investigation objective is to understand the influences of intermetallic separations in both polyester (PO/PA) and polycarbonate (PO/CO_2_) polymerization catalysts.

## Results and Discussion

The pro-ligand (H_2_L_wide_), targeted to produce a wide intermetallic separation catalyst, was synthesized from benzo-18-crown-6, with an overall yield of 73% by using a modified literature procedure (Figure S7).^51^ The novel catalyst, L_wide_CoK(OAc)_2_, was synthesized following a two-step, one-pot procedure (Figure S8). First, a suspension of the ligand, in methanol, was combined with KOAc, and the mixture was refluxed for 1 h under a nitrogen atmosphere. Next, an equivalent of Co(OAc)_2_ was added, at room temperature, and the mixture was stirred for 16 h. The L_wide_Co(II)KOAc complex was oxidized to the L_wide_Co(III)K(OAc)_2_ catalyst by the addition of acetic acid and stirring the solution in air for 4 h. The target catalyst, L_wide_CoK(OAc)_2_, was isolated as a very dark, red-purple powder in 62% yield. The catalyst was characterized by ^1^H, ^13^C{^1^H}, COSY, HSQC, HMBC NMR, IR, and UV–vis spectroscopy, as well as by MALDI-ToF mass spectrometry (Figures S9–S16). Purity was determined by elemental analysis.

The successful catalyst synthesis was confirmed by ^1^H NMR spectroscopy by the disappearance of the pro-ligand phenol proton resonance (13.51 ppm), and by IR spectroscopy where the pro-ligand phenol O–H stretch (3590 cm^–1^) disappeared (Figures S18 and S19). The desired complex formation was indicated by UV–vis spectroscopy, where the peak absorption, λ_max_, shifted from 270 nm (pro-ligand) to 280 nm (catalyst). The absorption is proposed as a ligand, π→π* transition. There is also a shift in the transition at 357 nm in the proligand to 402 nm in the complex. This is tentatively assigned as an n→π* transition, with the red-shift corresponding to a weaker C=N bond after coordination to Co(III) (Figure S20). The catalyst also shows a new absorption at 512 nm, which is assigned as a Co(III) d→d transition. MALDI-ToF mass spectrometry shows a peak at 870.07 *m*/*z*, corresponding to [L_wide_Co(II)K(I)]^+^ (Figure S16). No peaks corresponding to the ligand, mononuclear, or homodinuclear complexes could be observed. The isotopic distribution of the peak at 870.06 *m*/*z* is in agreement with the calculated distribution pattern (Figure S17).

While single crystals of L_wide_CoK(OAc)_2_ could not be grown, a structurally equivalent L_wide_NiKOAc complex was synthesized.^52^ The complex was characterized by single crystal X-ray diffractometry and showed a Ni(II)–K(I) separation of 8.06 Å (Figures S22 and S23). It is proposed that the L_wide_CoK(OAc)_2_ catalyst should show a very similar intermetallic separation as the ionic radii of Co(III) and Ni(II) are very similar (0.55 and 0.49 Å, respectively).^53^

The Co(III) complex for the bicomponent catalyst, L_mono_Co(OAc), was synthesized according to a literature procedure with a yield of 92%.^54^ The narrow separation catalyst, L_short_CoK(OAc)_2_, was synthesized from the dialdehyde pro-ligand with a yield of 64% according to a literature procedure.^32^ Spectroscopic characterization data for both L_mono_CoKOAc and L_short_CoK(OAc)_2_ matched those previously reported, and complex purity was determined by elemental analysis.

The three catalyst systems were tested for PO/PA ROCOP, using conditions of 1:20:400:1000 of [catalyst]_0_:[BDM]_0_:[PA]_0_:[PO]_0_, in neat epoxide, at 60 °C. 1,4-Benzene dimethanol (BDM) is used as a chain transfer agent. It is widely used to control the molar mass of the resultant polymers; catalysts must be stable to the excess alcohol and able to rapidly and reversibly react with it.^55^ Under these conditions, L_wide_CoK(OAc)_2_ displayed the highest activity with an exceptional TOF of 1623 h^–1^ (Table 1, entry 1). It was also fully selective for polyester linkages; no ether linkages are observed by ^1^H NMR spectroscopy. The polyester shows a molar mass value close to that predicted theoretically (2100 vs 2800 g mol^–1^). It also has a monomodal, narrow dispersity molar mass distribution, as assessed by GPC (Figure 25). Both features indicate rapid and reversible chain transfer, and are indicative of catalysis with a high degree of polymerization control. The control is further supported by the linear fit to plots of polyester *M*_n_ vs conversion, where *M*_n_ values are always close to theoretical values (Figure S26). Higher molar mass polyesters were synthesized by reducing (or removing) the quantity of BDM (chain-transfer agent) used in the catalysis (Figure S25).

**Table 1:** Data for the ROCOP of Propene Oxide (PO) and Phthalic Anhydride (PA) for the Three Catalysts^a^

entry	catalyst	time/h	TON^b^	TOF^c^/h^–1^	*M*_n_ [*Đ*]^d^/g mol^–1^
1	L_wide_CoK(OAc)_2_	0.2	281	1686	2100 [1.11]
2^e^	L_wide_CoK(OAc)_2_	0.8	1184	1480	12900 [1.07]
3	L_mono_CoOAc	6	316	53	2800 [1.10]
4	KOAc[18-crown-6]	8	289	36	2100 [1.22]
5	L_mono_CoOAc + KOAc[18-crown-6]	0.4	275	1238	2700 [1.08]
6^e^	L_mono_CoOAc + KOAc[18-crown-6]	22.5	837	37	6500 [1.18]
7	L_short_CoK(OAc)_2_	1.2	270	231	2100 [1.11]

a Reaction conditions: PO/PA 1:20:400:1000 [cat]_0_:[BDM]_0_:[PA]_0_:[PO]_0_, neat, 60 °C.

b Turnover number (TON) determined by the number of moles of converted PO divided by moles of catalyst, where conversions are determined by ^1^H NMR spectroscopy (Figure S24).

c Turnover frequency (TOF) calculated from TON divided by the time between 20% and 80% conversion. Determined by the comparison of the integrals for any ether resonances (3.4–3.6 ppm) with the resonances for polyester (7.3 ppm) via ^1^H NMR spectroscopy (Figure S24).

d Determined by GPC, in THF, using narrow dispersity polystyrene standards to calibrate.

e Reaction conditions: PO/PA 1:20:1600:4000 [cat]_0_:[BDM]_0_:[PA]_0_:[PO]_0_, neat, 60 °C.

The two components of the bicomponent catalyst were each tested separately, i.e., the Co(III) complex, L_mono_CoOAc, and the potassium salt, KOAc[18-crown-6]. Both species show low catalytic activities of 53 h^–1^ and 36 h^–1^, respectively, when tested separately, highlighting the benefits and synergy observed for the mixed metal catalysts (Table 1, entries 3 and 4). The bicomponent catalyst system, i.e., 1:1 mixture of L_mono_CoOAc:KOAc[18-crown-6], was an effective catalyst showing a turn over frequency (TOF) of 1238 h^–1^ (measured between 25–80% PA conversion) (Table 1, entry 5). The bicomponent catalyst does show a significant initiation period, and if only the single time point activity is considered, then the TOF is reduced to 749 h^–1^ (Figure S27). This initiation period may be due to the requirement of the bicomponent system to form the dinuclear species in solution. While the bicomponent catalytic activity is lower than the activity of L_wide_CoK(OAc)_2_, it is still very high for PO/PA ROCOP. One disadvantage of bicomponent catalysts is that they cannot be applied under low catalyst loadings. Indeed, when this bicomponent system is applied under more forcing conditions, 1:20:1600:4000 [catalyst]_0_:[BDM]_0_:[PA]_0_:[PO]_0_, its activity drops by 97% to 37 h^–1^ (Table 1, entry 6). On the other hand, the single-component dinuclear catalyst, L_wide_CoK(OAc)_2_, largely maintains its activity under these same forcing conditions, with only a 13% reduction in rate to 1480 h^–1^ (Table 1, entry 2). All the catalysts were fully selective for polyester linkages.

The wide separation heterodinuclear catalyst, L_wide_CoK(OAc)_2_, has 40× greater activity than the bicomponent analogue at this concentration. These findings emphasize the importance of controlling metallic separation through the ancillary ligand. The catalysts were also tested under demanding conditions, with a high loading of anhydride relative to the catalyst, demonstrating the robustness of these systems. The narrow-separation, heterodinuclear catalyst, L_short_CoK(OAc)_2_, did not have a very high activity, with a TOF of 231 h^–1^, a value that is markedly lower than the activities of L_wide_CoK(OAc)_2_ or the bicomponent catalyst system, under the same conditions (Table 1, entry 7).^32^

The three catalysts L_wide_CoK(OAc)_2_, L_mono_CoOAc + KOAc[18-crown-6], and L_short_CoK(OAc)_2_ were also tested for PO/CO_2_ ROCOP (Table 2). Polymerizations were conducted using 1:20:4000 [catalyst]_0_:[*trans*-1,2-cyclohexane diol (CHD)]_0_:[PO]_0_ at 50 °C and 20 bar of CO_2_ pressure. The diol (CHD) is a chain-transfer agent and was used to allow comparison to the literature. While the wide separation catalyst, L_wide_CoK(OAc)_2_, was the most active catalyst for PO/PA ROCOP, it is not very effective for PO/CO_2_ ROCOP. Over 27.5 h, it produced no polycarbonate and only small quantities of propene carbonate (cyclic byproduct) and polyether (Table 2, entry 1). The other two catalysts were active and more selective for polycarbonate formation. The bicomponent catalyst system shows a TOF of 16 h^–1^, even under the relatively high catalyst dilution of the test conditions (Table 2, entry 2). However, it was only moderately selective, with a polycarbonate selectivity of just 53%. It also formed 36% propene carbonate, and the polymers show ∼11% ether linkages. The formation of ether linkages is, perhaps, predictable, as L_mono_CoOAc is a known catalyst for PO ring opening polymerization.^54^ Increasing the concentration of the catalyst to 1:20:1000 [catalyst]_0_:[CHD]_0_:[PO]_0_ resulted in both higher activity (TOF = 231 h^–1^) and polycarbonate selectivity (82%, Table 2, entry 3). Only 1% cyclic carbonate was produced; however, the polymer shows a mixed composition with an even higher ether linkage proportion of 17%. When using higher catalyst concentrations, the bicomponent catalyst system shows comparable performance to other PO/CO_2_ catalysts reported in the literature.,^10,56,57^

**Table 2:** Data for the ROCOP of Propene Oxide (PO) and CO_2_ for the Three Catalysts^a^

entry	catalyst	time/h	TON^b^	TOF_PPC_^c^/h^–1^	PPC^d^/%	PC^e^/%	PPO^f^/%	*M*_n_ [*Đ*]^g^/g mol^–1^
1	L_wide_CoK(OAc)_2_	27.5	207	0	0	97	3	-
2	L_mono_CoOAc + KOAc[18-crown-6]	25.0	405	16	53	36	11	<1000
3^h^	L_mono_CoOAc + KOAc[18-crown-6]	0.4	112	231	82	1	17	<1000
4^32^	L_short_CoK(OAc)_2_	2.5	973	389	>99	0	0	4000 [1.06]

a Reaction conditions: 1:20:4000 [catalyst]_0_:[CHD]_0_:[PO]_0_, neat, 50 °C, 20 bar CO_2_.

b Turnover number (TON) = moles of PO consumed/moles of catalyst, determined by ^1^H NMR spectroscopy (Figure S28).

c Turnover frequency for PPC production (TOF_PPC_) = TON for PPC production/time.

d Selectivity for poly(propene carbonate) (PPC) determined by measuring the integrals for PPC compared to all other products in the reaction by ^1^H NMR spectroscopy (Figure S28).

e Selectivity for propene carbonate (cyclic by-product; PC) determined by measuring the integrals for PC compared to all other products in the reaction by ^1^H NMR spectroscopy (Figure S28).

f Selectivity for polypropene oxide (polyether; PPO) determined by measuring the integrals for PPO compared to all other products in the reaction by ^1^H NMR spectroscopy (Figure S28).

g Determined by GPC, in THF, using narrow dispersity polystyrene standards.

h Reaction conditions: 1:20:1000 [catalyst]_0_:[CHD]_0_:[PO]_0_, neat, 50 °C, 20 bar CO_2_.

L_short_CoK(OAc)_2_ is by far the most active and selective of the series of catalysts. It shows a high TOF of 389 h^–1^ and quantitative selectivity for polycarbonate formation, with no detectable propene carbonate or ether linkages as determined by ^1^H NMR spectroscopy (Table 2, entry 4). This catalyst is among the leading catalysts in the field of PO/CO_2_ ROCOP. It has a superior performance to a Co(III) salen system when applied without cocatalyst (TOF = 75 h^–1^, PPC > 99%, 1:500 [catalyst]_0_:[PO]_0_, neat, 22 °C, 55 bar CO_2_) and a Co(III) salen with tethered piperidinium cocatalysts, albeit at a higher temperature (TOF = 177 h^–1^, PPC = 99%, 1:2000 [catalyst]_0_:[PO]_0_, 1:1 PO/DME, 14 bar CO_2_, 25 °C).^56,58^ The tolerance of L_short_CoK(OAc)_2_ to CTA is also noteworthy; when the Co(III) salen/dipiperidinium catalyst is used with 20 eq. of methanol, the activity drops to 95 h^–1^.^58^ The catalyst has a lower activity than a Co(III) salen with four tethered quaternary ammonium cocatalysts, reported by Lu and co-workers (TOF = 18900 h^–1^, PPC > 99%, 1:50:100 000 [catalyst]_0_:[adipic acid]_0_:[PO]_0_, neat, 75 °C, 25 bar CO_2_; for structures, see Figure S28).^59,60^ While it is undeniably an excellent catalyst, its synthesis is rather complex (requiring 8 synthetic steps), and it is applied at a higher temperature than L_short_CoK(OAc)_2_.

### Catalyst Tolerance to Chain Transfer Agents

**Figure 4:**
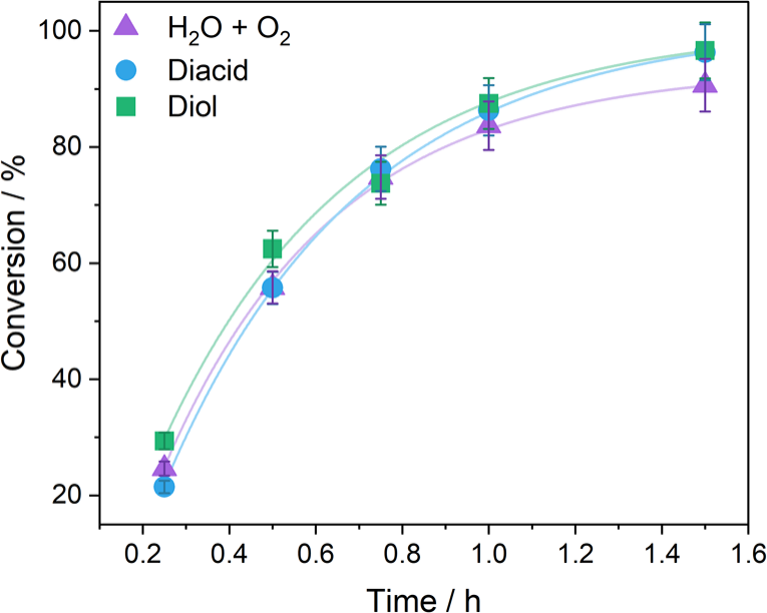
Plot of conversion vs time for polymerizations conducted with 100 equiv of water (aliquoted in air; H_2_O + O_2_), phthalic acid (diacid), and trans-1,2-cyclohexane diol (diol).

In this field of catalysis, chain transfer agents are frequently added to control the polymer molar mass and deliver high end-group selectivity. As such, catalysts must tolerate a large excess of alcohols, and as a further benefit, air-stable catalysts are desirable. To test the chemical stability of L_wide_CoK(OAc)_2_, polymerizations were conducted with an excess (100 eq. relative to catalyst; 0.24 mM) of 1,2-benzoic acid (*ortho*-phthalic acid; diacid), 1,2- cyclohexane diol (CHD; diol), or water (Figure 4). When water was used, aliquots were removed by opening to air to investigate whether air (oxygen) influenced the reaction. In each case, the additives did not compromise the overall conversion to polymer, which remained very high (83–96%) over the 2 h of each experiment. These data illustrate the exceptional chemical stability of the catalyst.

### Polymerization Catalysis: Electronics vs Intermetallic Separations

Recently, we reported upon a series of narrow separation catalysts, including L_short_CoK(OAc)_2_, where the ancillary ligand was modified to include electron-withdrawing and electron donating groups. The investigation revealed that the most electron-rich Co(III) complexes (as determined by Co(III/II) redox potentials, measured by cyclic voltammetry) were also the most active and selective for PO/CO_2_ ROCOP. To understand whether the catalytic performance differences in PO/CO_2_ ROCOP between these three catalysts might correlate with electronic factors, the Co(III/II) redox potentials were measured (Figures S29 and S30).^32^

The Co(III/II) redox potentials of the wide and bicomponent catalysts, L_wide_CoK(OAc)_2_ and L_mono_CoOAc, are nearly identical, with values of −0.23 and −0.22 V (vs ferrocene/ferrocenium), respectively. On the other hand, the equivalent Co(III/II) redox potential for the narrow separation catalyst, L_short_CoK(OAc)_2_, was significantly lower at −0.41 V (vs ferrocene/ferrocenium), indicating it has a more electron-rich Co(III) center.^32^ While this might initially seem to explain the differences in activity observed for PO/CO_2_ ROCOP, it does not explain the difference in activity observed between L_wide_CoK(OAc)_2_ and the bicomponent system, as they have essentially identical Co(III/II) redox potentials. Further, a narrow separation catalyst analogous to L_short_CoK(OAc)_2_ but with a chlorinated phenylene amine linker was reported in the previously discussed article (Figure S31).^32^ This catalyst has a very similar Co(III/II) redox potential to L_wide_CoK(OAc)_2_ and L_mono_CoOAc of −0.24 V (vs ferrocene/ferrocenium) but has an activity of 62 h^–1^ and a selectivity for polycarbonate of 75% for PO/CO_2_ ROCOP (1:20:4000 [catalyst]_0_:[CHD]_0_:[PO]_0_, neat, 50 °C, 20 bar CO_2_; Table S3). This is significantly faster and more selective than both L_wide_CoK(OAc)_2_ and the bicomponent system under the same conditions.

As such, the activity differences between L_wide_CoK(OAc)_2_, the bicomponent catalyst, and L_short_CoK(OAc)_2_ seem unlikely to be related to only different Co(III) electronics factors and more likely related to the difference in intermetallic separation.

**Figure 5:**
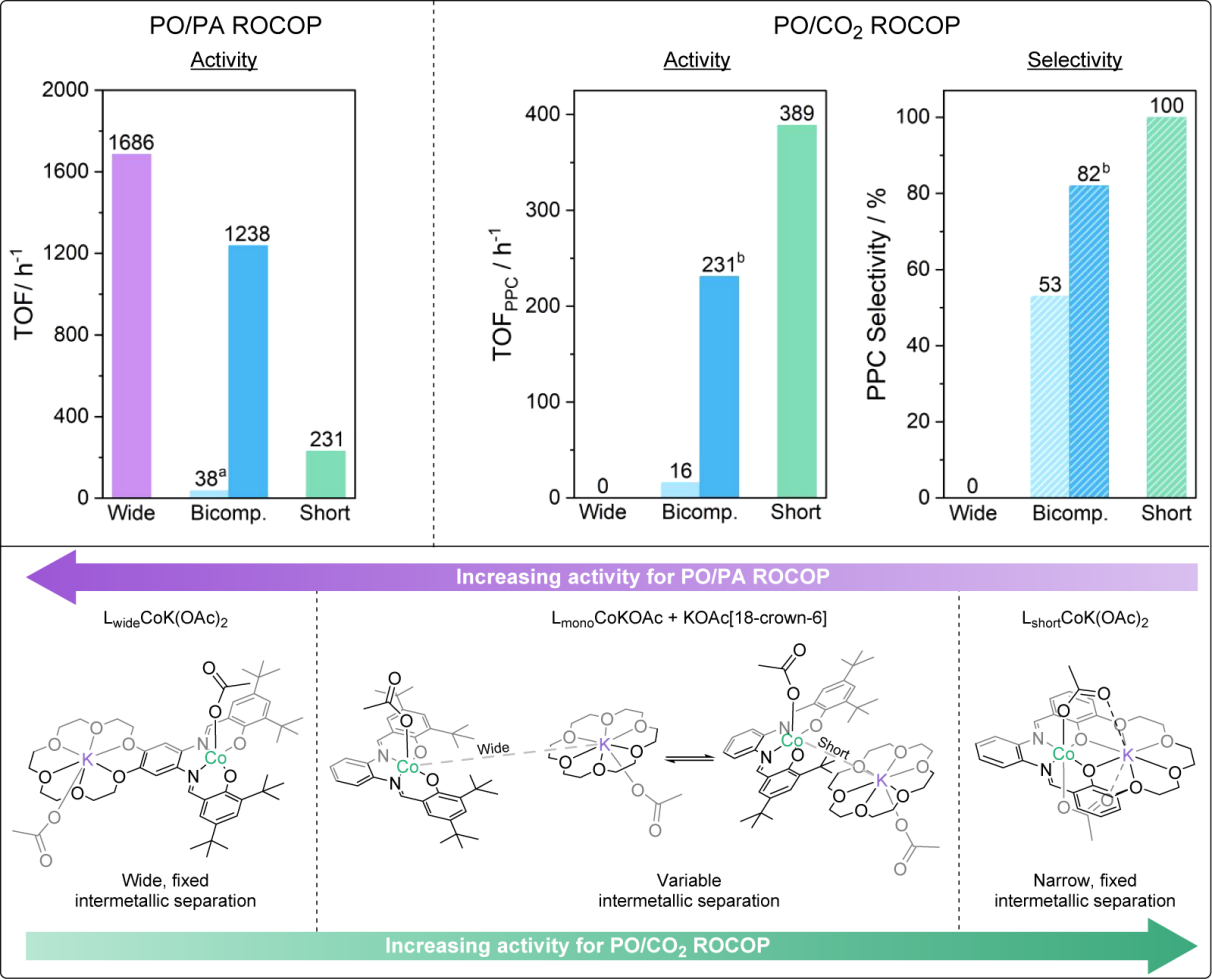
Top: comparison of the three catalysts in terms of activities (and selectivities, where relevant) for PO/PA ROCOP (LHS) and PO/CO_2_ ROCOP (RHS). Bottom: proposed structures of the three dinuclear catalyst systems during polymerization change with intermetallic separation. Reaction conditions: PO/PA ROCOP: 1:20:400:1000 (^a^1:20:400:4000) [catalyst]_0_:[BDM]_0_:[PA]_0_:[PO]_0_, neat, and 60 °C; PO/CO_2_ ROCOP: 1:20:4000 (^b^1:20:1000) [catalyst]_0_:[CHD]_0_:[PO]_0_, 50 °C, and 20 bar CO_2_.

To rationalize the experimental data for the two polymerizations, an alternative hypothesis is that L_wide_CoK(OAc)_2_ and L_short_CoK(OAc)_2_ show well-defined intermetallic separations of 8.06 and 3.59 Å, respectively. In contrast, the bicomponent catalyst system forms its dinuclear structure under the conditions of the catalysis and may be able to do so with variable intermetallic separations. For PO/PA ROCOP, the large intermetallic separation appears to result in L_wide_CoK(OAc)_2_ showing the highest activity, whereas the narrow intermetallic separation of L_short_CoK(OAc)_2_ is the least active. The bicomponent catalyst system is proposed to associate in solution to form a species with intermetallic separations similar to catalyst L_wide_CoK(OAc)_2_, allowing it to show high activity provided the catalyst concentration is also high, i.e., provided both the Co(III) and K(I) can associate in solution (Figure 5). The opposite trend is seen for PO/CO_2_ ROCOP catalysts where the narrow intermetallic separation of L_short_CoK(OAc)_2_ allows it to achieve the highest rates and selectivity. The large intermetallic separation of L_wide_CoK(OAc)_2_ results in complete catalyst deactivation and no formation of any polycarbonate. It is proposed that the bicomponent catalyst system is able to form a dinuclear species with a narrow intermetallic separation, which results in moderate PO/CO_2_ ROCOP performance, particularly at high catalyst concentrations when the probability of the two metals associating is highest (Figure 5).

**Figure 6:**
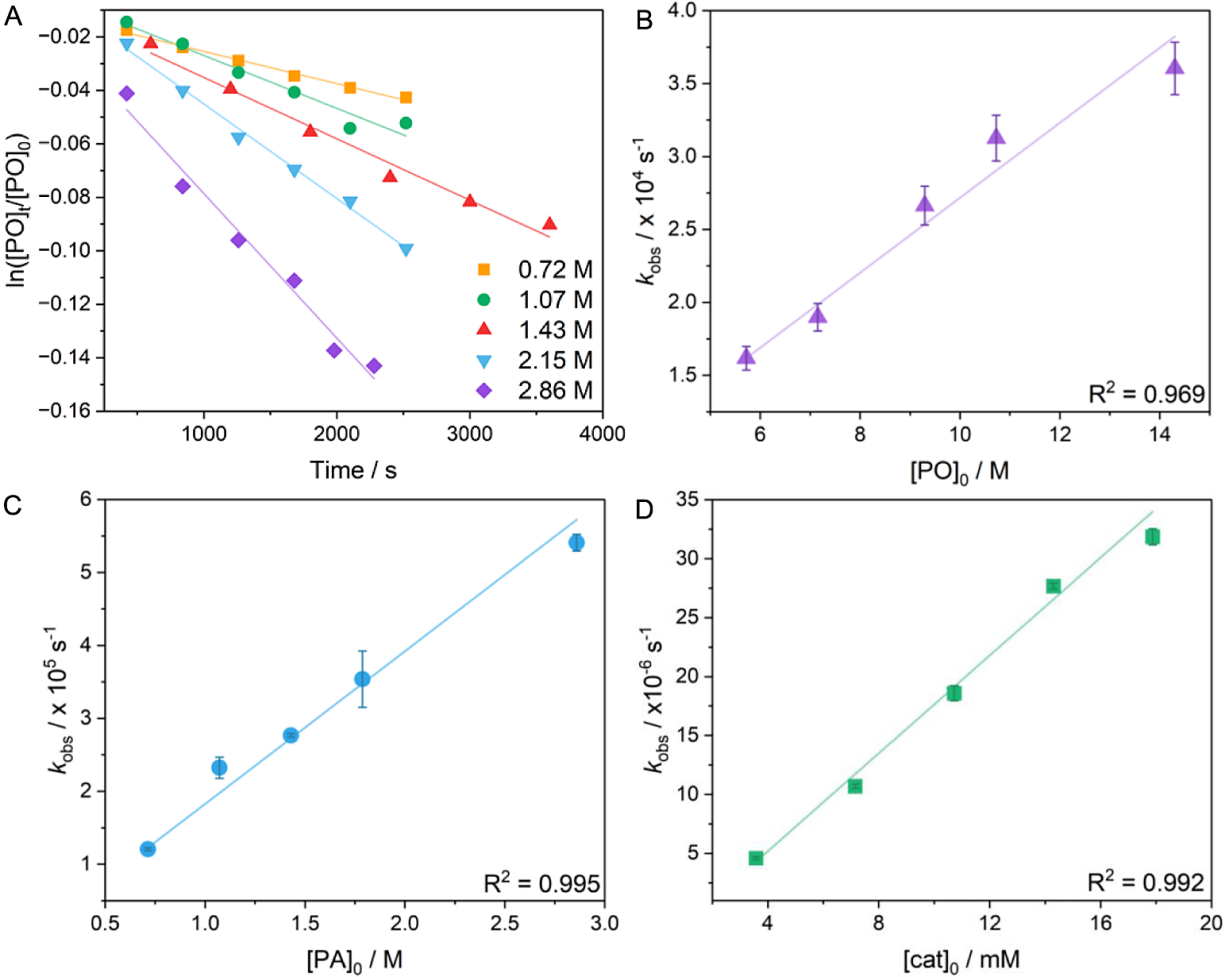
Polymerization kinetics using L_wide_CoK(OAc)_2_ for PO/PA ROCOP. (A) Linear plots of ln([PO]_t_/[PO]_0_) against time with varying [PA]_0._ (B) Plot of *k*_obs_ against [PO]_0_ for the determination of reaction order with respect to PO concentration. (C) Plot of *k*_obs_ against [PA]_0_ for the determination of reaction order with respect to PA concentration. (D) Plot of *k*_obs_ against [catalyst]_0_ for the determination of reaction order with respect to catalyst concentration.

To investigate the possible mechanism for PO/PA ROCOP, the rate law for the most active catalyst, L_wide_CoK(OAc)_2_, was determined. Isolation method integrated rate treatments were used to determine the relative dependence of rates on the concentrations of PO and PA, respectively. In both cases, the polymerizations were carried out using different starting concentrations of the monomer under investigation (0.7–2.9 M for [PA]_0_ or 5.8–14.3 M for [PO]_0_) with [catalyst]_0_ fixed at 14.3 mM and the other monomer starting concentrations held constant; all reactions were isothermal at 60 °C. The polymerizations were monitored by the regular removal of aliquots, which were analyzed, to determine the conversion of PA, using ^1^H NMR spectroscopy, with mesitylene as an internal standard (7.3–7.7 and 6.7 ppm, respectively). Each polymerization showed an exponential decay in the starting monomer concentration, i.e., [PA] or [PO], against time (Figure S32). The pseudo-first order rates were established by linear relationships of ln([PA]_t_/[PA]_0_) or ln([PO]_t_/[PO]_0_) against time (Figures 6A, S33–S36). Plots of the pseudo-first order rate coefficient *k*_obs_ against [PO]_0_ or [PA]_0_ were linear (Figure 6B,C). The data suggest the rate law is first order in both the concentrations of PO and PA. The relative orders in these monomers are supported by linear fits to plots of ln(*k*_obs_) vs ln[PO]_0_ or ln[PA]_0_ showing gradients (orders) of 0.93 and 1.10, respectively (Figures S35 and S36). Finally, the polymerization half-lives were consistent with the changes in the concentrations of PO or PA, again supporting first-order dependencies of the rates (Figure S37).

The rate dependence on catalyst concentration was investigated by conducting polymerizations in neat epoxide (14.3 M), with [PA] = 1.43 M, at 60 °C and using starting catalyst concentrations between 3.58 and 17.9 mM (Figure S38). The plot of *k*_obs_ against [catalyst]_0_ is best fit linearly, which is consistent with a first order dependence (Figure 6D).

As such, an overall third-order rate law was determined for PO/PA ROCOP catalyzed by L_wide_CoK(OAc)_2_:
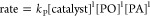


### Polyester Catalytic Cycle and Mechanism

**Figure 7:**
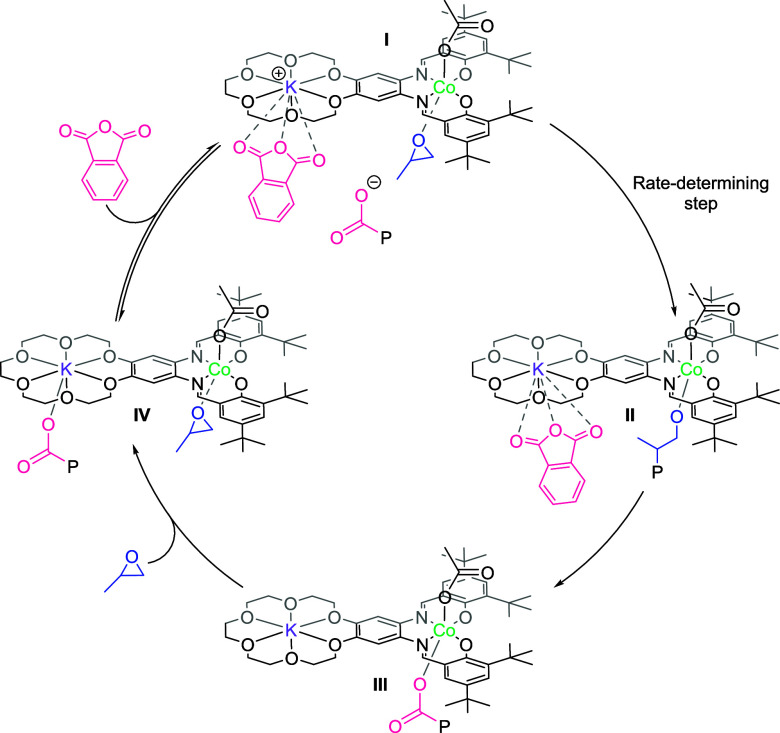
Proposed mechanism for PO/PA ROCOP catalyzed by L_wide_CoK(OAc)_2_.

Following the rate law, it is possible to propose a mechanism for PO/PA ROCOP catalyzed by L_wide_CoK(OAc)_2_. In the mechanism, the rate-determining step is the ring opening of the Co(III)-PO adduct by the carboxylate nucleophile (Figure 7, species **I** to **II**). The rate law also depends upon PA concentration, and this is interpreted by the anhydride playing a key role in activating the catalyst by coordinating to the K center, resulting in the elimination of a highly reactive, anionic carboxylate chain end (Figure 7, species **I**). It is tentatively proposed that there is an equilibrium between the K-carboxylate and the [K-PA]^+^ carboxylate^–^ species (Figure 7, **IV** and **I**, respectively). Perhaps the K-carboxylate intermediate (**IV**) cannot directly attack the Co–PO adduct, as the distance between the electrophile and nucleophile in a single catalyst molecule is too wide. Instead, the PA displaces the carboxylate to form a “free” anionic carboxylate chain end, which attacks and ring-opens the Co(III)–propene oxide. The alkoxide species produced is proposed to rapidly ring-open a phthalic anhydride molecule to form a Co(III)–carboxylate intermediate (**III**), which rearranges to form the K(I)–carboxylate intermediate **IV**.

Considering alternative catalyst speciation, a dimeric catalytic mechanism seems less likely as the catalyst, in the coordinating solvent (MeOD-*d*_4_), shows a single species by DOSY NMR, the diffusion coefficient of which indicates it remains monomeric (Figure S39). The formation of an anionic, free carboxylate intermediate in the rate-determining step may explain the high rates for PO/PA ROCOP, since such species should be strongly nucleophilic compared to metal-bound carboxylates. Indeed, recent studies have proposed that polymerization rates are accelerated by increasingly weak coordination of the anionic chain end to the catalyst species.^6,61^ The existence of an equilibrium between active species **I** and inactive species **IV** might explain the observation that the activity of the catalyst is directly proportional to PA concentration. At high concentrations, the equilibrium is pushed toward the active species, **I**. As the reaction progresses, and PA is consumed, the equilibrium shifts back toward the inactive species **IV**, resulting in the exponential decay of PA concentration that is characteristic of first-order kinetics. UV–vis titration experiments were conducted with the catalyst and increasing concentrations of PA, in THF, using up to 250 000 PA equivalents relative to the catalyst (Figure S41). A gradual change in the absorptions in the UV–vis spectra are seen with increasing concentrations of PA. These data are consistent with a reversible interaction between the catalyst and PA, in solution. The data may support the hypothesis of an equilibrium between intermediates **IV** + PA and **I** + “free carboxylate” in the catalytic cycle. No saturation of the UV–vis spectrum was observed, even at such high loadings of PA, indicating that any equilibrium would lie far toward **IV** + PA.

The narrow separation catalyst shows a different rate law (zero order in anhydride concentration), suggesting it operates by a more standard chain shuttling mechanism, as has been observed for other dinuclear catalysts.^6,17^

### Literature Catalyst Comparison

**Figure 8:**
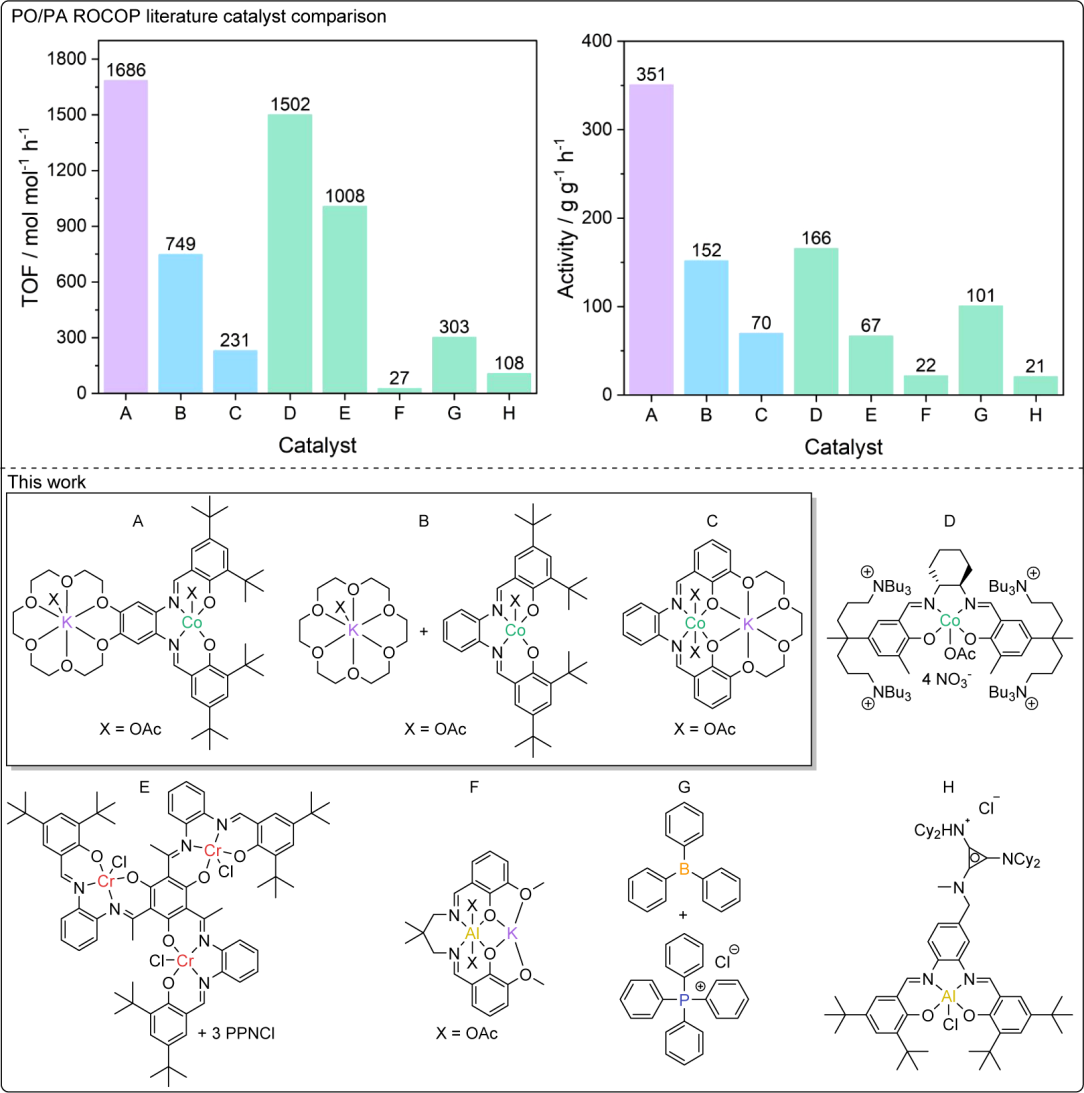
Comparison of the three Co(III)K(I) catalysts against high-performance literature catalysts for PO/PA ROCOP (for conditions, see Figure S40).^7,35,62−64^.

The catalyst activities, in terms of both moles of polymer produced per moles of catalyst per hour (TOF), and gram per gram activity, were evaluated against some of the best catalysts reported in the literature (Figure 8). L_wide_CoK(OAc)_2_ is among the best performing catalysts reported to date, with its TOF of 1686 h^–1^. (Figure 8). As mentioned at the outset, the ROCOP using PO/PA is challenging and most literature catalysts show TOF < 100 h^–1^. Other excellent catalysts include a cobalt salen with four tethered quaternary ammonium “arms”, reported by Lee and coworkers, which shows a molar TOF of 1502 h^–1^, and a trimetallic Cr(III)_3_ Schiff base complex, reported by Lu and coworkers, which shows a molar TOF of 1008 h^–1^ (**D** and **E**, respectively, in Figure 8; 1:242:7508:100 000 [cat]_0_:[EtOH]_0_:[PA]_0_:[PO]_0_, 80 °C, neat; 1:3:600:3000 [cat]_0_:[PPNCl]_0_:[PA]_0_:[PO]_0_, 60 °C, neat).^35,64^ As discussed previously, the tethered ammonium cobalt salen complex is complex to synthesize, and is applied at 80 °C, which would be expected to increase the rate of polymerization significantly.^60,64^ It is also important to consider that the tri-Cr(III) catalyst only shows this high rate when used with 3 equiv of PPNCl. The cocatalyst is not only very expensive but also corrosive and toxic. When considering the activity per gram of catalyst, the high molar masses for these two catalysts is a disadvantage (RMM **D** = 1674 g mol^–1^, **E** = 3107 g mol^–1^ vs 989 g mol^–1^ for L_wide_CoK(OAc)_2_, **A** in Figure 8). As such, the gram per gram activity of L_wide_CoK(OAc)_2_ is twice that of the tethered ammonium cobalt salen catalyst, and five times greater than that of the tris-Cr(III) salen catalyst (351 h^–1^ vs 166 h^–1^ and 351 h^–1^ vs 67 h^–1^, respectively; Figure 8). L_wide_CoK(OAc)_2_ also has superior activities, either mol mol^–1^ h^–1^ or g g^–1^ h^-1^, compared to other excellent catalysts, including the Al(III)K(I) heterodinuclear catalyst (F), the organoborane/phosphonium chloride system (**G**), or the cationic Al(III) Schiff base catalyst (**H**) (Figure 8**F**–**H**; for conditions, see Figure S55).^7,62,63^

The bicomponent catalyst system L_mono_CoKOAc + KOAc[18-crown-6] (**B**) also performs very well in comparison to the literature catalysts. The simplicity of its synthesis might make it an interesting future candidate for PO/PA ROCOP, especially when relatively high catalyst loadings are acceptable.

### Monomer Scope

**Figure 9:**
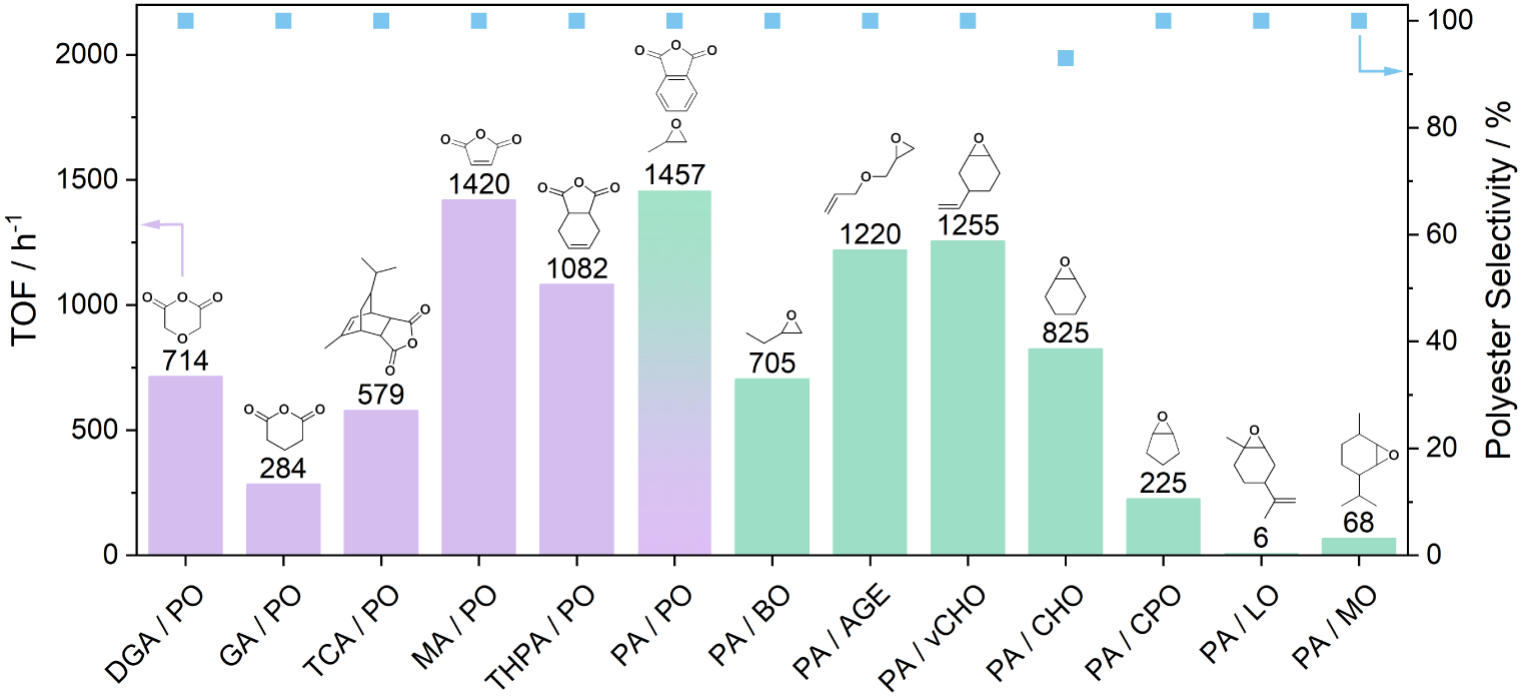
Turnover frequencies (TOFs) and polyester selectivity for polymerizations using different monomer pairs, from LHS reactions combining PO with different anhydride (purple data) while the RHS showing PA with various epoxides (green data).

An attraction of epoxide/anhydride ROCOP is the large number of commercially available epoxides and anhydrides, which could be used to make polyesters with rigid, aromatic, or flexible backbone substituents. It is important to evaluate that the best catalysts are also tolerant of a range of different monomers. L_wide_CoK(OAc)_2_ was tested for the ROCOP of PO with various anhydrides (Figure 9 LHS (purple)) and of PA with various epoxides (Figure 9 RHS (green)). In each case, the polymerizations were conducted with 14.3 mM catalyst, 1.43 M anhydride, 0.29 M BDM, and 0.75 mL of the epoxide at 70 °C. For PO/PA ROCOP, these conditions correspond to 1:20:100:1000 [catalyst]_0_:[BDM]_0_:[PA]_0_:[PO]_0_.

The catalyst was active under all conditions and showed good performance with different anhydrides, including those featuring C3 carboxylate separations (six-membered rings: DGA or GA), C2-carboxylate separations (five-membered rings: TCA, MA, THPA and PA). In general, the five-membered cyclic anhydrides showed faster rates than the six-membered anhydrides. This may be due to the higher anhydride torsion angle, which increases its basicity and shifts the coordination equilibria with K(I) in the rate-determining step. The bioderived tricyclic anhydride, TCA, was least active among the five membered ring anhydrides and is likely due to the increase in sterics. L_wide_CoK(OAc)_2_ was also very effective using different epoxides, including for terminal epoxides (PO, BO), six-membered cyclic epoxides (CHO, vCHO, LO, MO), and for the five-membered cyclic epoxide (CPO). Epoxides with high steric congestion proved less active, for example, slower rates using LO or MO than for CHO or vCHO. Overall, the catalyst is highly active across the range of commonly applied epoxides and anhydrides, and its low temperature activity makes it well-suited to ROCOP using propene oxide.

## Conclusions

A series of three Co(III)/K(I) heterodinuclear catalysts were tested for propene oxide ring-opening copolymerizations with phthalic anhydride or with carbon dioxide. The catalysts feature closely related Schiff base ancillary ligands but have different and controllable intermetallic separations. The catalyst with a wide intermetallic separation, L_wide_CoK(OAc)_2_, showed the highest activity for PO/PA ROCOP, and it also showed quantitative selectivity for ester linkage formation and good polymerization control and impurity tolerance. The same catalyst was, however, nearly completely inactive for PO/CO_2_ ROCOP. In contrast, the catalyst with the narrow intermetallic separation, L_short_CoK(OAc)_2_, showed the highest activity and selectivity for PO/CO_2_ ROCOP, and it was also well controlled and tolerant to impurities but was not very active for PO/PA ROCOP. A bicomponent catalyst system, comprising a monometallic Co(III) complex applied with a potassium salt, showed high activities for both PO/PA and PO/CO_2_ ROCOP but required high catalyst concentrations and was not very effective at low loadings. The bicomponent catalyst may be useful but only for polymerizations where high catalyst loading can be tolerated (i.e., catalyst cost is not a barrier and effective removal strategies are developed).

The L_wide_CoK(OAc)_2_ catalyst rate law was determined to be first order in catalyst, anhydride, and epoxide concentrations. It was also highly effective in the polymerization of a range of different anhydrides and epoxides. The rate data were compared for the two different classes of polymerizations with some useful guiding principles for catalyst design emerging from the investigation. For epoxide/carbon dioxide ROCOP, catalyst separations are most effective in the “shorter” range (3–4 Å) and ancillary ligands that position metals accordingly should be prioritized. For epoxide/anhydride ROCOP, catalysts featuring more widely separately metals (8–9 Å) should be prioritized in future.

## Supporting Information Available

Additional experimental details, materials, methods (kinetics plots), and characterization data (NMR, IR, UV–vis spectroscopies, MALDI-ToF mass spectrometry, crystal structures, cyclic voltammetry, and GPC plots) (PDF)
