# Supplementary material for: Synergic Catalysis: the Importance of Intermetallic Separation in Co(III)K(I) Catalysts for Ring Opening Copolymerizations
Source: J Am Chem Soc. 2024 Aug 9;146(33):23517–28. doi: 10.1021/jacs.4c07405 (PMC11345820; doi:10.1021/jacs.4c07405)
Supplement: Supplementary file 1 — ja4c07405_si_001.pdf [file ja4c07405_si_001.pdf]

Supplementary Information

Synergic Catalysis: The Importance of Intermetallic Separation in Co(III)K(I) Catalysts for Ring Opening Copolymerizations.

Francesca Fiorentini, Katharina H.S. Eisenhardt, Arron C. Deacy, and Charlotte K. Williams*

Department of Chemistry, University of Oxford, OX1 3TA, United Kingdom

* charlotte.williams@chem.ox.ac.uk

Figure S1. Plot of TOF against solid-state, dimeric intermetallic separation of Zn(II) BDI complexes with changing steric bulk.[2] 8
Figure S2. Zn(II) BDI complexes shown in Figure 1, with changing R groups adjacent to N donors in BDI ligands.[2] 8
Figure S3. Structures of high pressure (> 5 bar) CHO/CO_2 ROCOP catalysts used in Figure 1 and Table S1.[2-8] 10
Figure S4. CHO/PA ROCOP catalyst structures used in Figure 1 and Table S2. [6, 9-13] 12
Figure S5. General dinuclear mechanism for epoxide/CO_2 ROCOP (showing PO/CO_2 ROCOP). 13
Figure S6. General dinuclear mechanism for epoxide/anhydride ROCOP (showing PO/PA ROCOP). 13
Figure S7. Synthetic pathway for H_2L_{wide} i) H_2SO_4, HNO_3, $CHCl_3$, 0 °C to rt, 2 days; ii) Pd/C, 1.5 bar H_2, MeOH, rt, 5 days iii) 3,5-ditertbutylbenzaldehyde, MeOH, N_2, 65 °C, 3 days. 14
Figure S8. $L_{wide}CoK(OAc)_2$ synthesis from H_2L_{wide}; i) KOAc, MeOH, N_2, reflux, 1 h, ii) $Co(OAc)_2$, N_2, rt iii) AcOH, MeOH, air, rt, 4 h. 14
Figure S9. ^1H NMR spectrum of $L_{wide}CoK(OAc)_2$ in MeOD-d_4. 15
Figure S10. COSY NMR spectrum of $L_{wide}CoK(OAc)_2$ in MeOD-d_4 15
Figure S11. $^{13}C\{^1H\}$ NMR spectrum of $L_{wide}CoK(OAc)_2$ in MeOD-d_4 16
Figure S12. HSQC NMR spectrum of $L_{wide}CoK(OAc)_2$ in MeOD-d_4 16
Figure S13. HMBC NMR spectrum of $L_{wide}CoK(OAc)_2$ in MeOD-d_4 17
Figure S14. Solid state IR spectrum of $L_{wide}CoK(OAc)_2$ 17
Figure S15. UV-vis spectrum of $L_{wide}CoK(OAc)_2$ in DCM. 18
Figure S16. MALDI-TOF mass spectrometry trace of $L_{wide}CoK(OAc)_2$ 18
Figure S17. Experimental (green) and calculated (black) isotopic distributions of the $[Co(II)K(I)]^+$ peak 19
Figure S18. Stacked ^1H NMR spectra of H_2L_{wide} (top; black) in $CDCl_3$ and $L_{wide}CoK(OAc)_2$ (bottom; green) in MeOD-d_4 showing the disappearance of the phenol peak (dotted box). 19
Figure S19. Overlaid IR spectra of H_2L_{wide} (back) and $L_{wide}CoK(OAc)_2$ (green) showing the disappearance of the phenol O-H stretch upon Co(III) coordination to the pro-ligand. 20
Figure S20. Overlaid UV-vis spectra of H_2L_{wide} (back) and $L_{wide}CoK(OAc)_2$ (green) showing shifting of $\pi \rightarrow \pi^*$, $n \rightarrow \pi^*$, and $d \rightarrow d$ transitions 20
Figure S21. ^1H NMR spectrum of $L_{wide}CoK(OAc)_2$ in MeOD-d_4 between 50 to -50 ppm showing the absence of peaks outside of the typical range. 21
Figure S22. Half-dimer molecular structure of $L_{wide}NiKOAc$ determined by single crystals diffractometry. Solvent molecules and hydrogen atoms omitted for clarity, ellipsoids drawn at 40% probability. 21
Figure S23. Dimeric molecular structure of $L_{wide}NiKOAc$ determined by single crystals diffractometry. Solvent molecules and hydrogen atoms omitted for clarity, ellipsoids drawn at 40% probability. 22
Figure S24. ^1H NMR spectrum of a representative PO/PA ROCOP reaction showing integrals and/or regions for PA monomer, polyester, mesitylene (10 eq. used as an internal standard), and polyether for the calculation of conversion, which is in turn used to calculate the turnover number (TON) and turnover frequency (TOF), and selectivity for polyester. 22
Figure S25. Representative GPCs of polyester produced from PA/PO ROCOP catalysed by $L_{wide}CoK(OAc)_2$ with and without added chain transfer agent. 23
Figure S26. Representative plot of polyester M_n against conversion for PA/PO ROCOP catalysed by $L_{wide}CoK(OAc)_2$. 23
Figure S27. Conversion vs. time plot for PO/PA ROCOP catalysed by bicomponent system $L_{mono}CoOAc$ + KOAc[18-crown-6]. There are two regions to the plot; one with a slow, initial rate, and the other with a faster rate. The initiation time is taken as the time from which the faster rate occurs.24
Figure S28. ^1H NMR spectrum of a representative PO/CO_2 ROCOP reaction showing integrals for mesitylene (10 eq. used as an internal standard), polycarbonate, cyclic carbonate, and polyether for the

calculation of conversion, which is in turn used to calculate the turnover number (TON) and turnover frequency (TOF), and selectivity for CO_2 and polymer. 25
Figure S29. Structures, activites, selectivities and conditions for selected PO/CO_2 ROCOP catalysts.[14-17] 26
Figure S30. Cyclic voltammetry trace of $L_{wide}CoK(OAc)_2$. 26
Figure S31. Cyclic voltammetry trace of $L_{mono}CoOAc$. 27
Figure S32. Structure of the chlorinated analogue of $L_{short}CoK(OAc)_2$....................28
Figure S33. Representative plots of the concentration of PA (LHS) and PO (RHS) over the course of a polymerization (1:100:1000 $[L_{wide}CoK(OAc)_2]_0$:$[PA]_0$:$[PO]_0$ with exponential fits. Indicates first-order dependence on [PA] and pseudo-zero order dependence on [PO] due to the large excess of PO.........28
Figure S34. Plots of $\ln([PA]_t/[PA]_0)$ for varying concentrations of PO showing the increase in rate with increasing PO concentration. 29
Figure S35. Plots of $\ln([PO]_t/[PO]_0)$ for varying concentrations of PA showing the increase in rate with increasing PA concentration. 29
Figure S36. Plot of k_{obs} vs. $[PO]_0$ showing a linear dependence. 30
Figure S37. Plot of k_{obs} vs. $[PA]_0$ showing a linear dependence. 30
Figure S38. Graphs showing consistent polymerisation half-life with changing concentrations of PA and PO, supporting first-order dependencies of rate on the concentration of each monomer..............31
Figure S39. Plots of $\ln([PA]_t/[PA]_0)$ for varying concentrations of $L_{wide}CoK(OAc)_2$ showing the increase in rate with increasing $L_{wide}CoK(OAc)_2$ concentration. 31
Figure S40. DOSY NMR spectrum of $L_{wide}CoK(OAc)_2$ in MeOD-d_4. 32
Figure S41. Stacked UV-vis spectra of $L_{wide}CoK(OAc)_2$ with increasing concentration of PA (from 0 (red) – 250,000 (blue) equivalents). Initial concentration of $L_{wide}CoK(OAc)_2$ = 0.175 µM in THF.....32
Figure S42. Literature catalysts for PO/PA ROCOP used for comparison.[6, 10, 18, 19] 33

Experimental

General Methods

All manipulations were performed using either a dual manifold nitrogen-vacuum Schlenk line or a nitrogen-filled glovebox. Solvents were collected from a solvent purification system, degassed using three freeze-pump-thaw cycles and stored over 3 Å molecular sieves in a nitrogen-filled glovebox. All reagents were obtained from commercial sources and used as received. Propene oxide monomer was bought from Sigma Aldrich and dried overnight over calcium hydride and purified by fractional distillation, followed by degassing with nitrogen and stored under nitrogen. Phthalic anhydride was bought from Sigma Aldrich and purified by stirring in dry toluene overnight. The supernatant was removed by filtration and the toluene removed *in vacuo*. The resultant white solid was recrystallised from hot chloroform and sublimed under vacuum at 80 °C. Research-grade CO_2 (BOC, 99.99 %) was used and dried through a Drierlite column and two additional drying columns (Micro Torr, model number: MC1-804FV) in series before use.

NMR spectroscopic analysis was performed using a Bruker AV 400 MHz spectrometer at 298 K. Gel permeation chromatography (GPC) analysis was performed using a Shimadzu LC-20AD instrument, at 40 °C, with two mixed bed PSS SDV linear S columns in series, and with THF as eluent at a flow rate of 1 mL/min. Molar mass values were calibrated using narrow molar mass polystyrene standards. UV-visible spectra were collected using a Cary 60 UV-Vis spectrophotometer (Agilent Technologies)

Epoxide/Anhydride ROCOP

A solution of catalyst (0.014 mmol) and anhydride (0.143 mmol) in epoxide (1 mL) was prepared in a dried vial, inside a N_2 glovebox. The vial was sealed, with a melamine cap containing a Teflon inlay, and further sealed with electrical insulation tape. The sealed vial was then heated to the desired temperature for the time stated. Aliquoting was performed by cooling the polymerization vial in an acetone/dry ice bath, before removing ~ 10 µL of the mixture with a pipette in a N_2 glovebox. Aliquots were analysed by ^{1}H NMR spectroscopy ($CDCl_3$) to determine conversion, and by GPC in THF to determine the molar mass and dispersity of the resultant polyester. The polymerizations were quenched by addition of benzoic acid (2 mg, 0.018 mmol), and the polyesters purified by precipitation from chloroform with methanol and dried under vacuum. Polymerizations were run in triplicate to allow determination of errors.

Epoxide/CO_2 ROCOP

A solution of catalyst (0.018 mmol), *1,2*-cyclohexane diol (42 mg, 0.357 mmol), and mesitylene (25 µL, 0.179 mmol, internal standard) in PO (5 mL, 71.5 mmol) was prepared in a N_2 glovebox. The solution was injected into a 100 mL Parr reactor, fitted with a DiComp sentinel probe attached to an ATR-IR spectrometer, under a stream of CO_2. The reactor was pressurized to 20 bar CO_2 pressure and heated to 50 °C. Conversion to polycarbonate (PPC) and cyclic carbonate (PC) were followed using *in situ* ATR-IR spectroscopy by observing the development of peaks at 1750 cm^{-1} and 1810 cm^{-1} for PPC and PC, respectively. The copolymerization was stirred for the reaction duration, allowed to cool upon completion, and quenched by addition of benzoic acid (2 mg, 0.018 mmol). Conversions to PPC, PC and polyether (PPO) were determined by ^{1}H NMR spectroscopy of the crude sample, using mesitylene as an internal standard, and molar masses and dispersities of resultant PPC determined by GPC in THF. The polymer product was purified by dissolving in chloroform and precipitating with methanol. Polymerizations were run in triplicate to allow determination of errors.

Synthesis

Dinitrobenzo[18-crown-6]

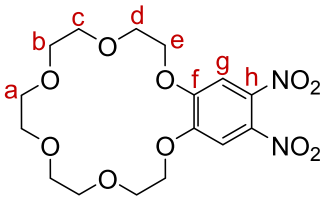

To a solution of benzo-18-crown-6 (3.00 g, 9.62 mmol) in chloroform (80 mL) cooled to 0 °C was added a mixture of sulphuric acid (>95%, 10 mL) and nitric acid (70%, 20 mL) dropwise over an hour with rapid stirring. The solution gradually turned amber and nitrogen dioxide evolved. The solution was then stirred at room temperature for 48 h. Upon completion, the reaction was diluted with chloroform (60 mL) and washed with ice-cooled distilled water (3 x 40 mL). The combined aqueous phases were back-extracted with chloroform (40 mL). The organic phases were then combined and dried ($MgSO_4$), before concentrating to 10 mL *in vacuo*. Diethyl ether (approximately 200 mL) was added and the solution cooled at -12 °C for 2 h. The precipitated product was filtered from the solution and washed with cold diethyl ether, giving pure dinitrobenzo-18-crown-6 as a pale-yellow solid (3.24 g, 8.06 mmol, 84%). ^{1}H NMR (400 MHz, $CDCl_3$, 298 K) δ (ppm): 7.27 (s, 2H, Ar**H**, **g**), 4.19 – 4.24 (m, 4H, C**H₂**, **e**), 3.85 – 3.90 (m, 4H, C**H₂**, **d**), 3.65 – 3.70 (m, 4H, C**H₂**, **c**), 3.60 – 3.64 (m, 4H, C**H₂**, **b**), 3.58 (s, 4H, C**H₂**, **a**); ^{13}C {^{1}H} NMR (151 MHz, CD_3CN, 298 K) δ (ppm): 151.6 (**C**O, **f**), 136.7 (**C**NO_2, **h**), 108.4 (**C**H, **g**), 71.1 (**b**), 70.8 (**C**H_2, **a**), 70.5 (**C**H_2, **c**), 69.9 (**C**H_2, **e**), 68.9 (**C**H_2, **d**).

H_2L_{wide}

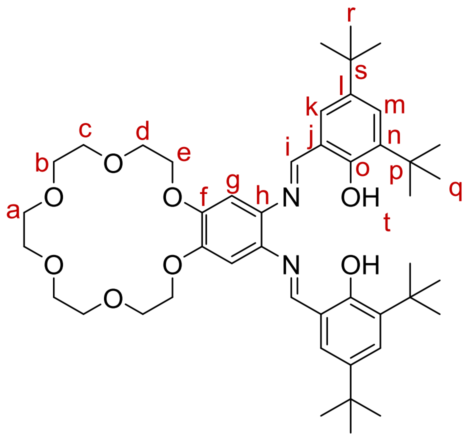

A solution of dinitrobenzo-18-crown-6 (7.00 g, 17.4 mmol) and Pd/C (10 w%, 0.46 g, 4.4 mmol) in degassed methanol (200 mL) was stirred in the dark under 1.5 bar H_2 at room temperature for 5 days. The resultant yellow solution of diaminobenzo-18-crown-6 was filtered via cannula into an ampoule containing 3,5-ditertbutyl 1-hydroxybenzaldehyde (8.20 g, 34.8 mmol). The resultant solution was stirred in the dark at 65 °C for 3 days. The solution immediately darkened upon heating, and eventually a yellow precipitate formed. The reaction was cooled and filtered in air, and the precipitate washed with cold methanol. The pure product H_2L_{wide} was obtained as a bright yellow solid (11.73 g, 15.1 mmol, 87%). ^{1}H NMR (400 MHz, $CDCl_3$, 298 K) δ (ppm): 13.51 (s, 2H, O**H**, **t**), 8.55 (s, 2H, N=C**H**, **i**), 7.35 (d, 2H, $^4J_{H-H}$ = 2.43 Hz, Ar**H**, **m**), 7.12 (d, 2H, $^4J_{H-H}$ = 2.34 Hz, Ar**H**, **k**), 6.76 (s, 2H, Ar**H**, **g**), 4.19 (t, 4H, $^3J_{H-H}$ = 4.54 Hz, C**H₂**, **e**), 3.90 (t, 4H, $^3J_{H-H}$ = 4.50 Hz, C**H₂**, **d**), 3.70 – 3.76 (m, 4H, C**H₂**, **c**), 3.65-3.69 (m, 4H, C**H₂**, **b**), 3.64 (s, 4H, C**H₂**, **a**), 1.36 (s, 18H, C(C**H₃**)₃, **q**), 1.25 (s, 18H, C(C**H₃**)₃, **r**); ^{13}C {^{1}H} NMR (151 MHz, CD_3CN, 298 K) δ (ppm): 163.5 (s, N=CH, **i**), 158.6 (s, *ipso*-**C**, **o**), 148.6 (s,

H_2CO-**C**, **f**), 140.5 (s, *para*-**C**-C(CH_3)$_3$, **l**), 137.3 (s, *ortho*-**C**-C(CH_3)$_3$, **n**), 136.1 (s, HC=N-**C**, **h**), 128.1 (s, *meta*-CH, **m**), 126.8 (s, *meta*-CH, **k**), 118.6 (s, *ortho*-**C**, **j**), 106.0 (s, **C**H, **g**), 71.1 (s, **C**H_2, **b** or **c**), 71.0 (s, **C**H_2, **a** and **b** or **c**), 69.8 (s, **C**H_2, **d** or **e**), 69.7 (s, **C**H_2, **d** or **e**), 35.3 (s, **C**(CH_3)$_3$, **p**), 34.4 (s, C(**C**H_3)$_3$, **r**), 31.7 (s, **C**(CH_3)$_3$, **s**), 29.6 (s, C(**C**H_3)$_3$, **q**); λ_{max} (DCM)/nm 280, 308sh, 357, 391sh (ε / dm^3 mol^{-1} cm^{-1} 13500, 8200, 12200, 8900); ν_{max}/cm^{-1} 3590 (phenol O-H stretch), 2912 (C-H stretch), 1613 (imine C=N stretch), 1198 (aryl alkyl ether C-O stretch), 1121 (aliphatic ether C-O stretch). Anal. Calc. for $C_{46}H_{66}N_2O_8$: C 71.29, H 8.58, N 3.61%.

L_{wide}CoK(OAc)$_2$

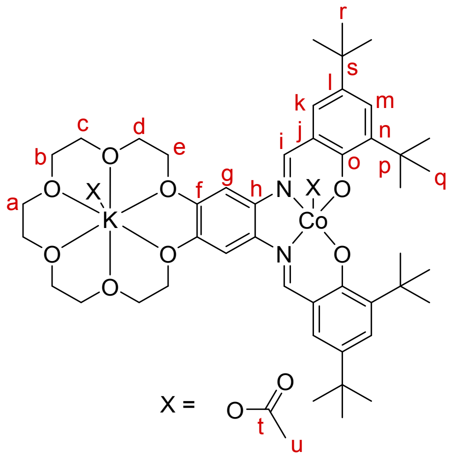

Co(OAc)$_2$ (114 mg, 0.65 mmol), KOAc (63 mg, 0.65 mmol), and the salphen[18-crown-6] ligand (**D**; 500 mg, 0.65 mmol) were combined in dry MeCN (15 mL) under N_2, forming a deep red-brown suspension, which was stirred for 16 h. The solution was then exposed to air, and acetic acid (74 µL, 1.30 mmol) added to the mixture. The solution was stirred for 4 h, over the course of undissolved species dissolved, and the solution turned a deep, vibrant red. Solvent was removed *in vacuo* and the resultant crude product was purified by precipitation from toluene with pentane. The final product was isolated by filtration as a red solid (400 mg; 62% yield). ^{1}H NMR (400 MHz, MeOD-d$_4$, 298 K) δ (ppm): 8.03 (s, 2H, N=C**H**, **i**), 7.73 (s, 2H, Ar**H**, **m** or **k**), 7.68 (s, 2H, Ar**H**, **m** or **k**), 7.63 (s, br, 2H, Ar**H**, **g**), 4.55 (m, 4H, C**H$_2$**, **e**), 4.03 (m, 4H, C**H$_2$**, **d**), 3.80 (m, 4H, C**H$_2$**, **c**), 3.74 (m, 4H, C**H$_2$**, **b**), 3.70 (s, 4H, C**H$_2$**, **a**), 1.91 (s, 8H, O=C-C**H$_3$**, **q** or **r**) 1.77 (s, 18H, C(C**H$_3$**)$_3$, **q** or **r**), 1.40 (s, 18H, C(C**H$_3$**)$_3$, **q** or **r**); ^{13}C{^{1}H} (600 MHz, MeOD-d$_4$, 298 K) 164.96 (s, **l**), 161.90 (s, **t**), 158.24 (s, **g**), 148.78 (s, **f**, **h**), 144.71 (s, **o**), 139.84 (s, **n**), 130.15 (s, **m**), 128.24 (s, **k**), 122.69 (s, **j**), 100.80 (s, **i**), 71.45 (s, **c**), 71.13 (s, **a**), 71.07 (s, **b**), 70.07 (s, **d**), 69.62 (s, **e**), 37.00 (s, **p**), 34.11 (s, **s**), 32.57 (s, **r**), 31.71 (s, **u**), 31.33 (s, **q**); λ_{max} (DCM)/nm 270, 301sh, 358, 402, 422sh, 512 (ε / dm^3 mol^{-1} cm^{-1} 19900, 10200, 6800, 6400, 3900); ν_{max}/cm^{-1} 2906 (C-H stretch), 1609 (imine C=N stretch), 1589 (acetate asymmetric C-O stretch), 1340 (acetate symmetric C-O stretch), 1193 (aryl alkyl ether C-O stretch), 1116 (aliphatic ether C-O stretch); MS (MALDI+) *m/z*: 870.07 [L_{wide}Co(II)K]$^+$; Anal. Calc. for $C_{50}H_{70}CoKN_2O_{12}$: C 60.71, H 7.13, N 2.83 %; Found: C 58.62*, H 6.97, N 2.66%.

* Low C percentage for elemental analysis is proposed to be due to the formation of metal carbides.[1]

L$_{mono}$CoOAc

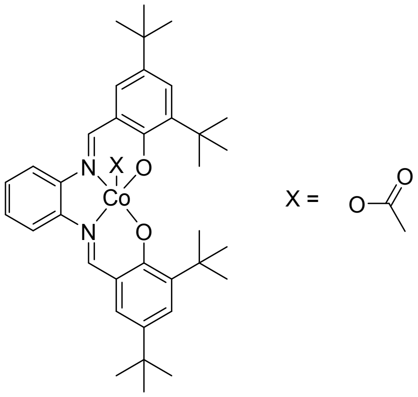

H_2L_{mono} (1.00 g, 1.85 mmol) was combined with methanol (50 mL) and refluxed under N_2 for 2 h. $Co(OAc)_2.4H_2O$ (0.46 g, 1.85 mmol) was added under a flow of N_2. The resultant red solution was refluxed for a further 1 h. Solvent was evaporated *in vacuo* and the resultant solid was redissolved in DCM. To this solution, acetic acid (0.11 mL, 1.85 mmol) was added and stirred open to air for 16 h. The solvent was evaporated *in vacuo* and azeotropic distillations performed on the solid with toluene (3 x 30 mL) and pentane (3 x 30 mL). L$_{mono}$CoOAc was isolated as a bright-red solid (1.12 g, 1.70 mmol, 92%); MS (MALDI+): 599.845 $[L_{mono}Co(II)]^+$ Anal. Calc. for $C_{38}H_{49}CoN_2O_4$: C 69.50, H 8.95, N 5.18 %; Found: C 69.83, H 7.49, N 4.12%.

L$_{short}$CoK(OAc)$_2$

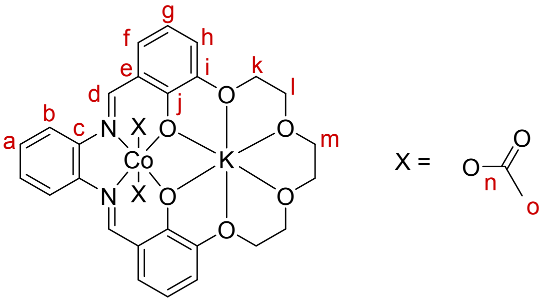

The dialdehyde proligand (300 mg, 0.77 mmol), $Co(OAc)_2$ (136 mg, 0.77 mmol) and KOAc (75 mg, 0.77 mmol) were combined with dry, degassed acetonitrile (15 mL) and stirred under a N_2 atmosphere for 1 h. *ortho*-Phenylene diamine (83 mg, 0.77 mmol) was added in one portion in a N_2 glovebox and the solution was stirred for 16h, before opening to air and adding acetic acid (44 µL, 0.77 mmol). The solution was stirred, open to air, for 3 days. Solvent was removed *in vacuo* and azeotropic distillations performed on the solid with toluene (3 x 30 mL) and pentane (3 x 30 mL). The complex was purified by precipitation (DCM/pentane), and L$_{short}$CoK(OAc)$_2$ was isolated as a brown powder (334 mg, 0.49 mmol, 64%). ^{1}H NMR (400 MHz, $CDCl_3$, 298 K) δ (ppm): 8.19 (s, 2H, N=C**H**, **d**), 7.99 – 8.04 (m, 2H, Ar**H**, **a** or **b**), 7.34 – 7.40 (m, 2H, Ar**H**, **a** or **b**), 7.00 (dd, 2H, $^3J_{H-H}$ = 8.11 Hz, $^4J_{H-H}$ = 1.16 Hz, Ar**H**, **f** or **h**), 6.74 (dd, 2H, $^3J_{H-H}$ = 7.78 Hz, $^4J_{H-H}$ = 1.02 Hz, Ar**H**, **f** or **h**), 6.47 (t, 2H, $^3J_{H-H}$ = 7. 87 Hz, Ar**H**, **g**), 4.18 – 4.24 (m, 4H, O-C**H$_2$**, **k**), 3.95 – 4.00 (m, 4H, O-C**H$_2$**, **l**), 3.86 (s, 4H, O-C**H$_2$**, **m**), 1.38 (s, 6H, OC(O)C**H$_3$**, **o**); ^{13}C{^{1}H} NMR (500 MHz, $CDCl_3$, 298 K) δ (ppm): 178.75 (s, **n**), 157.64 (s, **d**), 156.09 (s, **j**), 150.86 (s, **i**), 145.94 (s, **c**), 126.16 (s, **a**), 117.26 (s, **e**), 114.42 (s, **b**), 112.00 (s, **f** and **h**, or **g**), 111.98 (s, **f** and **h**, or **g**), 69.05 (s, **k**, **l**, or **m**), 68.16 (s, **k**, **l**, or **m**), 65.18 (s, **k**, **l**, or **m**), 23.42 (s, **o**); MS (MALDI+): 561.012 $[L_{short}Co(II)K]^+$; Anal. Calc. for $C_{30}H_{40}CoKN_2O_{10}$: C 53.26, H 4.47, N 4.14 %; Found: C 53.02, H 4.12, N 4.17%.

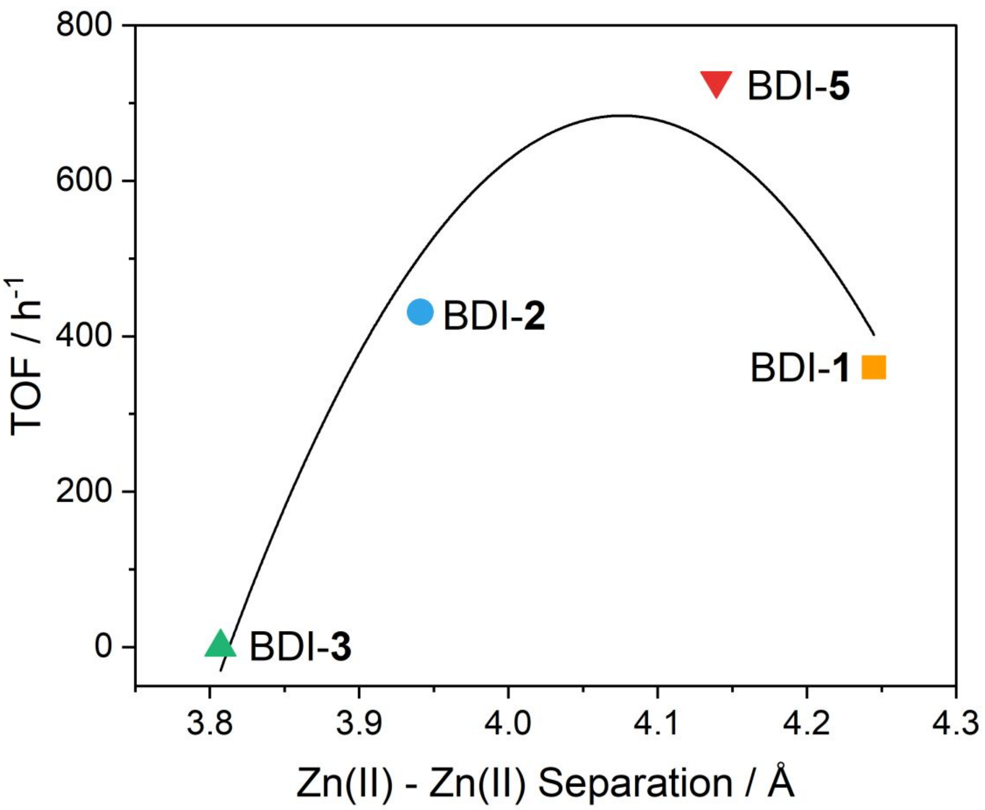

Figure S1. Plot of TOF against solid-state, dimeric intermetallic separation of Zn(II) BDI complexes with changing steric bulk.[2]

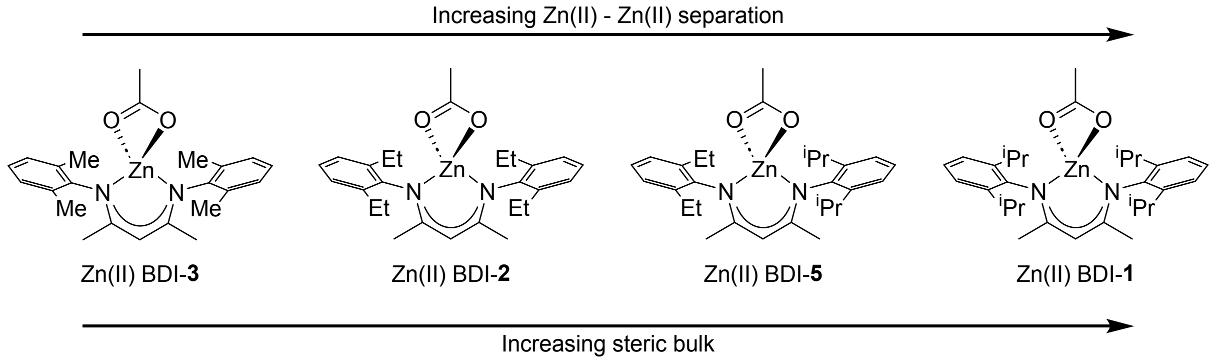

Figure S2. Zn(II) BDI complexes shown in Figure 1, with changing R groups adjacent to N donors in BDI ligands.[2]

Table S1. Catalysts for high pressure (>5 bar) CHO/CO_2 ROCOP used for Figure 1, showing the TOF, intermetallic separation (from solid-state structures), and polymerisation conditions.

Catalyst	TOF / h^{-1}	Solid-State M – M Separation / Å	[CHO]/[cat]	[CHO]	Additives?	CO_2 pressure / bar	Temp. / °C
Zn(II) BDI-**1**[2]	360	4.24	1,000	Neat	No	7	50
Zn(II) BDI-**2**[2]	431	3.94					
Zn(II) BDI-**4**[2]	622	4.09					
Zn(II) BDI-**6**[2]	729	4.14					
Zn(II) BDI-**7**[2]	917	3.85					
Di-Zn(II) Tethered BDI[3]	5880	7.70	4,000	Neat	No	30	100
Di-Co(III) **1a**[4]	1269	7.89*	1,000	Neat	PPNX 2 equiv. to catalyst	20	25
Di-Co(III) **1b**[4]	1356	7.89*					
Di-Co(III) **1c**[4]	1409	7.89*					
Di-Zn(II) anilido-aldimine [5]	2860	4.86	100,000	Neat	No	14	80
Al(III)K(I) *o*-vanillin [6]	505	3.67	2000	Neat	No	20	100
Tri-Co(III)Ce(III)[7]	850	3.68	21,250	Neat	No	20	130
Tri-Co(III)Nd(III) [7]	1625	3.67*					
Tri-Co(III)Sm(III)[7]	488	3.66					
Tri-Co(III)Eu(III)[7]	288	3.66					
Tri-Co(III)La(III)[7]	1375	3.68					
Co(II)Mg(II) [8]	3100	3.04	4000	Neat	1,2-cyclohexane diol 20 equiv. to catalyst	20	100

* When intermetallic separations for the catalyst are not available (i.e. no reported molecular structure available), intermetallic separations for related systems (e.g. same ligand but different metals) are used as an approximation.

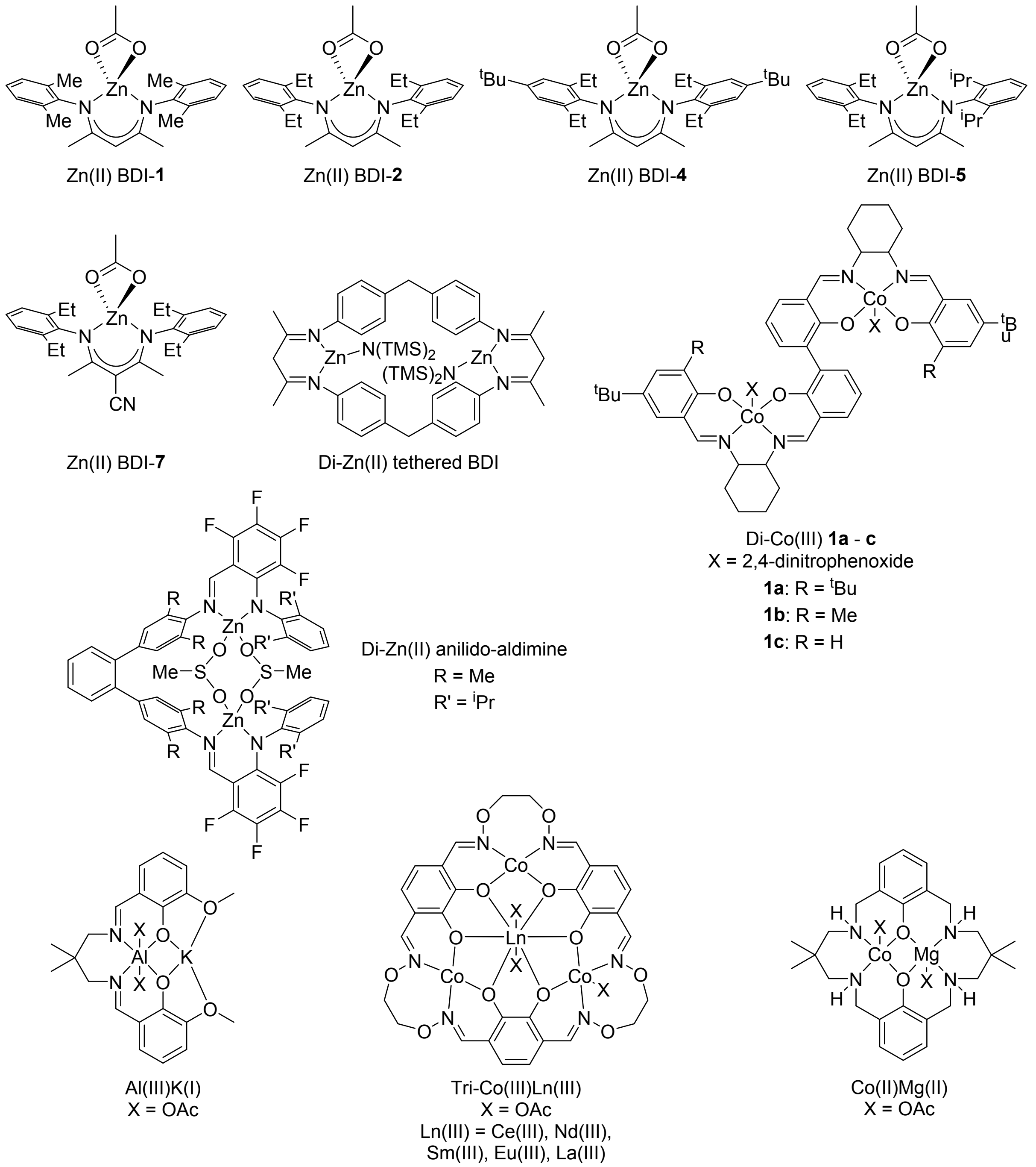

Figure S3. Structures of high pressure (> 5 bar) CHO/CO_2 ROCOP catalysts used in Figure 1 and Table S1.[2-8]

Table 2. Catalysts CHO/PA ROCOP used for Figure 1, showing the TOF, intermetallic separation (from solid-state structures), and polymerisation conditions.

Catalyst	TOF / h^{-1}	Solid-State M – M Separation / Å	[CHO]/[cat]	[CHO]	[PA]/[cat]	Additives?	Temp. / °C
Di-Al(III) **1** [9]	750	7.89*	1,000	Neat	250	PPNCl 2 equiv. to catalyst	50
Di-Co(III) **1** [9]	539	7.89*					
Di-Cr(III) **1** [9]	196	7.89*					
Di-Cr(III) **2** [10]	912	7.32	2,000	Neat	400	PPNCl 1 equiv. to catalyst	60
Tri-Cr(III) **1** [10]	2556	7.36	4,000	Neat	800	PPNCl 1 equiv. to catalyst	60
Tri-Cr(III) **2** [10]	10620	8.47	30,000	Neat	6,000	PPNCl 3 equiv. to catalyst	100
Di-Zn(II) *o*-vanillin [11]	198	3.1557	800	Neat	100	No	100
Fe(III)K(I) *o*-vanillin [12]	1168	3.618	8,000	Neat	400	No	100
Di-Zn(II)Na(I) [13]	225	3.45	4,000	Neat	200	1,2-cyclohexane diol 20 equiv. to catalyst	100
Al(III) Na(I) *o*-vanillin [6]	1072	3.665	2,000	Neat	400	No	100
Al(III) K(I) *o*-vanillin [6]	1136	3.819					
Al(III) Rb(I) *o*-vanillin [6]	712	3.284					
Al(III) Cs(I) *o*-vanillin [6]	875	3.979					

* When intermetallic separations for the catalyst are not available (i.e. no reported molecular structure available), intermetallic separations for related systems (e.g. same ligand but different metals) are used as an approximation.

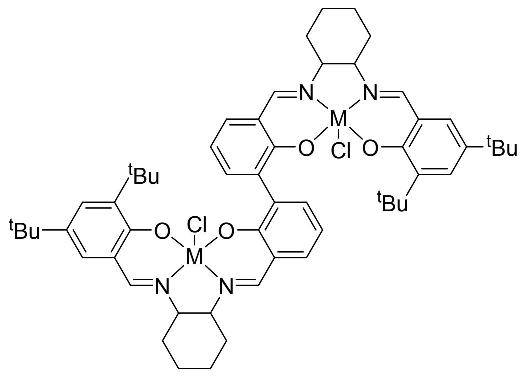

Di-M(III) 1
M = Al(III), Co(III), Cr(III)

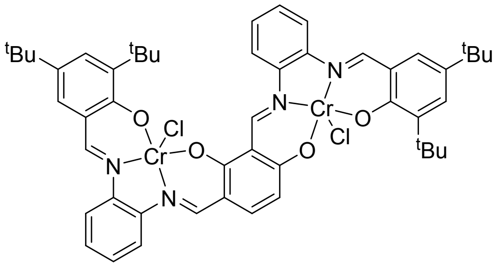

Di-Cr(III) **2**

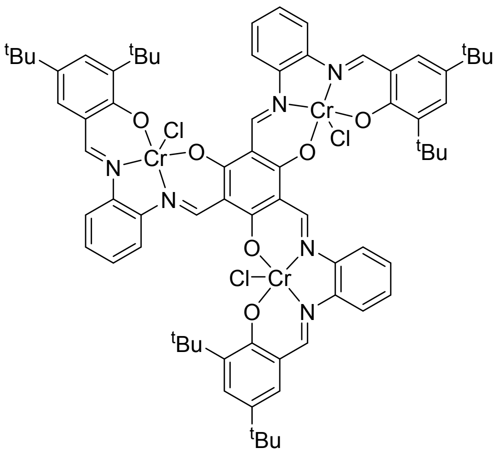

Tri-Cr(III) **1**

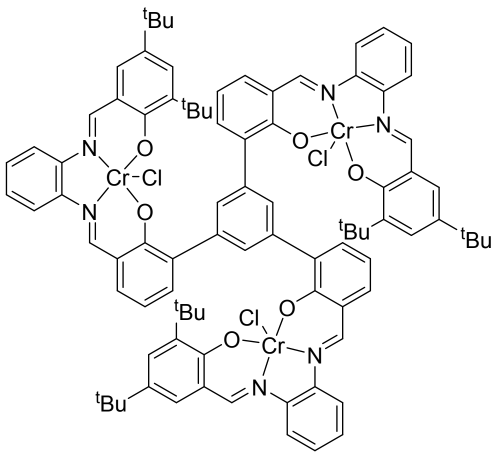

Tri-Cr(III) **2**

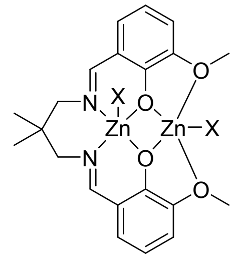

Di-Zn(II) *o*-vanillin
X = OAc

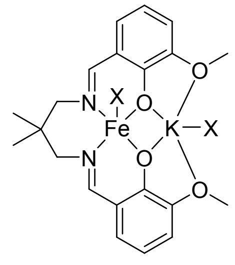

Fe(III)K(I) *o*-vanillin
X = OAc

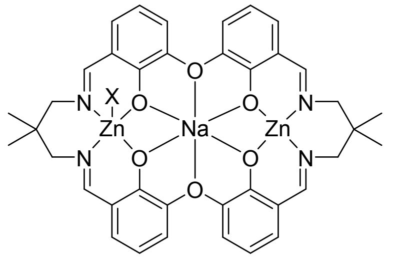

Di-Zn(II)Na(I)
X = $CO_2C_6H_4CF_3$

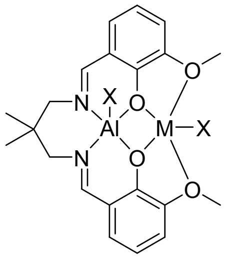

Al(III)M(I) *o*-vanillin
M(I) = Na(I), K(I),
Rb(I), Cs(I)
X = OAc

Figure S4. CHO/PA ROCOP catalyst structures used in Figure 1 and Table S2. [6, 9-13]

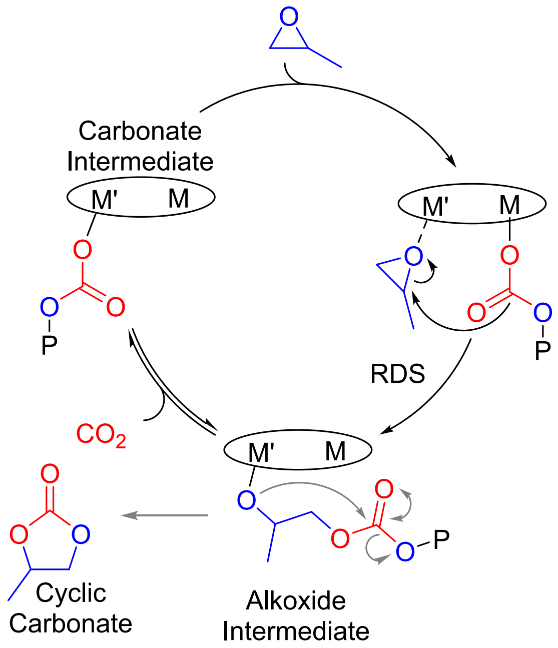

Figure S5. General dinuclear mechanism for epoxide/CO_2 ROCOP (showing PO/CO_2 ROCOP).

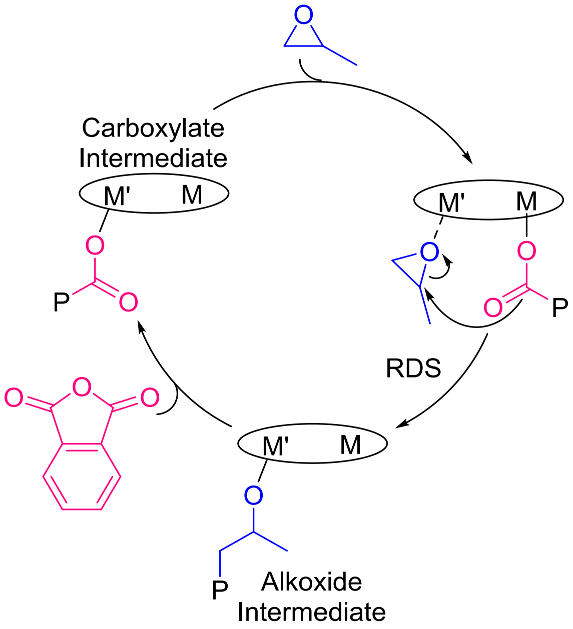

Figure S6. General dinuclear mechanism for epoxide/anhydride ROCOP (showing PO/PA ROCOP).

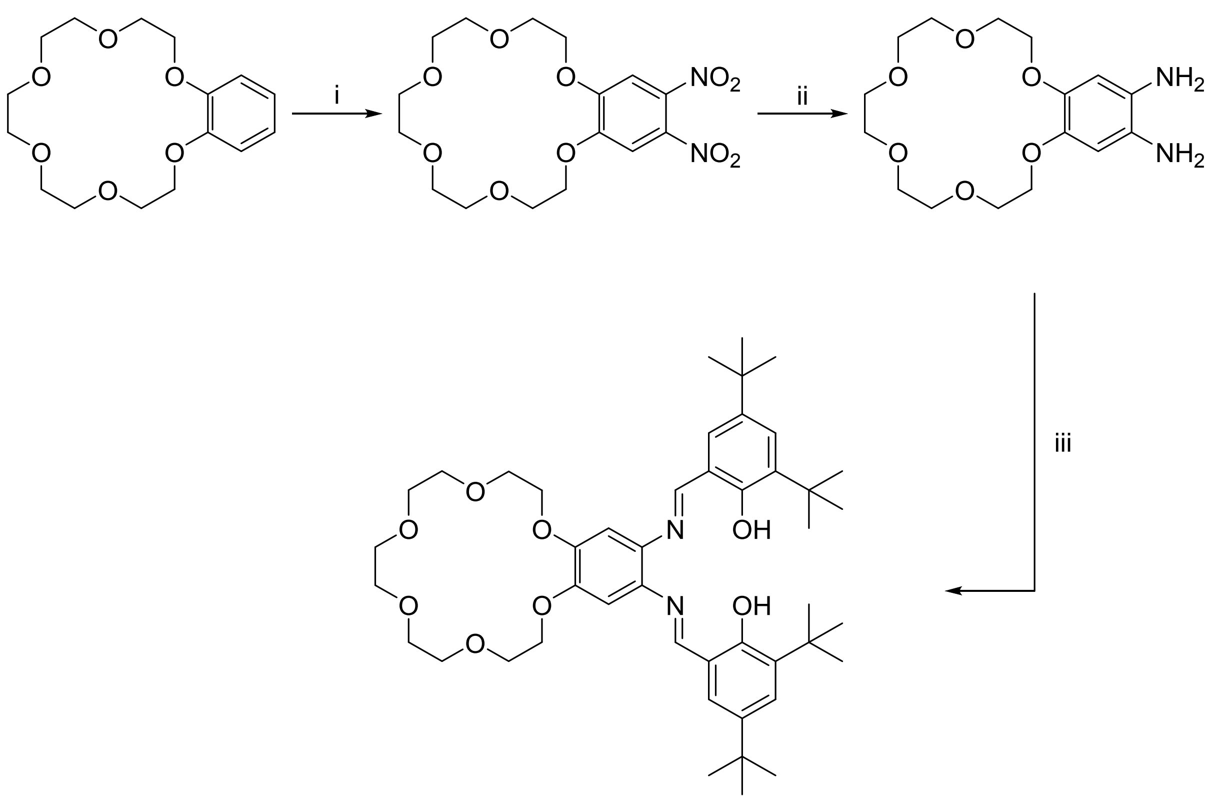

Figure S7. Synthetic pathway for H_2L_{wide} i) H_2SO_4, HNO_3, $CHCl_3$, 0 °C to rt, 2 days; ii) Pd/C, 1.5 bar H_2, MeOH, rt, 5 days iii) 3,5-ditertbutylbenzaldehyde, MeOH, N_2, 65 °C, 3 days.

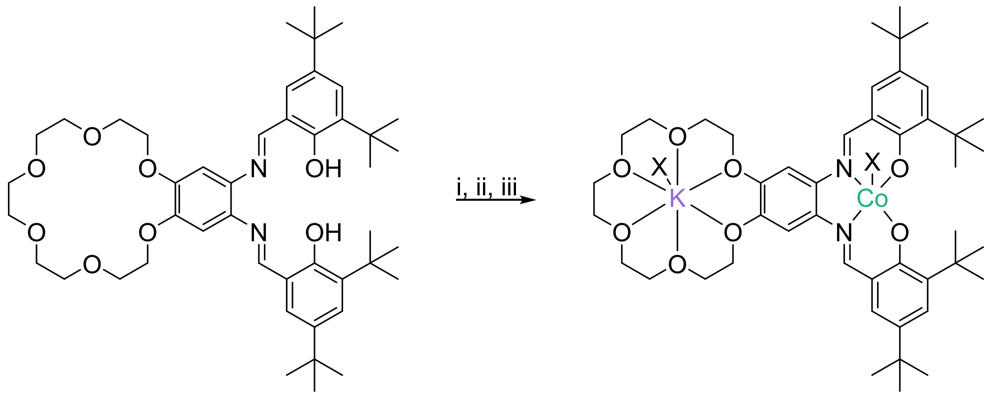

H_2L_{wide}

$H_2L_{wide}CoK(OAc)_2$
X = OAc

Figure S8. $L_{wide}CoK(OAc)_2$ synthesis from H_2L_{wide}; i) KOAc, MeOH, N_2, reflux, 1 h, ii) $Co(OAc)_2$, N_2, rt iii) AcOH, MeOH, air, rt, 4 h.

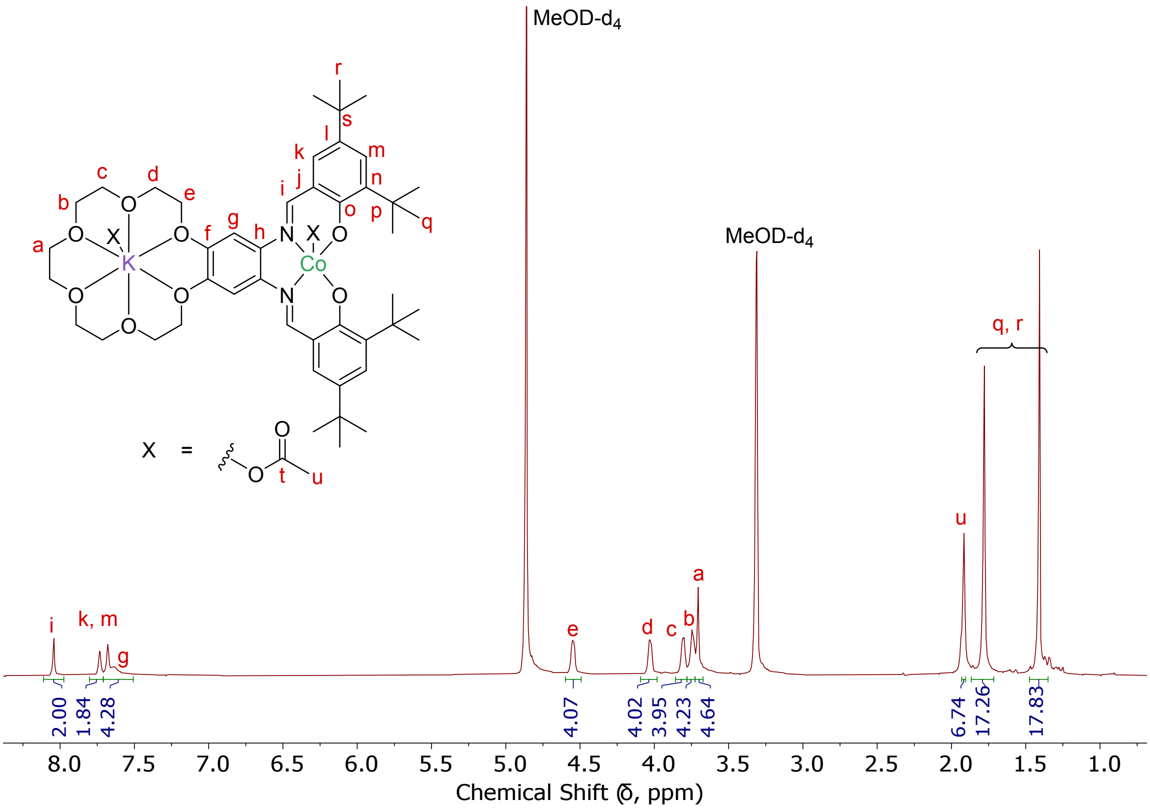

Figure S9. ^{1}H NMR spectrum of $L_{wide}CoK(OAc)_2$ in MeOD-d$_4$.

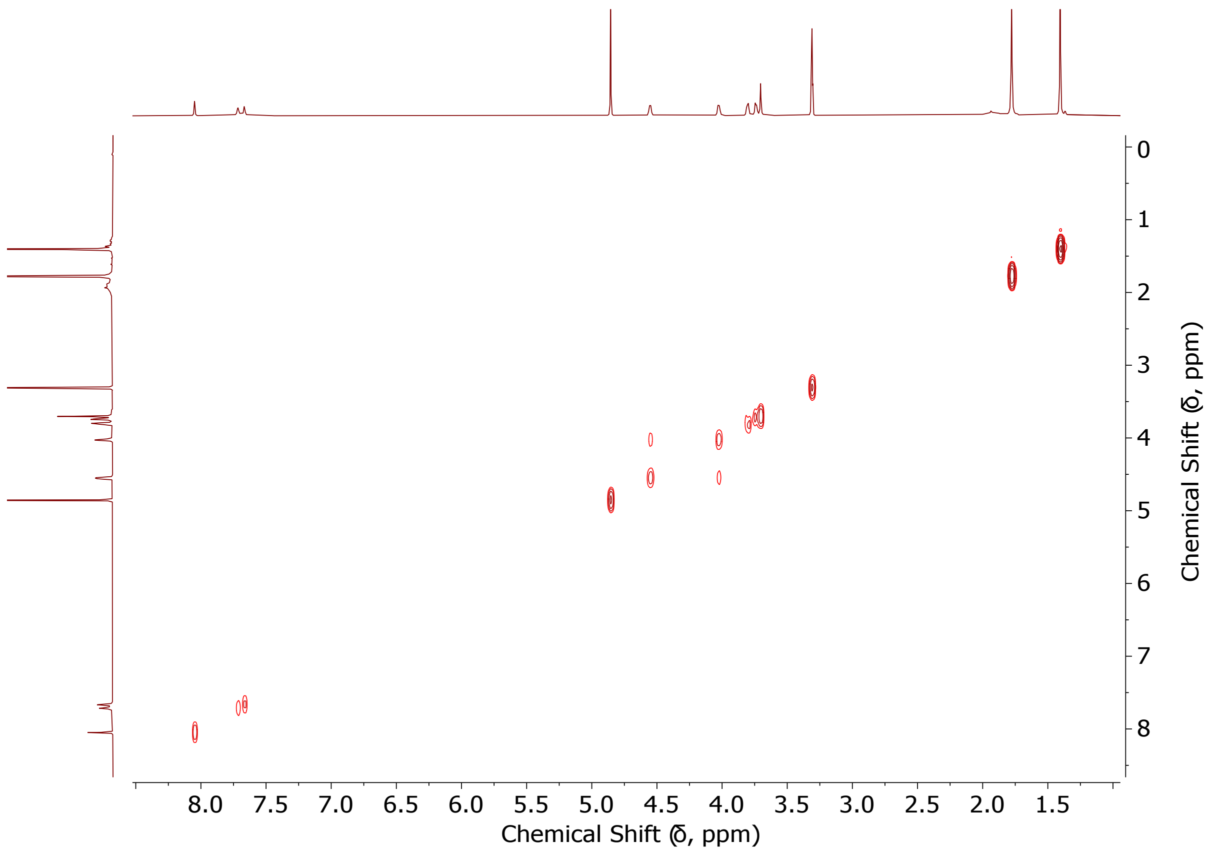

Figure S10. COSY NMR spectrum of $L_{wide}CoK(OAc)_2$ in MeOD-d$_4$

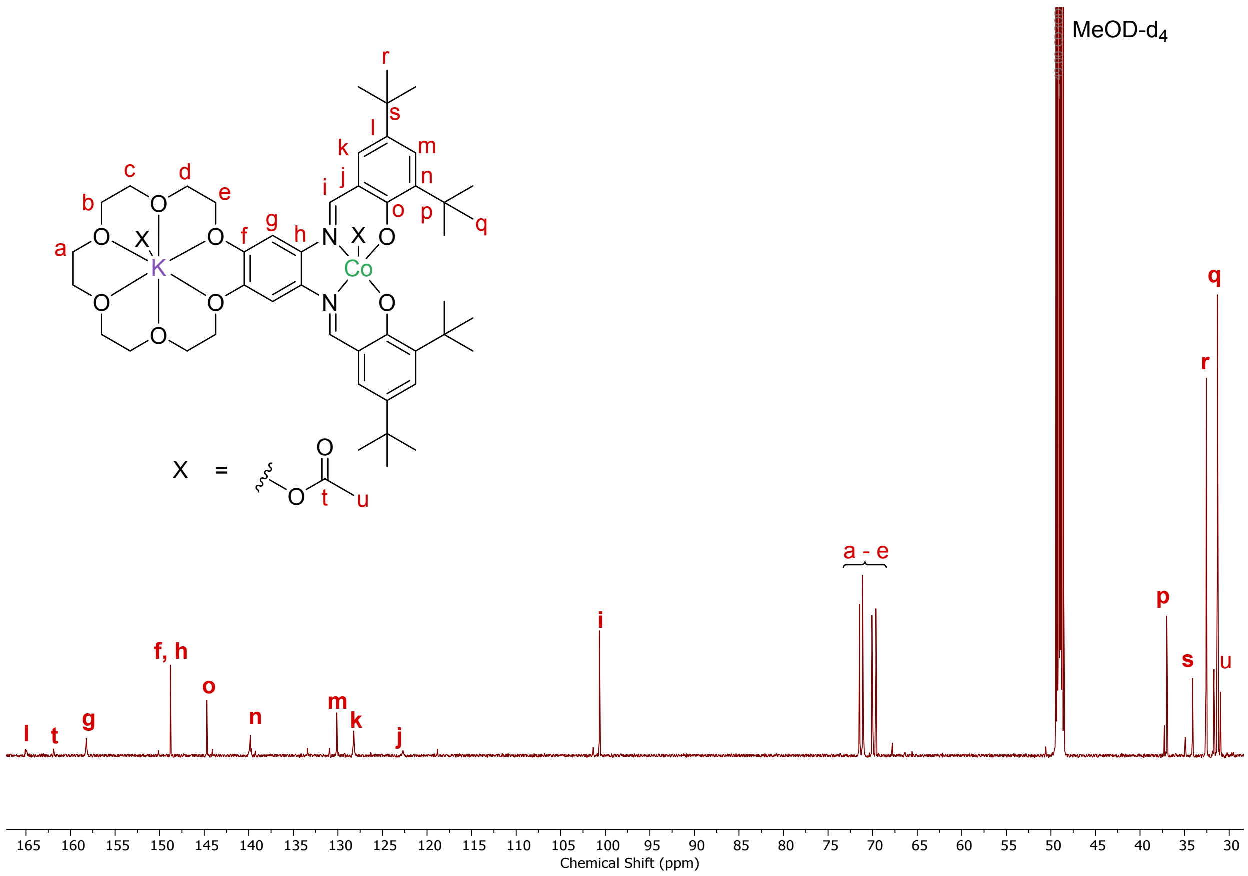

Figure S11. $^{13}C\{^{1}H\}$ NMR spectrum of $L_{wide}CoK(OAc)_2$ in MeOD-d_4

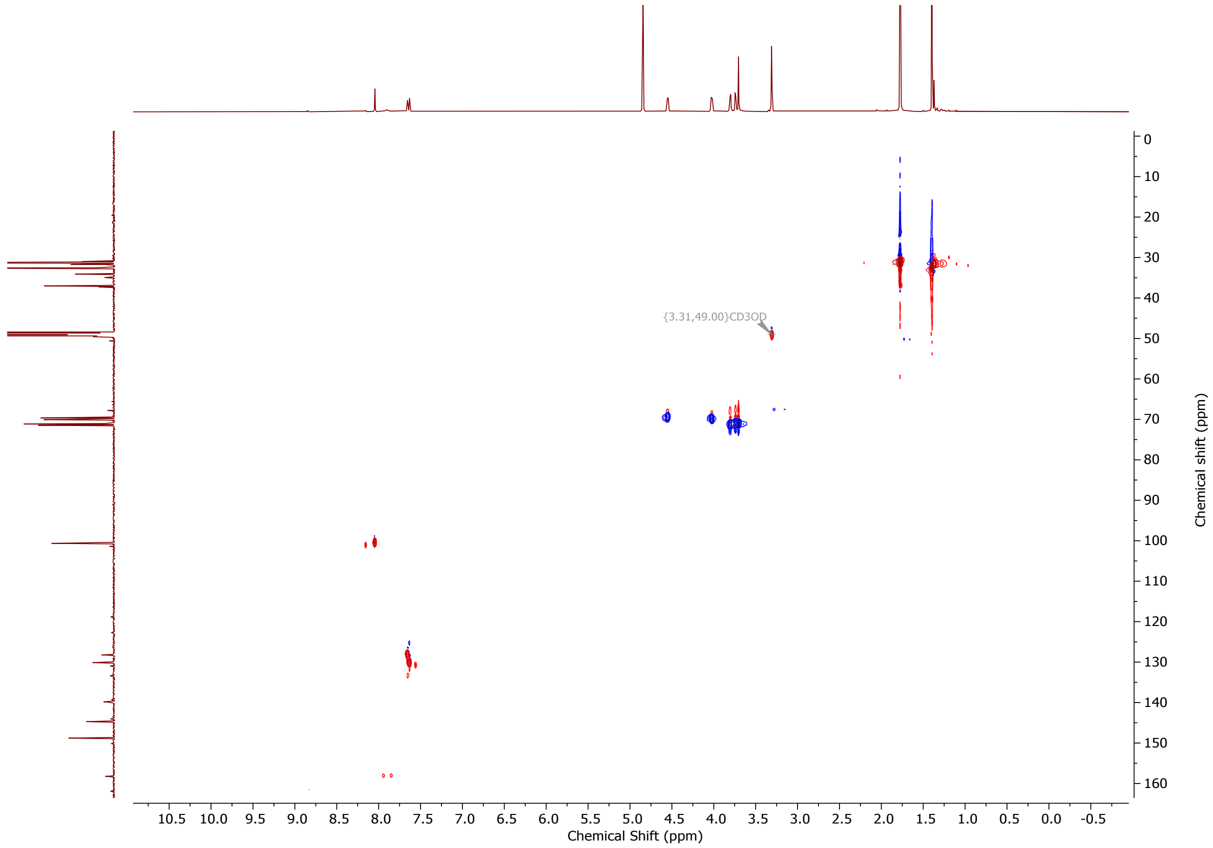

Figure S12. HSQC NMR spectrum of $L_{wide}CoK(OAc)_2$ in MeOD-d_4

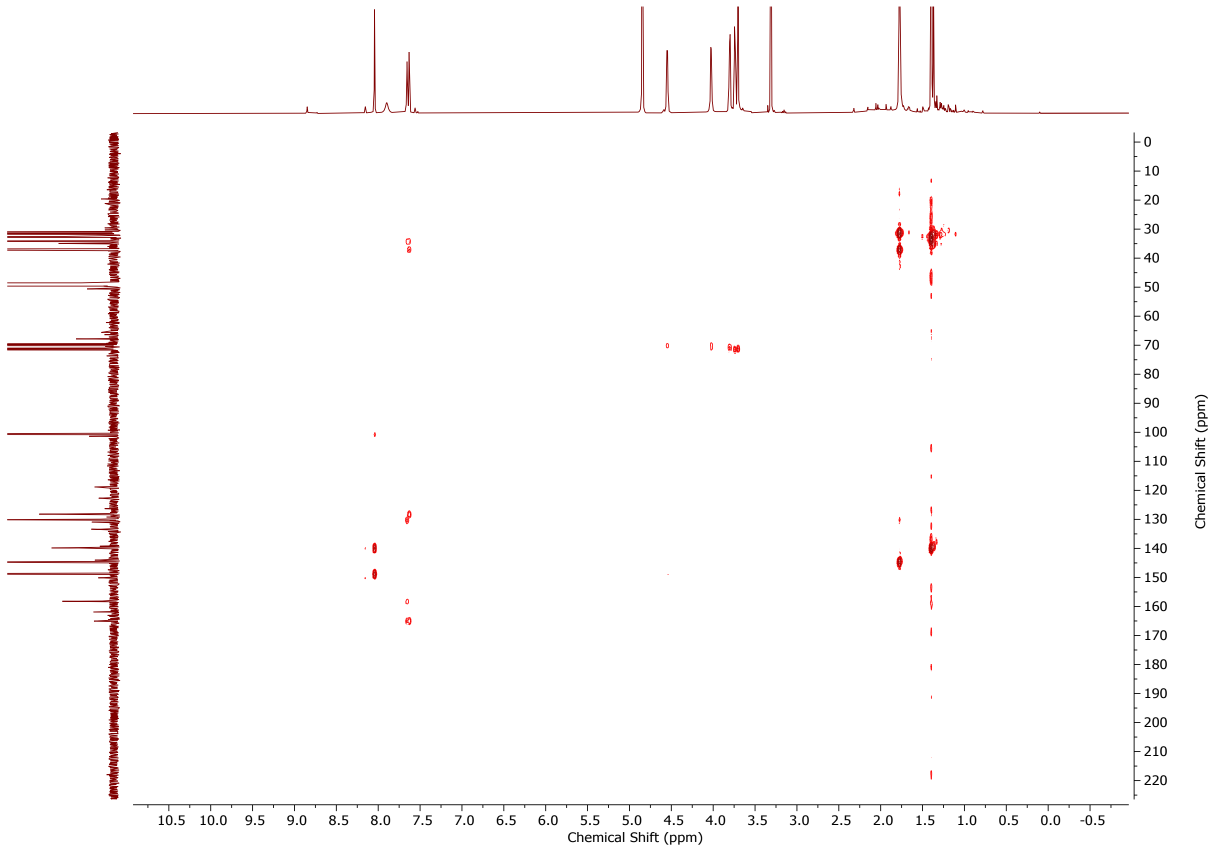

Figure S13. HMBC NMR spectrum of $L_{wide}CoK(OAc)_2$ in MeOD-d_4

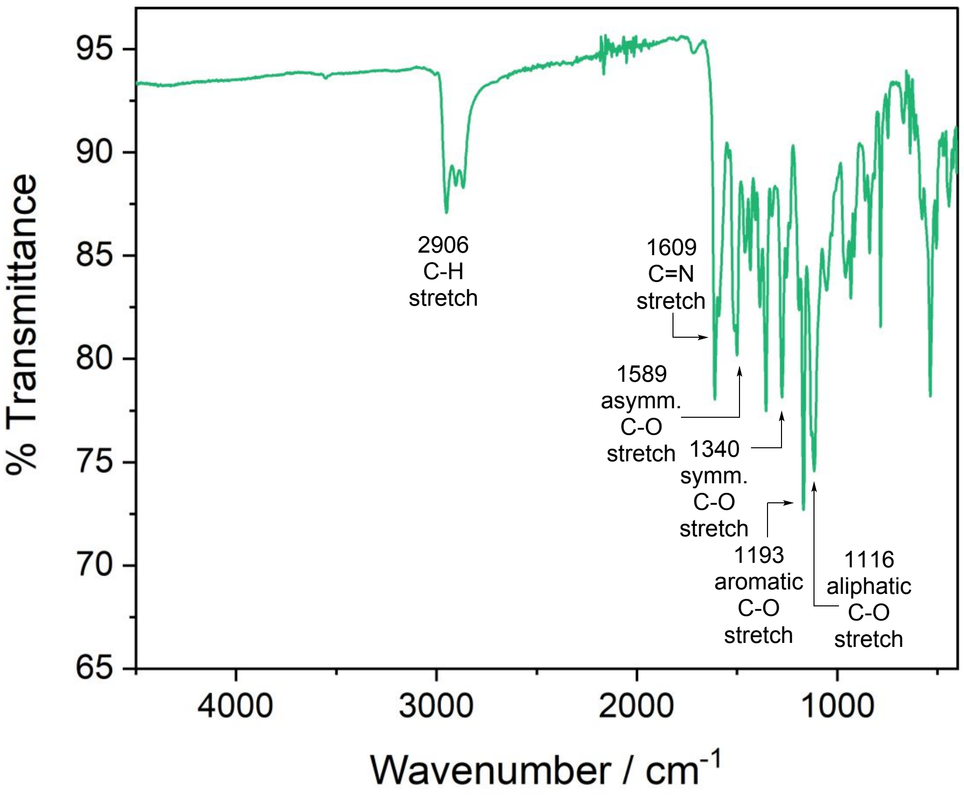

Figure S14. Solid state IR spectrum of $L_{wide}CoK(OAc)_2$.

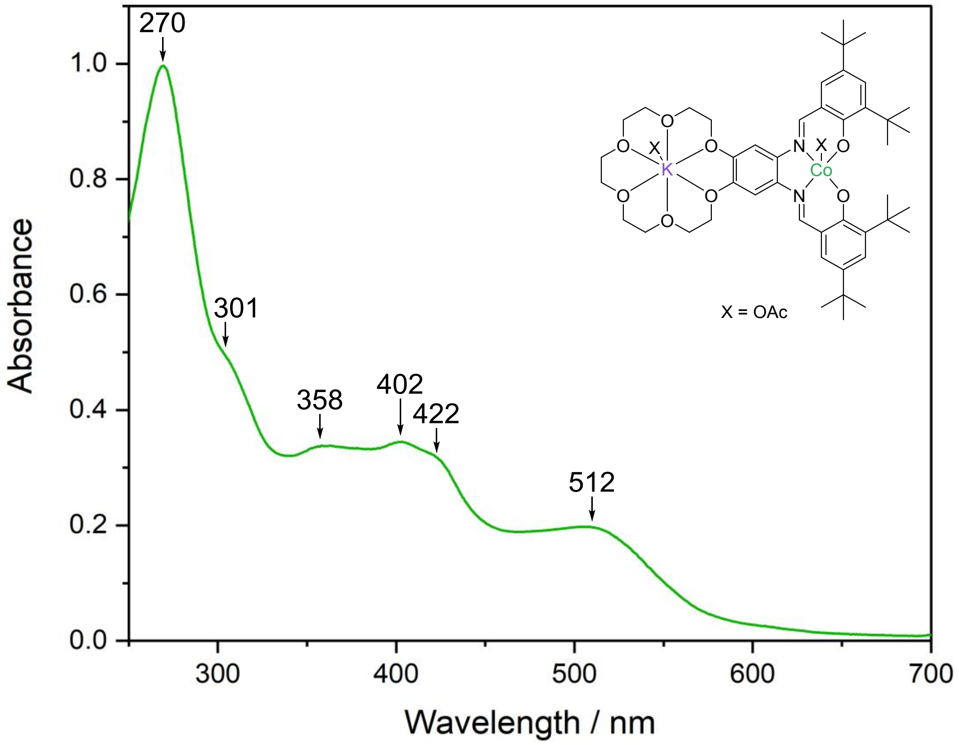

Figure S15. UV-vis spectrum of $L_{wide}CoK(OAc)_2$ in DCM.

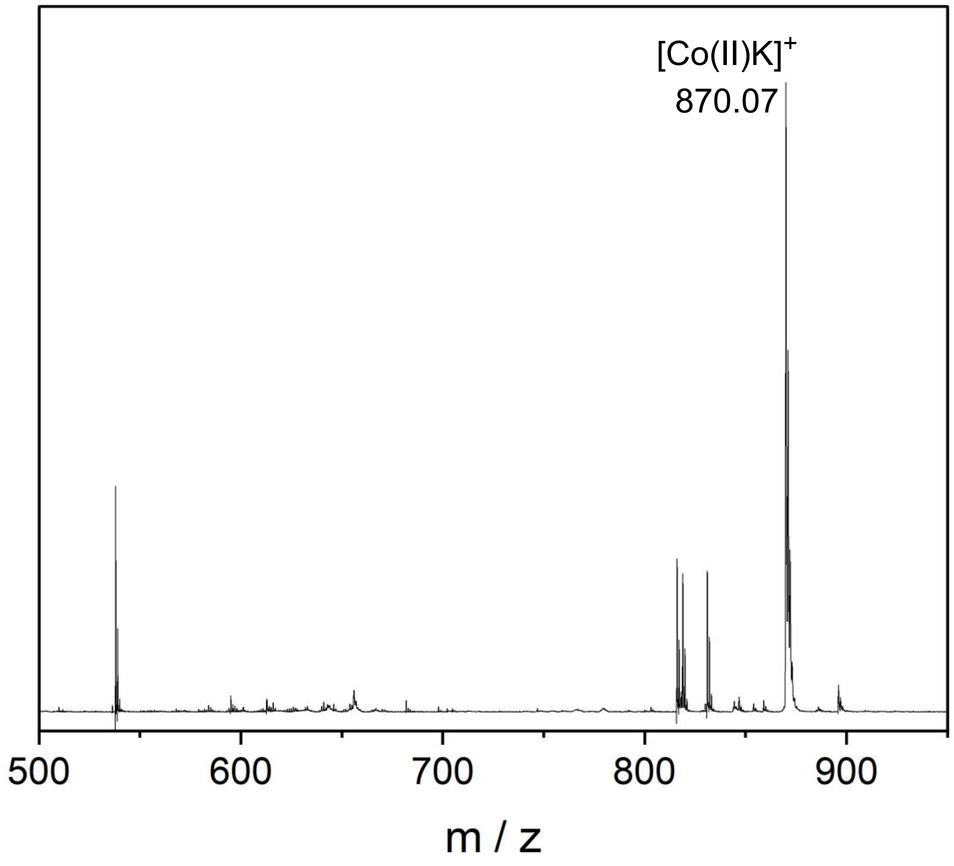

Figure S16. MALDI-TOF mass spectrometry trace of $L_{wide}CoK(OAc)_2$

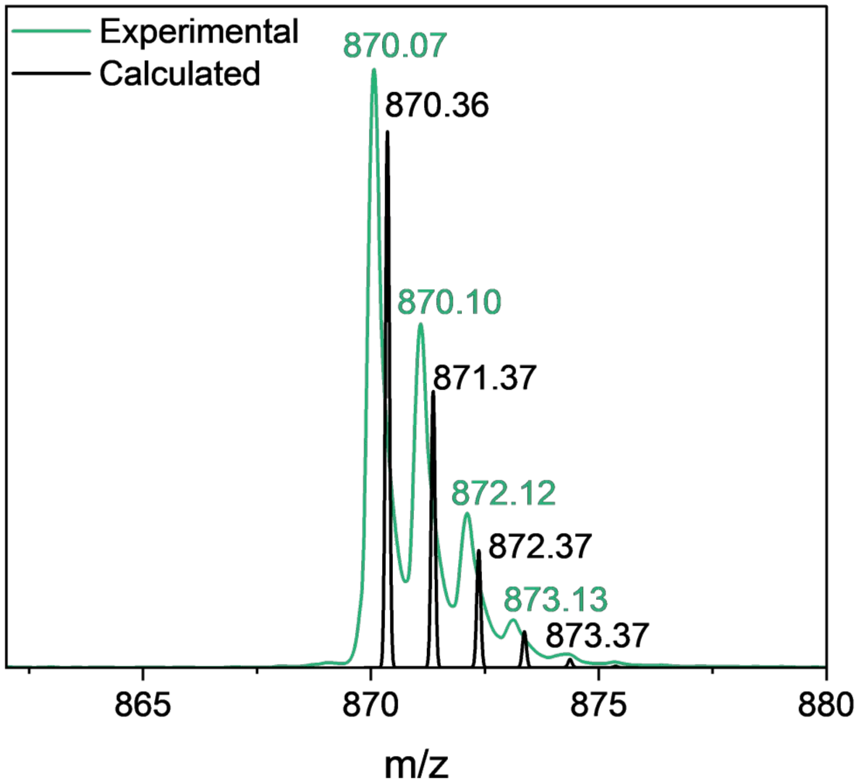

Figure S17. Experimental (green) and calculated (black) isotopic distributions of the $[Co(II)K(I)]^+$ peak

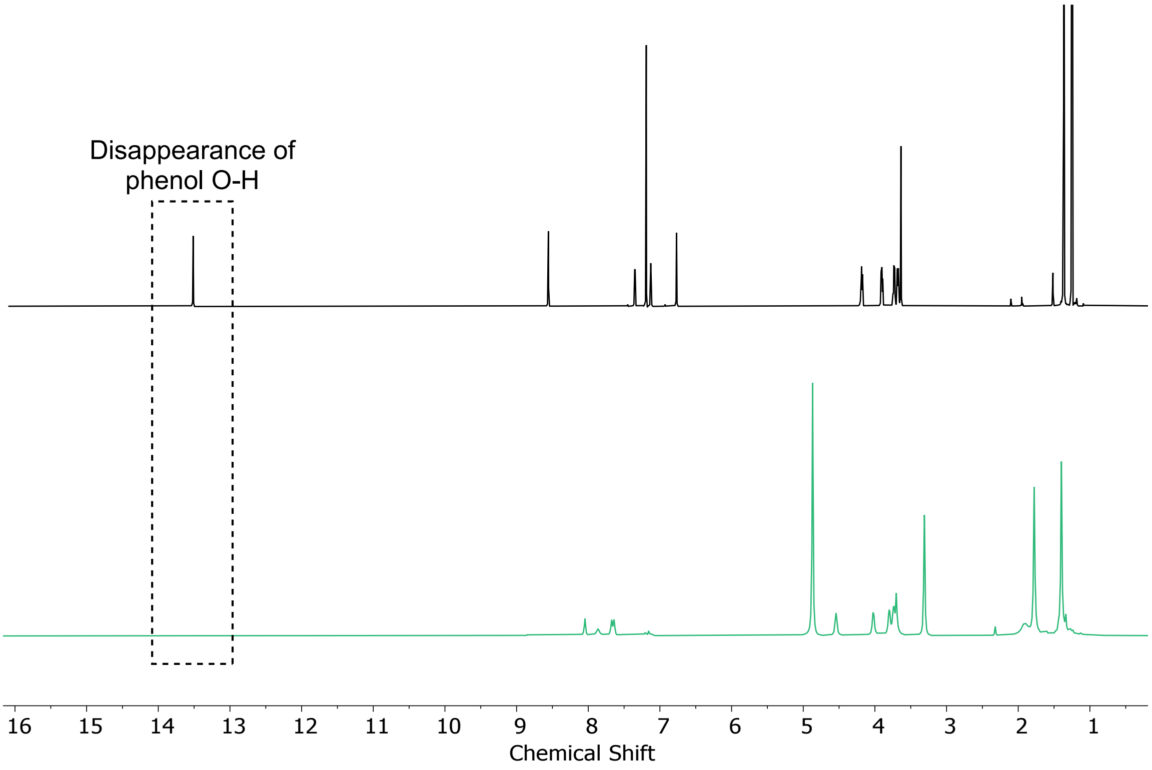

Figure S18. Stacked ^1H NMR spectra of H_2L_{wide} (top; black) in $CDCl_3$ and $L_{wide}CoK(OAc)_2$ (bottom; green) in MeOD-d_4 showing the disappearance of the phenol peak (dotted box).

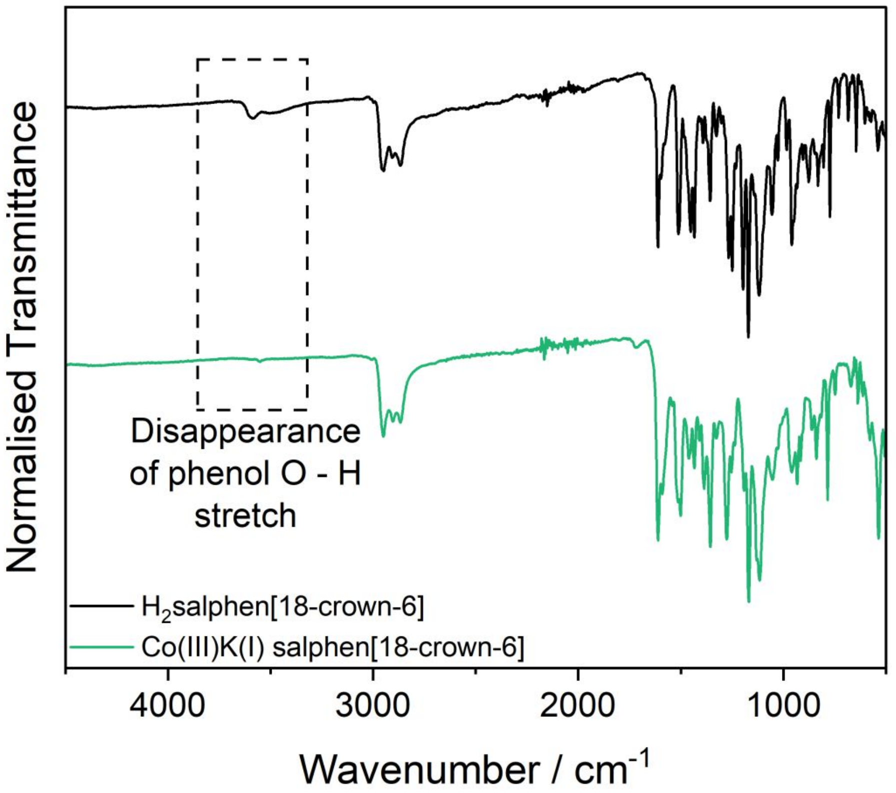

Figure S19. Overlaid IR spectra of H_2L_{wide} (back) and $L_{wide}CoK(OAc)_2$ (green) showing the disappearance of the phenol O-H stretch upon Co(III) coordination to the pro-ligand.

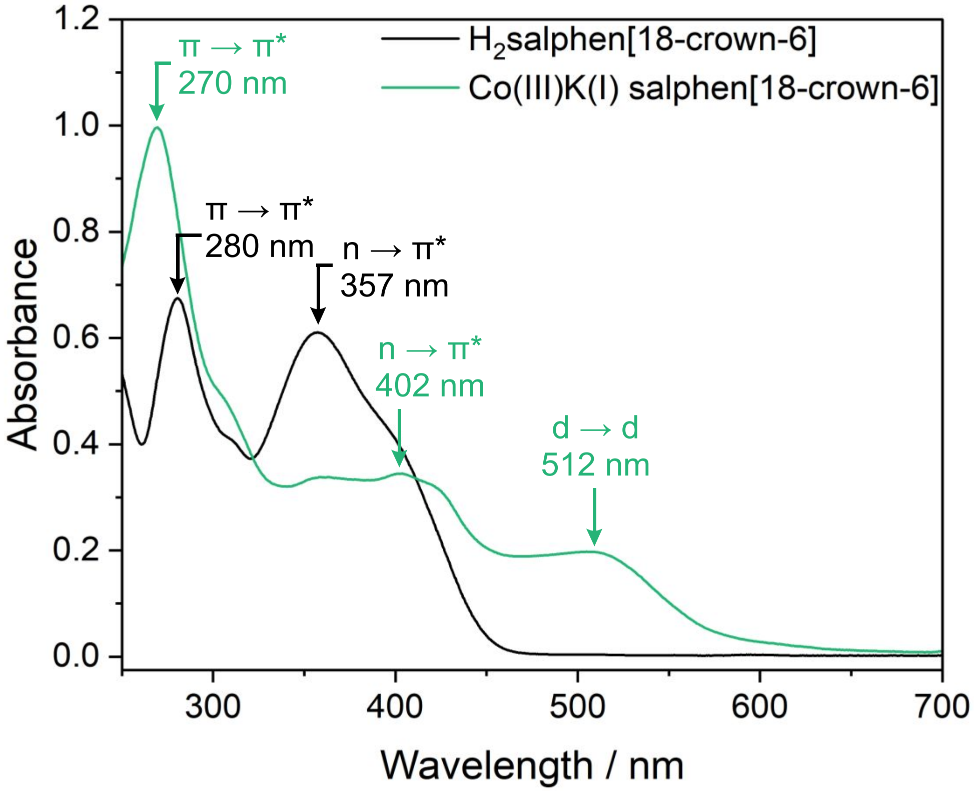

Figure S20. Overlaid UV-vis spectra of H_2L_{wide} (back) and $L_{wide}CoK(OAc)_2$ (green) showing shifting of $\pi \rightarrow \pi^*$, $n \rightarrow \pi^*$, and $d \rightarrow d$ transitions.

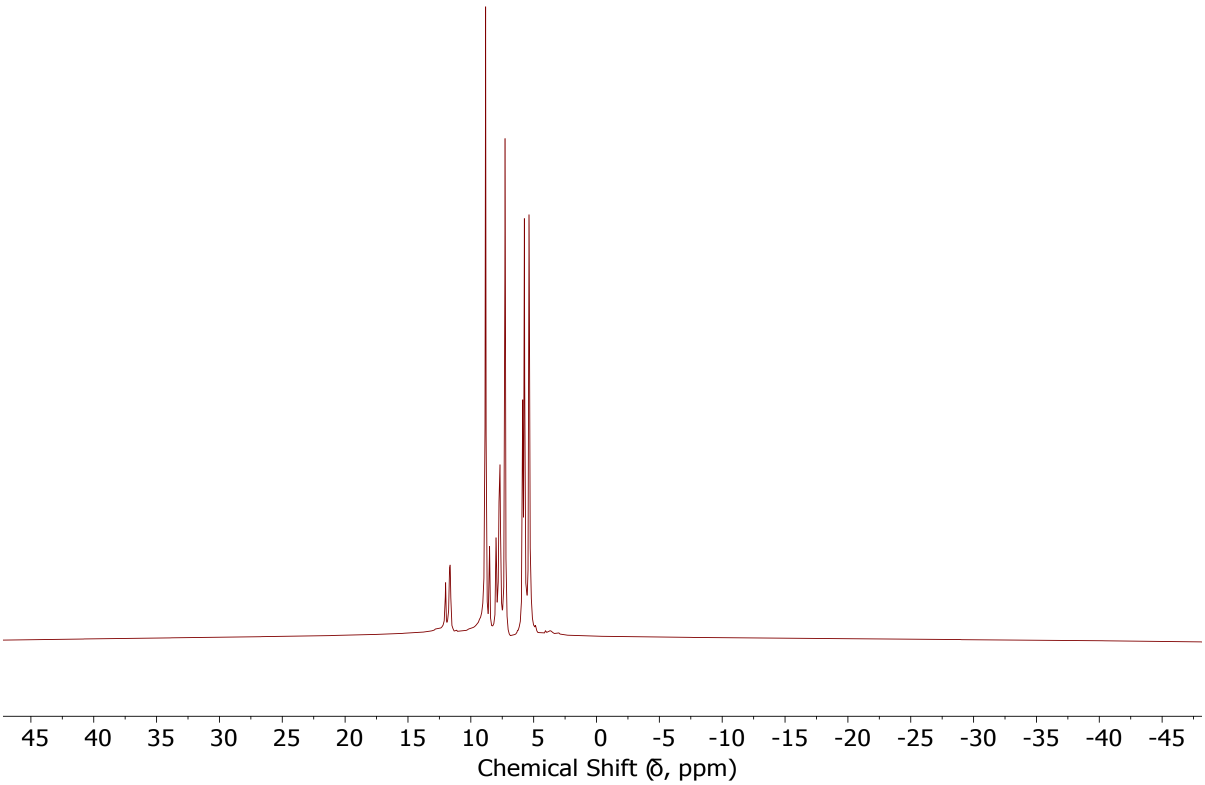

Figure S21. ^{1}H NMR spectrum of $L_{wide}CoK(OAc)_2$ in MeOD-d_4 between 50 to -50 ppm showing the absence of peaks outside of the typical range.

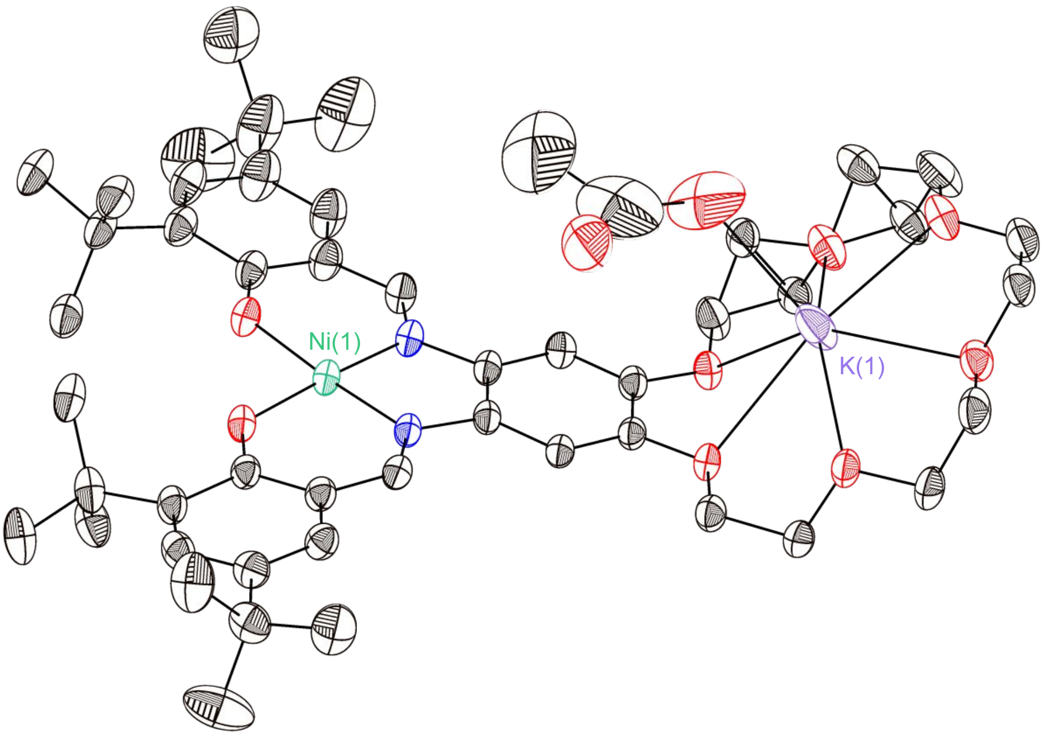

Figure S22. Half-dimer molecular structure of $L_{wide}NiKOAc$ determined by single crystals diffractometry. Solvent molecules and hydrogen atoms omitted for clarity, ellipsoids drawn at 40% probability.

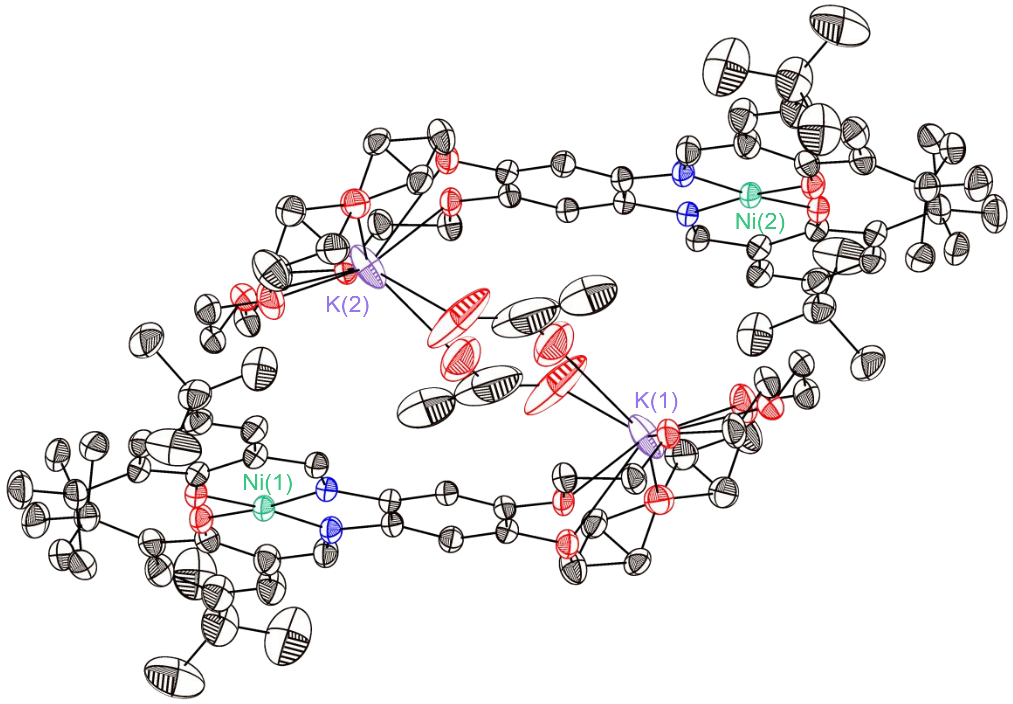

Figure S23. Dimeric molecular structure of L_{wide}NiKOAc determined by single crystals diffractometry. Solvent molecules and hydrogen atoms omitted for clarity, ellipsoids drawn at 40% probability.

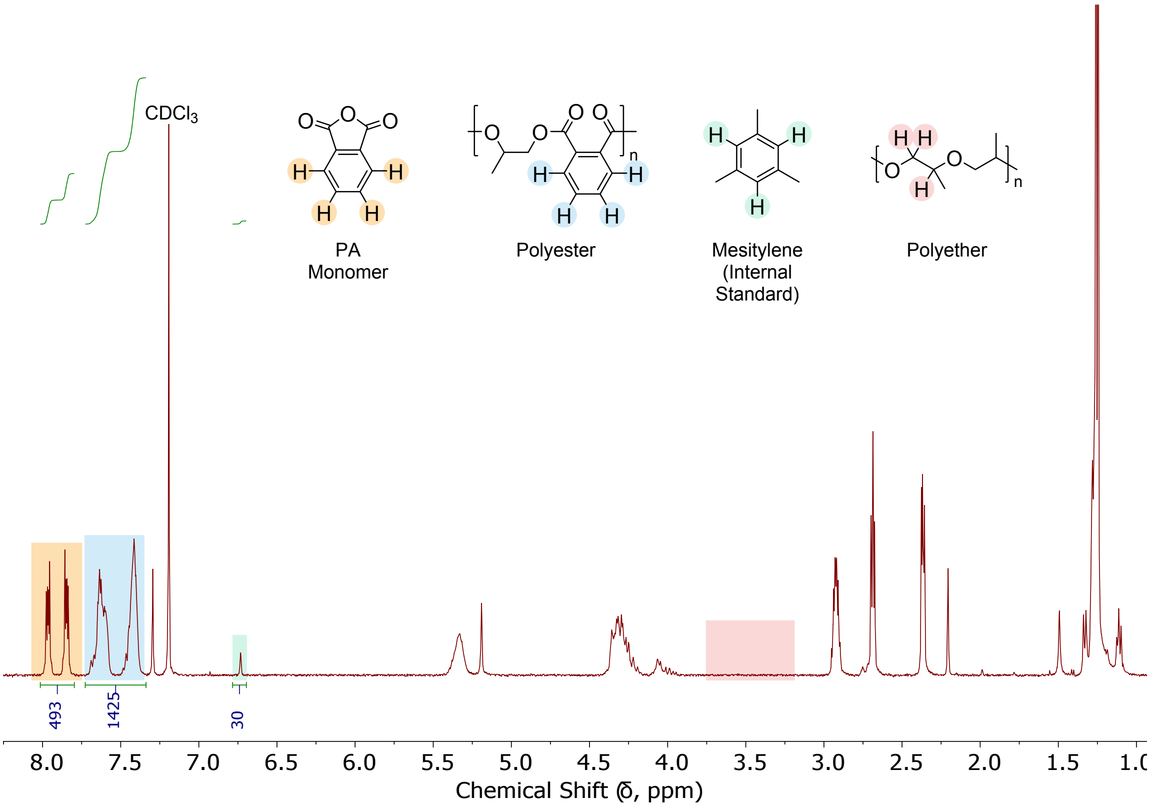

Figure S24. ^{1}H NMR spectrum of a representative PO/PA ROCOP reaction showing integrals and/or regions for PA monomer, polyester, mesitylene (10 eq. used as an internal standard), and polyether for the calculation of conversion, which is in turn used to calculate the turnover number (TON) and turnover frequency (TOF), and selectivity for polyester.

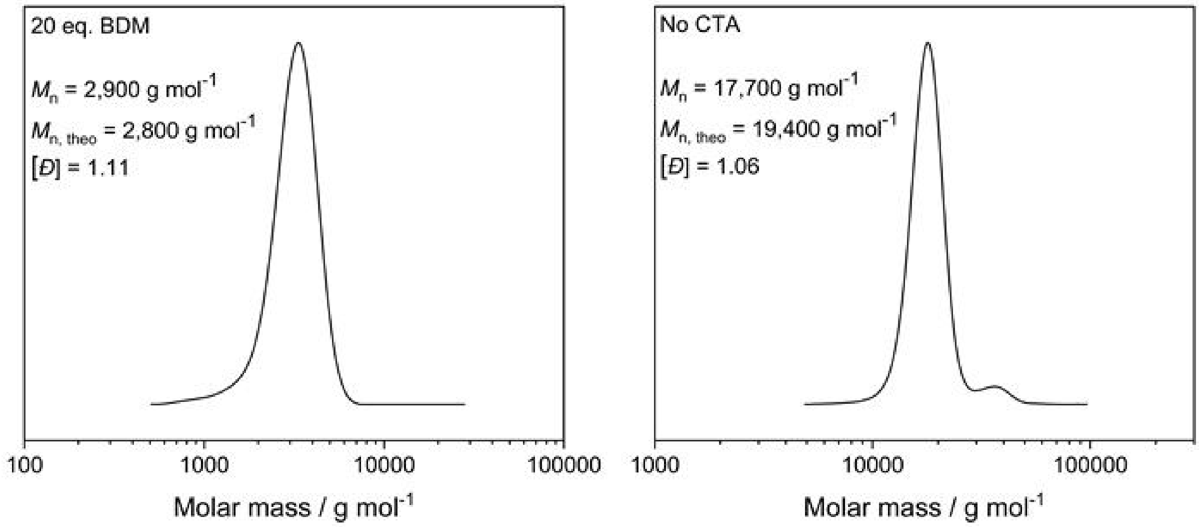

Figure S25. Representative GPCs of polyester produced from PA/PO ROCOP catalysed by $L_{wide}CoK(OAc)_2$ with and without added chain transfer agent.

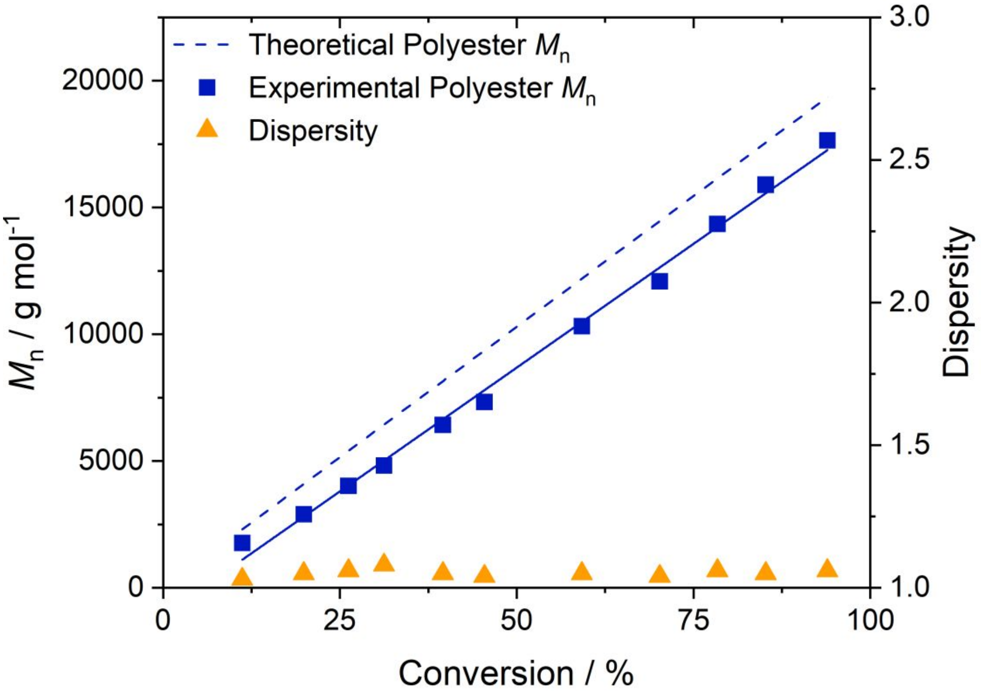

Figure S26. Representative plot of polyester M_n against conversion for PA/PO ROCOP catalysed by $L_{wide}CoK(OAc)_2$.

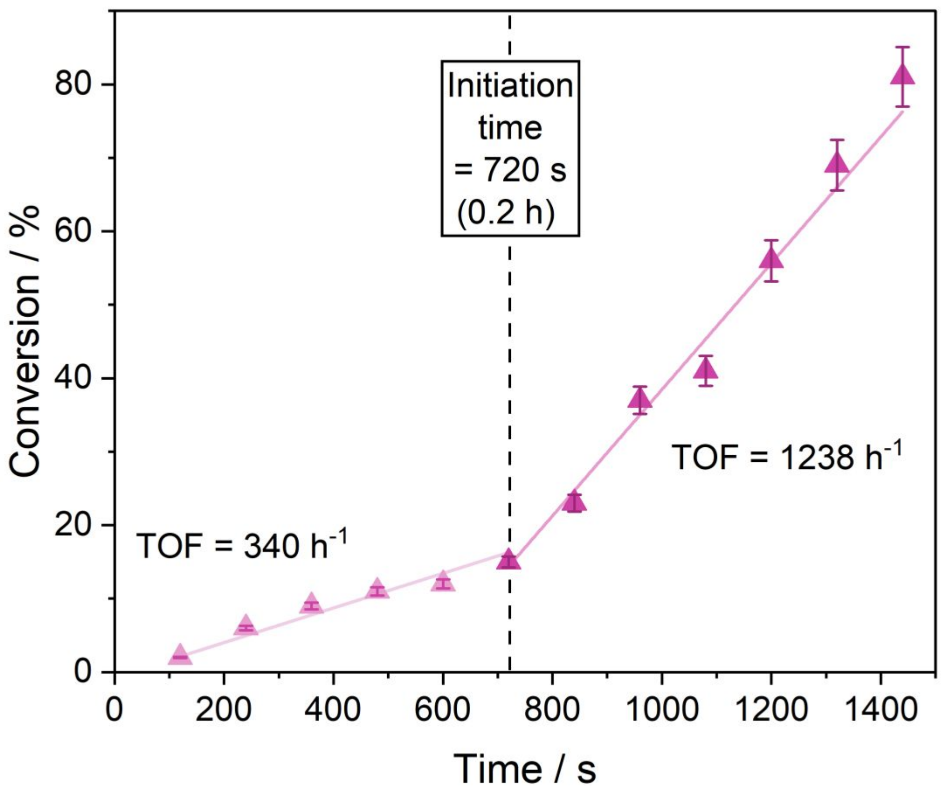

Figure S27. Conversion vs. time plot for PO/PA ROCOP catalysed by bicomponent system $L_{mono}CoOAc$ + KOAc[18-crown-6]. There are two regions to the plot; one with a slow, initial rate, and the other with a faster rate. The initiation time is taken as the time from which the faster rate occurs.

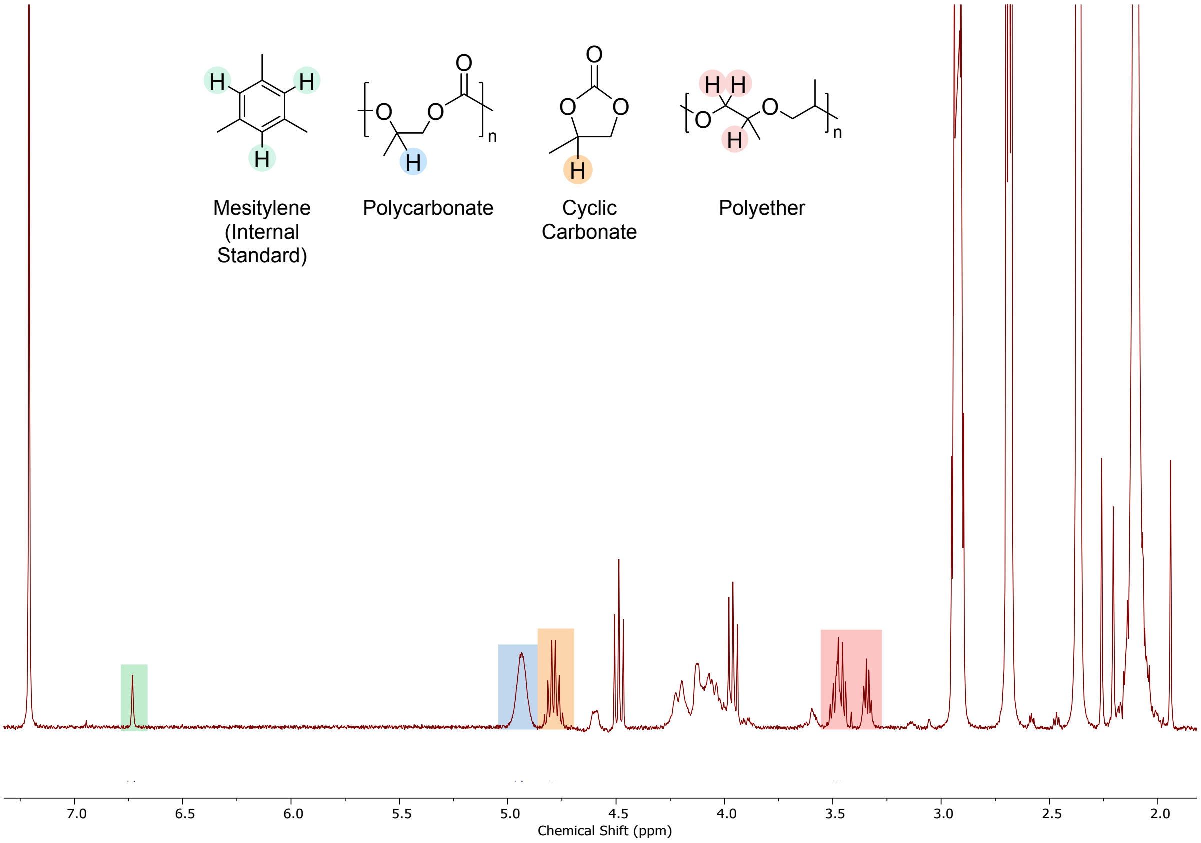

Figure S28. ^{1}H NMR spectrum of a representative PO/CO_2 ROCOP reaction showing integrals for mesitylene (10 eq. used as an internal standard), polycarbonate, cyclic carbonate, and polyether for the calculation of conversion, which is in turn used to calculate the turnover number (TON) and turnover frequency (TOF), and selectivity for CO_2 and polymer.

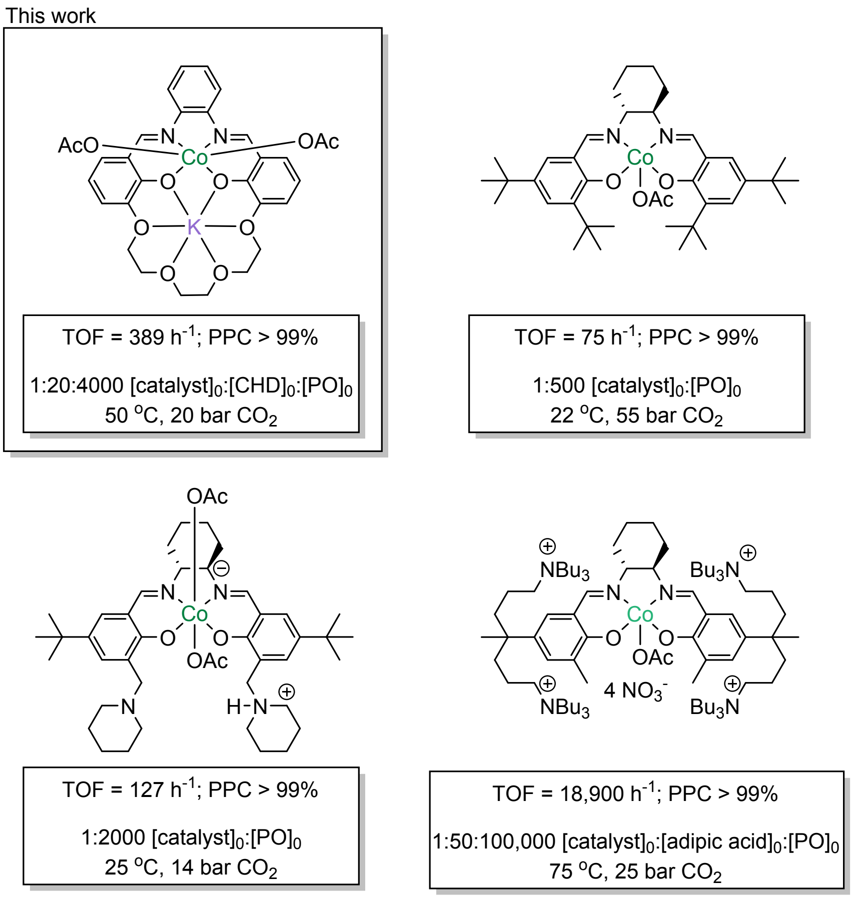

Figure S29. Structures, activites, selectivities and conditions for selected PO/CO_2 ROCOP catalysts.[14-17]

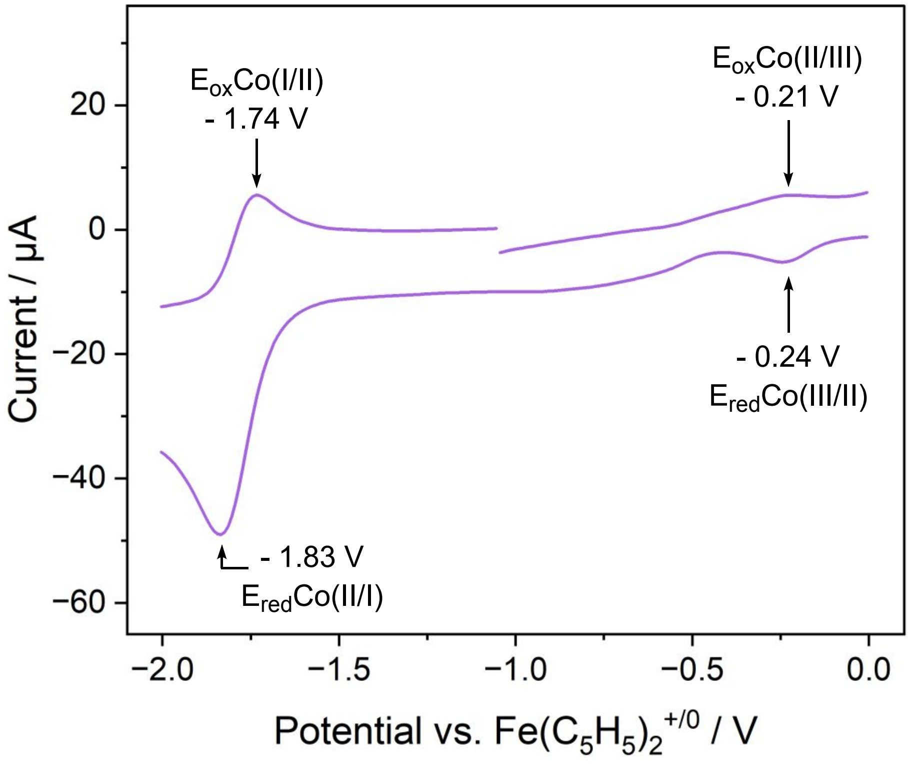

Figure S30. Cyclic voltammetry trace of $L_{wide}CoK(OAc)_2$.

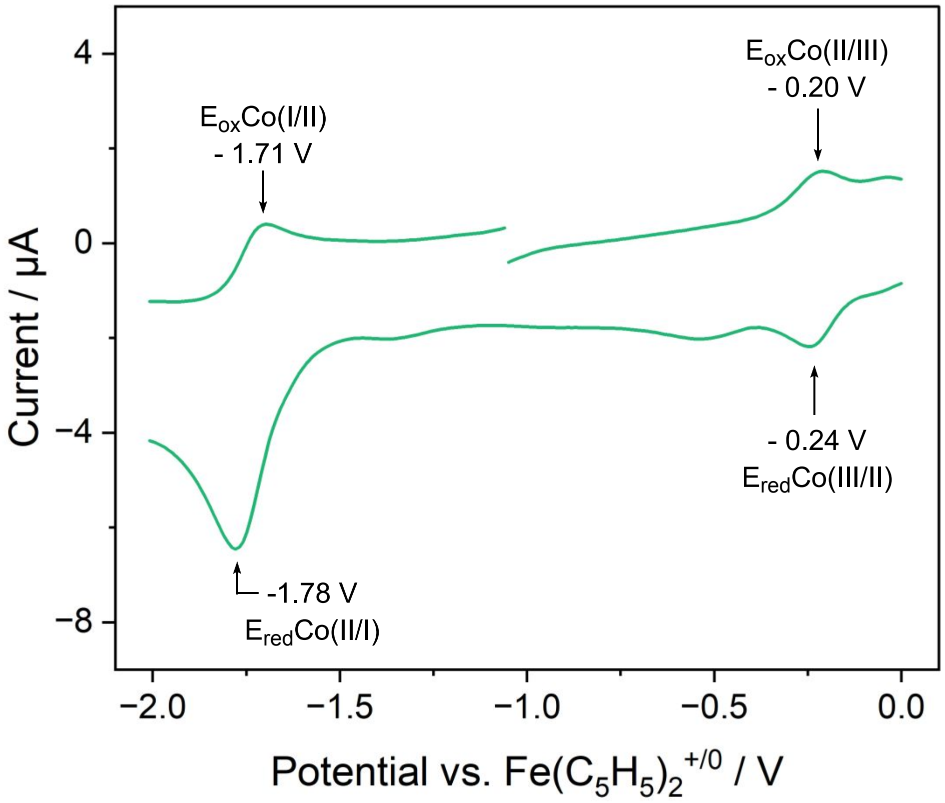

Figure S31. Cyclic voltammetry trace of L_{mono}CoOAc.

Table 3. Summary of Co(III/II) redox potentials and ROCOP activities/selectivities for $L_{wide}CoK(OAc)_2$, the bicomponent system L_{mono}CoOAc + KOAc[18-crown-6], $L_{short}CoK(OAc)_2$, and an analogue of $L_{short}CoK(OAc)_2$ with a chlorinated phenyl amine backbone ($L_{short(Cl)}CoK(OAc)_2$)

Catalyst	$E_{1/2}$ Co(II/III) / V	PO/PA ROCOP	PO/CO_2 ROCOP	
		TOF / h^{-1}	TOF / h^{-1}	PPC / %
$L_{wide}CoK(OAc)_2$	- 0.23	1686	0	0
L_{mono}CoOAc + KOAc[18-crown-6]	- 0.22	1238	231*	82*
$L_{short}CoK(OAc)_2$	- 0.41	231	389	> 99
$L_{short(Cl)}CoK(OAc)_2$	- 0.25	N.D.	62	75

Reaction conditions: PO/PA ROCOP: 1:20:400:1000 $[catalyst]_0:[BDM]_0:[PA]_0:[PO]_0$, 60 °C; PO/CO_2 ROCOP: 1:20:4000 $[catalyst]_0:[CHD]_0:[PO]_0$, 50 °C, 20 bar CO_2 pressure.

* PO/CO_2 ROCOP catalysed by L_{mono}CoOAc + KOAc[18-crown-6] performed under the following conditions: 1:20:1000 $[catalyst]_0:[CHD]_0:[PO]_0$, 50 °C, 20 bar CO_2 pressure.

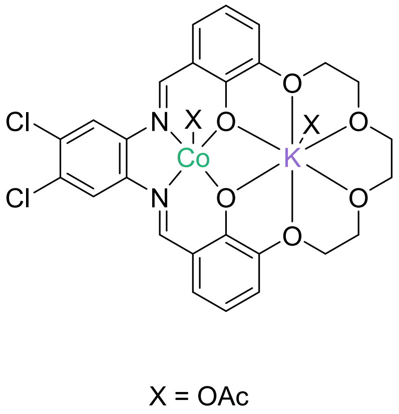

Figure S32. Structure of the chlorinated analogue of $L_{short}CoK(OAc)_2$.

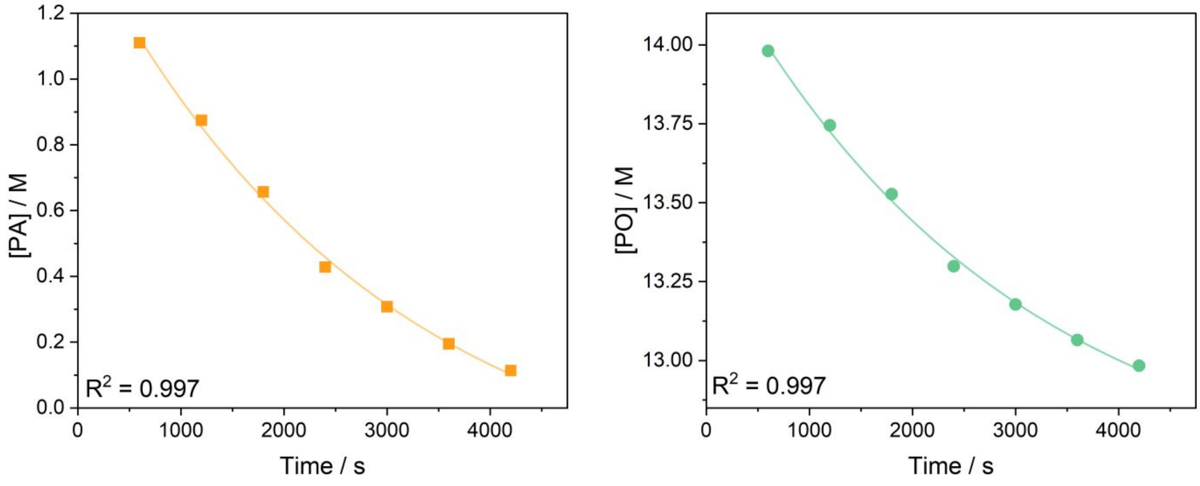

Figure S33. Representative plots of the concentration of PA (LHS) and PO (RHS) over the course of a polymerization (1:100:1000 $[L_{wide}CoK(OAc)_2]_0$:$[PA]_0$:$[PO]_0$ with exponential fits. Indicates first-order dependence on [PA] and pseudo-zero order dependence on [PO] due to the large excess of PO.

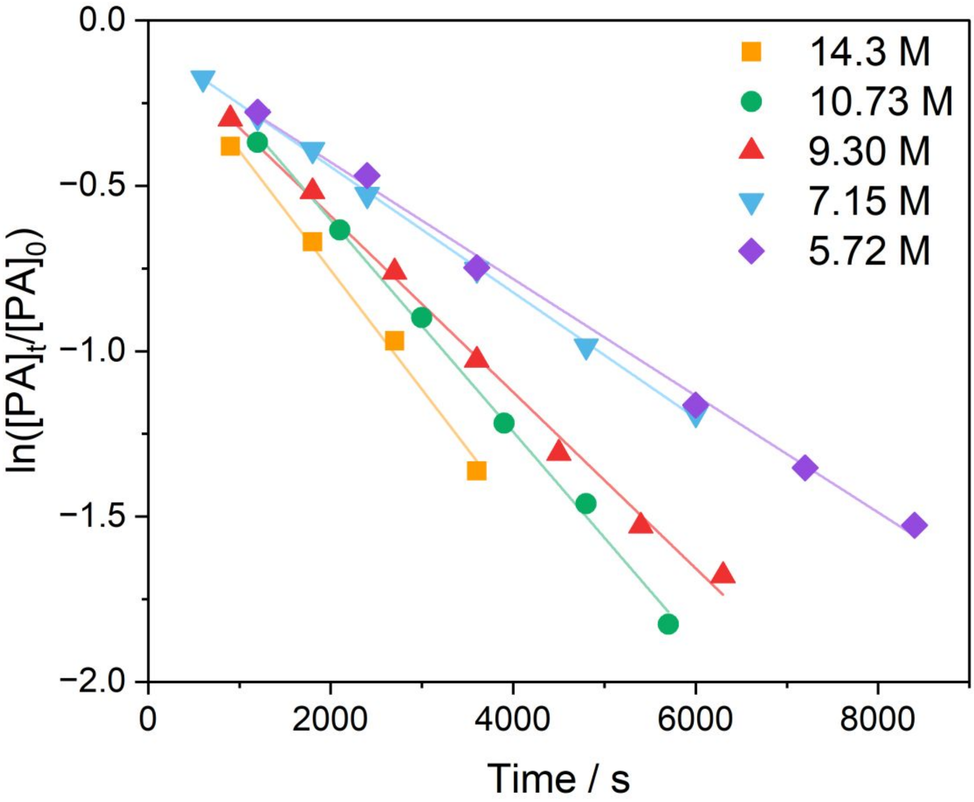

Figure S34. Plots of $\ln([PA]_t/[PA]_0)$ for varying concentrations of PO showing the increase in rate with increasing PO concentration.

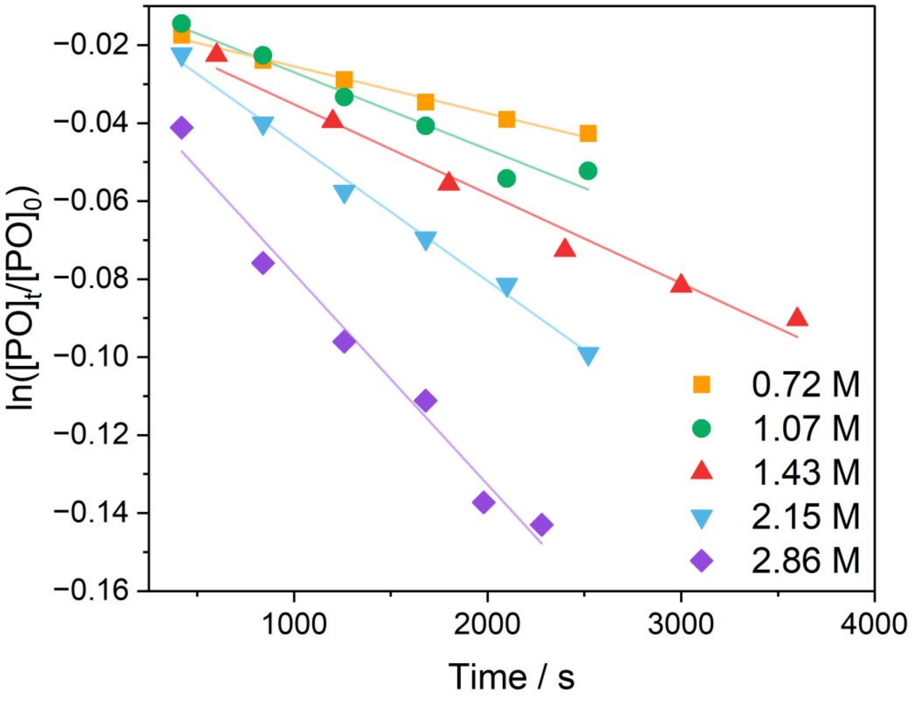

Figure S35. Plots of $\ln([PO]_t/[PO]_0)$ for varying concentrations of PA showing the increase in rate with increasing PA concentration.

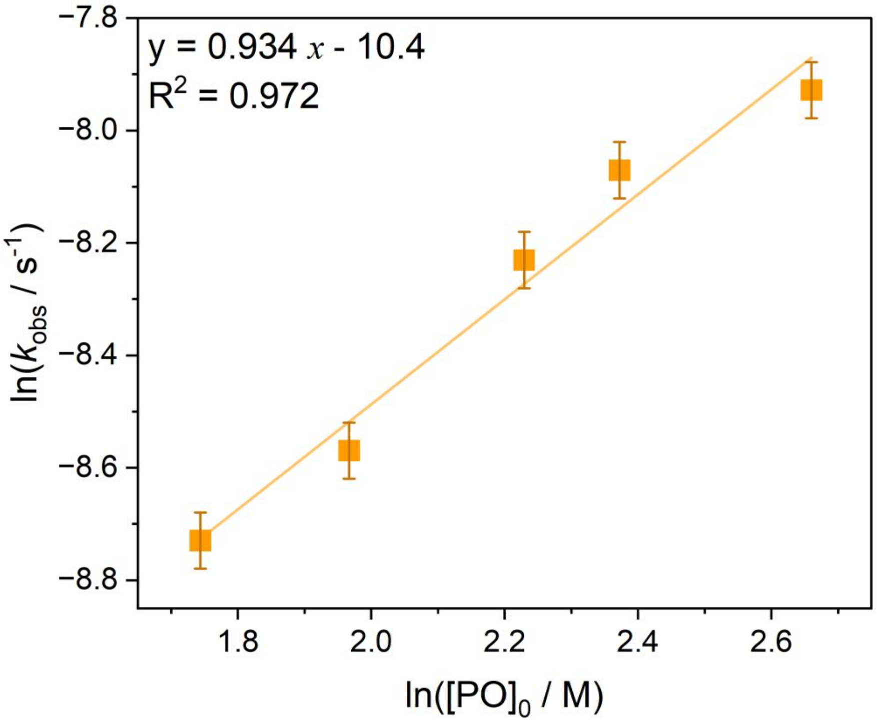

Figure S36. Plot of k_{obs} vs. $[PO]_0$ showing a linear dependence.

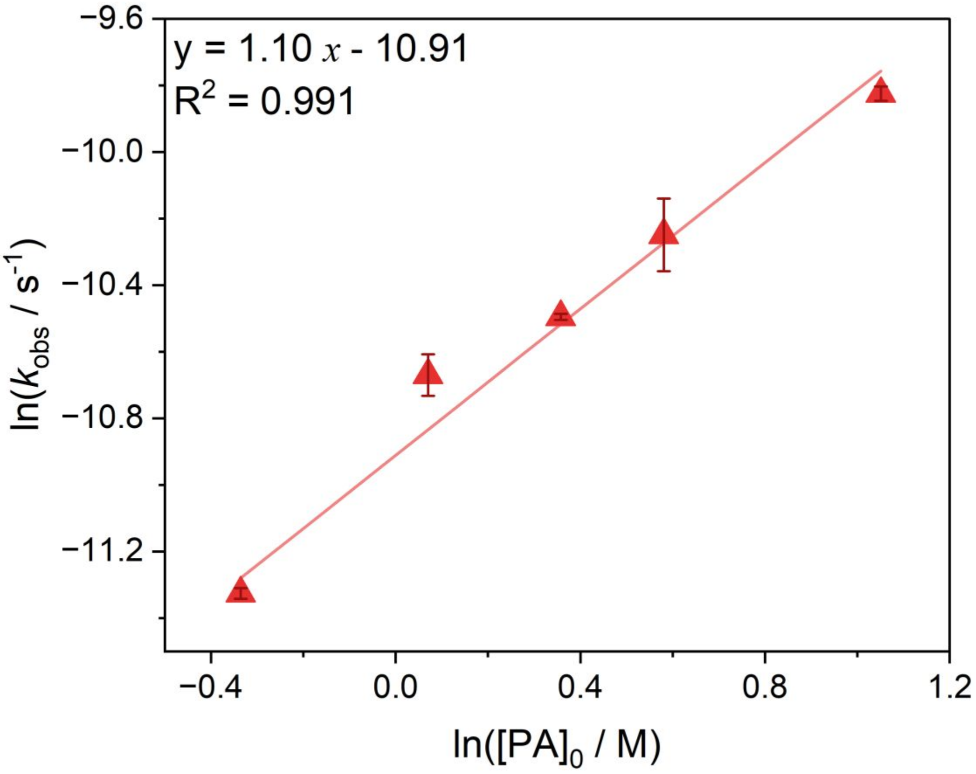

Figure S37. Plot of k_{obs} vs. $[PA]_0$ showing a linear dependence.

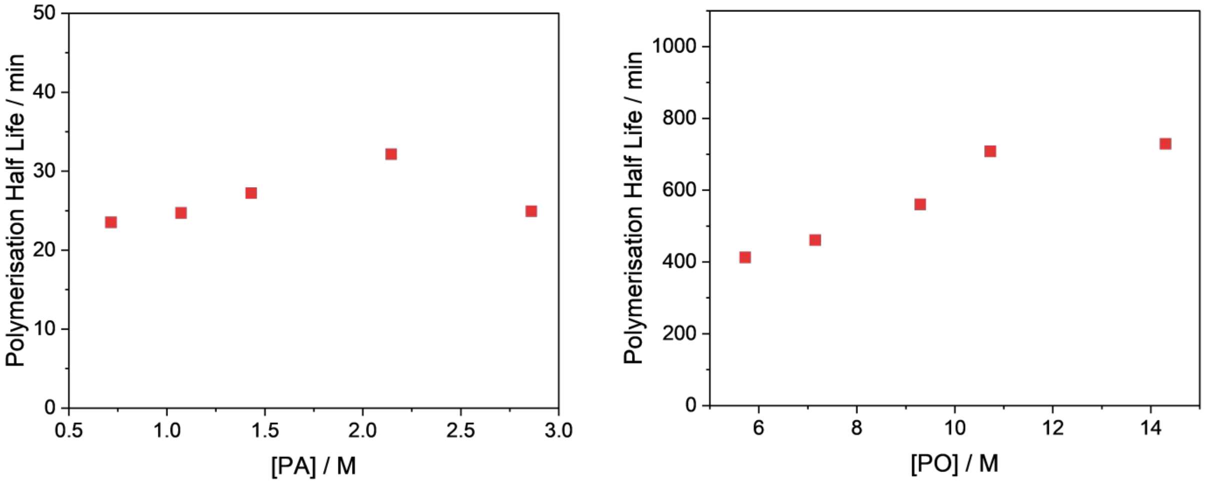

Figure S38. Graphs showing consistent polymerisation half-life with changing concentrations of PA and PO, supporting first-order dependencies of rate on the concentration of each monomer.

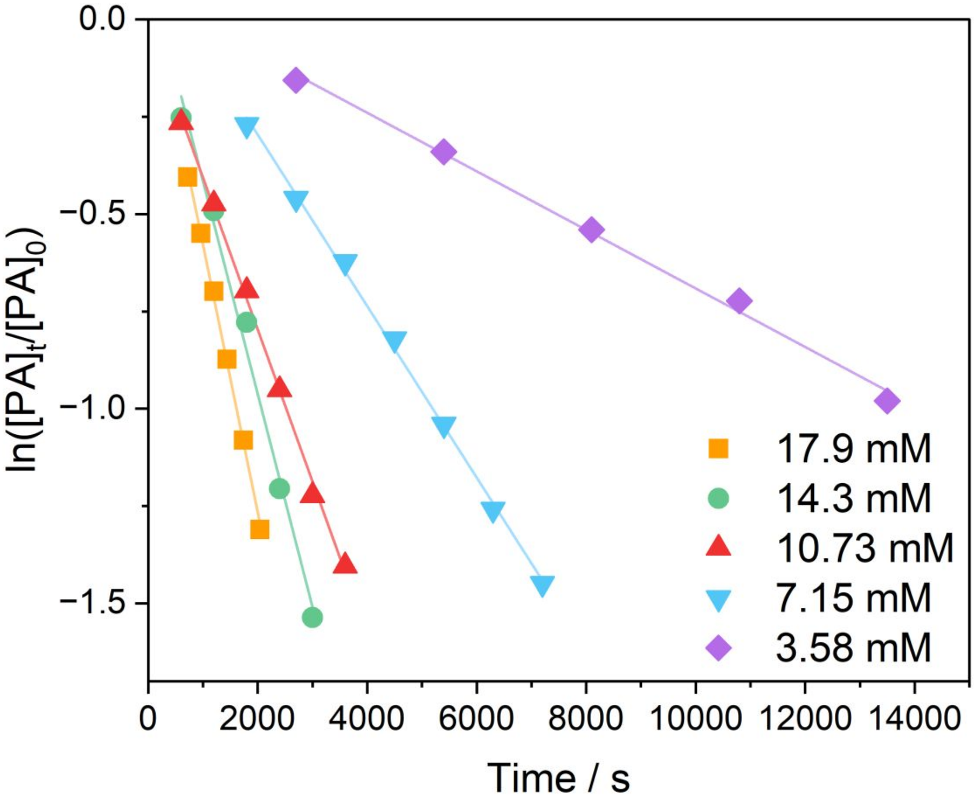

Figure S39. Plots of $\ln([PA]_t/[PA]_0)$ for varying concentrations of $L_{wide}CoK(OAc)_2$ showing the increase in rate with increasing $L_{wide}CoK(OAc)_2$ concentration.

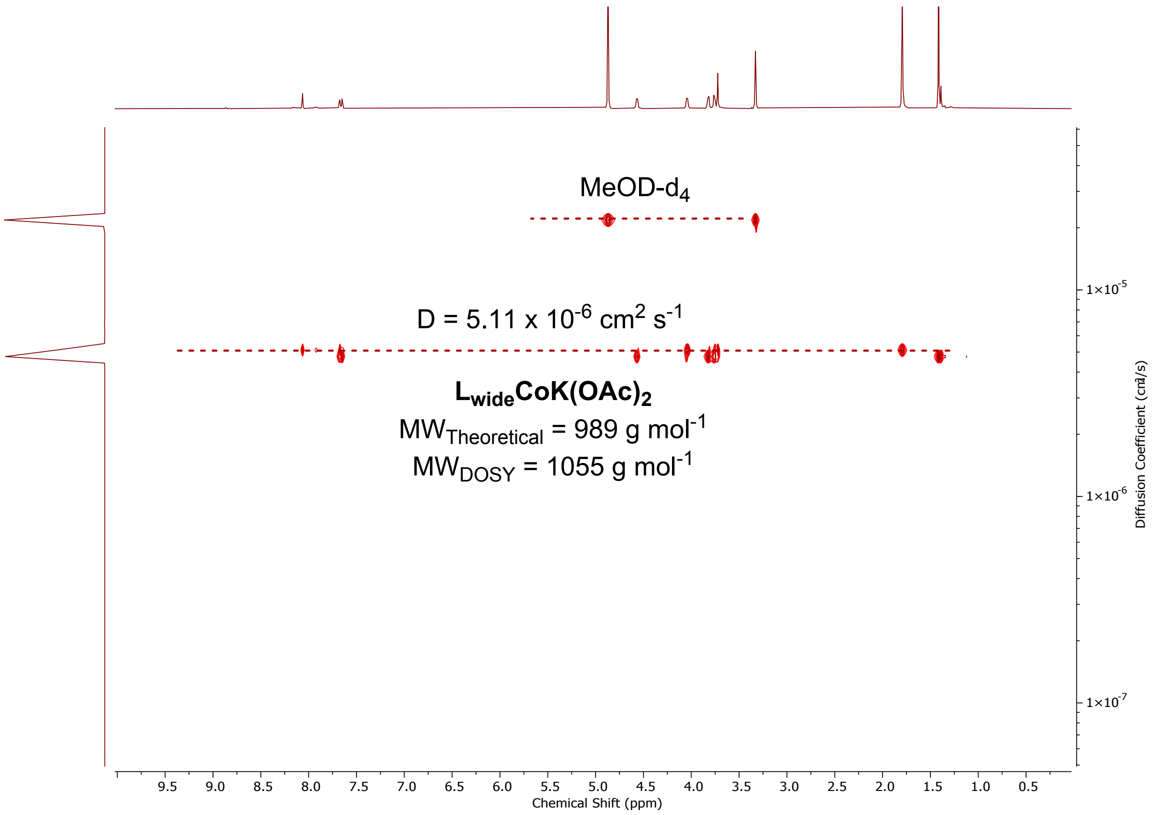

Figure S40. DOSY NMR spectrum of $L_{wide}CoK(OAc)_2$ in MeOD-d_4.

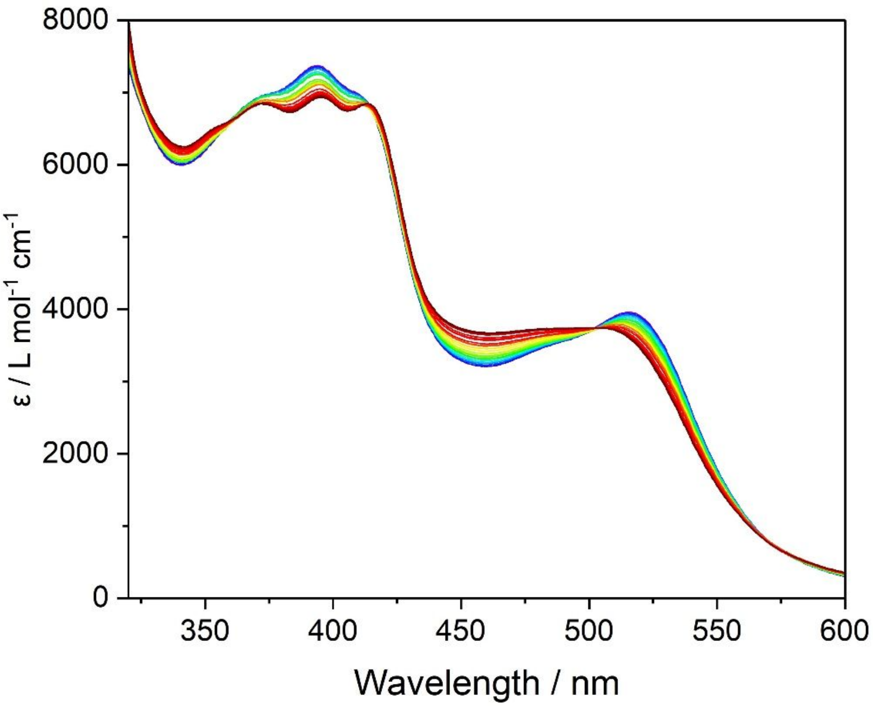

Figure S41. Stacked UV-vis spectra of $L_{wide}CoK(OAc)_2$ with increasing concentration of PA (from 0 (red) – 250,000 (blue) equivalents). Initial concentration of $L_{wide}CoK(OAc)_2$ = 0.175 μM in THF.

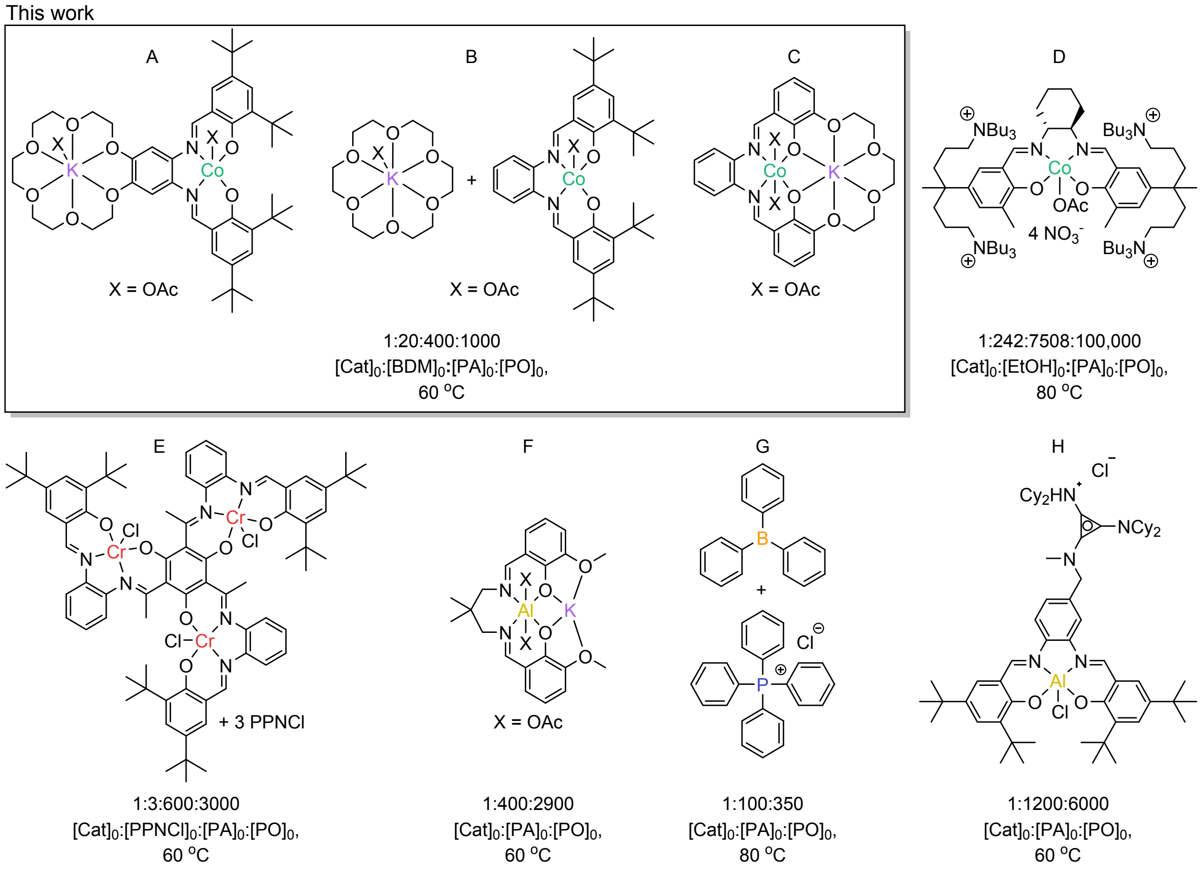

Figure S42. Literature catalysts for PO/PA ROCOP used for comparison.[6, 10, 18, 19]

The vials were tested to ensure that they were able to contain the pressure associated with PO heated to well above its boiling point of 34 °C, by weighing the vial containing the polymerization solution before and after each period of heating. No change in weight was observed, indicating that no PO was evaporating from the vial over the course of the polymerization (Table S5). Before removing each aliquot, the polymerization was cooled in an acetone/dry ice bath to ensure that no evaporation of PO occurred during the aliquoting process.

Table S4. Table of weights of the vial containing the polymerization solution of PO/GA at 70 °C demonstrating that no PO is evaporating from the vial during heating of the polymerization mixture.

Time heated / min	Weight before aliquot / g	Weight after aliquot / g
0	7.742	-
3	7.743	7.733
6	7.733	7.727
9	7.727	7.716
15	7.717	7.692

Crystallographic Data for L_{wide}NiKOAc

Table S5. Selected bond lengths and angles for L_{wide}NiKOAc.

Bond	Length	Bond	Angle
**Ni1—O8**	1.8500 (16)	**K1—O5**	2.813 (3)
**Ni1—O7**	1.8464 (17)	**K1—O6**	2.768 (2)
**Ni1—N1**	1.8551 (19)	**K1—O9**	2.750 (5)
**Ni1—N2**	1.853 (2)	**N1—C17**	1.299 (3)
**K1—O3**	2.8333 (18)	**N2—C32**	1.295 (3)
**K1—O4**	2.8651 (19)	**O10—C47**	1.356 (11)
**K1—O2**	2.832 (2)	**O9—C47**	1.410 (13)
**K1—O1**	2.849 (2)	**N2—Ni1—N1**	86.19 (8)
**O8—Ni1—N1**	174.46 (8)	**O1—K1—O4**	140.81 (7)
**O8—Ni1—N2**	94.38 (8)	**O5—K1—O2**	149.14 (7)
**O7—Ni1—O8**	85.25 (8)	**O6—K1—O3**	165.01 (8)
**O7—Ni1—N1**	94.67 (8)		
**O7—Ni1—N2**	174.94 (8)		

Table S6. Summary of crystallographic refinement data for L_{wide}NiKOAc.

Complex	L_{wide}NiKOAc
**Local Code**	005ff23_autored-ff
**CCDC Deposition Number**	2342581
**Crystal data**	
**Chemical formula**	$C_{48}H_{67}KN_2NiO_{10}$
M_r	929.84
**Crystal system, space group**	Triclinic, *P*1
**Temperature (K)**	150
a, b, c **(Å)**	10.6691 (2), 17.0018 (3), 17.7820 (3)
α, β, γ **(°)**	94.563 (1), 102.757 (1), 104.684 (2)
V **(Å^3)**	3011.61 (10)
**Z**	2
**Radiation type**	Cu *K*α
**μ (mm^{-1})**	1.46
**Crystal size (mm)**	0.165 x 0.169 x 0.445
**Data Collection**	
**Diffractometer**	SuperNova, Dual, Cu at home/near, Atlas
**Absorption correction**	Multi-scan *CrysAlis PRO* 1.171.42.72a (Rigaku Oxford Diffraction, 2022) Empirical absorption correction using spherical harmonics, implemented in SCALE3 ABSPACK scaling algorithm.
T_{min}, T_{max}	0.402, 1.000
**No. of measured, independent and observed [$I > 2\sigma(I)$] reflections**	74081, 12546, 10375
R_{int}	0.052
**(sin θ/λ)max (Å−1)**	0.631
**Refinement**	
$R[F^2 > 2\sigma(F^2)]$, $wR(F^2)$, S	0.064, 0.184, 1.05
**No. of reflections**	12546
**No. of parameters**	593
**No. of restraints**	22
**H-atom treatment**	H-atom parameters constrained
$(\Delta/\sigma)_{max}$	0.776
$\Delta\rho_{max}$, $\Delta\rho_{min}$ (e Å^{-3})	0.94, −1.38

References

(1) Arnold, P. L.; Turner, Z. R.; Bellabarba, R.; Tooze, R. P. Carbon–Silicon and Carbon–Carbon Bond Formation by Elimination Reactions at Metal N-Heterocyclic Carbene Complexes. *J. Am. Chem. Soc.* **2011**, *133* (30), 11744-11756.

(2) Moore, D. R.; Cheng, M.; Lobkovsky, E. B.; Coates, G. W. Mechanism of the Alternating Copolymerization of Epoxides and CO2 Using β-Diiminate Zinc Catalysts: Evidence for a Bimetallic Epoxide Enchainment. *J. Am. Chem. Soc.* **2003**, *125* (39), 11911-11924.

(3) Lehenmeier, M. W.; Kissling, S.; Altenbuchner, P. T.; Bruckmeier, C.; Deglmann, P.; Brym, A.-K.; Rieger, B. Flexibly Tethered Dinuclear Zinc Complexes: A Solution to the Entropy Problem in CO2/Epoxide Copolymerization Catalysis? *Angew. Chem. Int. Ed.* **2013**, *52* (37), 9821-9826.

(4) Liu, Y.; Ren, W.-M.; Liu, J.; Lu, X.-B. Asymmetric Copolymerization of CO_2 with meso-Epoxides Mediated by Dinuclear Cobalt(III) Complexes: Unprecedented Enantioselectivity and Activity. *Angew. Chem. Int. Ed.* **2013**, *52* (44), 11594-11598.

(5) Bok, T.; Yun, H.; Lee, B. Y. Bimetallic Fluorine-Substituted Anilido−Aldimine Zinc Complexes for CO2/(Cyclohexene Oxide) Copolymerization. *Inorg. Chem.* **2006**, *45* (10), 4228-4237.

(6) Diment, W. T.; Gregory, G. L.; Kerr, R. W. F.; Phanopoulos, A.; Buchard, A.; Williams, C. K. Catalytic Synergy Using Al(III) and Group 1 Metals to Accelerate Epoxide and Anhydride Ring-Opening Copolymerizations. *ACS Catal.* **2021**, *11* (20), 12532-12542.

(7) Asaba, H.; Iwasaki, T.; Hatazawa, M.; Deng, J.; Nagae, H.; Mashima, K.; Nozaki, K. Alternating Copolymerization of CO_2 and Cyclohexene Oxide Catalyzed by Cobalt–Lanthanide Mixed Multinuclear Complexes. *Inorg. Chem.* **2020**, *59* (12), 7928-7933.

(8) Deacy, A. C.; Kilpatrick, A. F. R.; Regoutz, A.; Williams, C. K. Understanding metal synergy in heterodinuclear catalysts for the copolymerization of CO_2 and epoxides. *Nat. Chem.* **2020**, *12* (4), 372-380.

(9) Li, J.; Liu, Y.; Ren, W.-M.; Lu, X.-B. Asymmetric Alternating Copolymerization of Meso-epoxides and Cyclic Anhydrides: Efficient Access to Enantiopure Polyesters. *J. Am. Chem. Soc.* **2016**, *138* (36), 11493-11496.

(10) Cui, L.; Ren, B.-H.; Lu, X.-B. Trinuclear salphen–chromium(III)chloride complexes as catalysts for the alternating copolymerization of epoxides and cyclic anhydrides. *J. Polym. Sci.* **2021**, *59* (16), 1821-1828.

(11) Thevenon, A.; Garden, J. A.; White, A. J. P.; Williams, C. K. Dinuclear Zinc Salen Catalysts for the Ring Opening Copolymerization of Epoxides and Carbon Dioxide or Anhydrides. *Inorg. Chem.* **2015**, *54* (24), 11906-11915.

(12) Diment, W. T.; Rosetto, G.; Ezaz-Nikpay, N.; Kerr, R. W. F.; Williams, C. K. A highly active, thermally robust iron(iii)/potassium(i) heterodinuclear catalyst for bio-derived epoxide/anhydride ring-opening copolymerizations. *Green Chem.* **2023**, *25* (6), 2262-2267.

(13) Plajer, A. J.; Williams, C. K. Heterotrinuclear Ring Opening Copolymerization Catalysis: Structure–activity Relationships. *ACS Catal.* **2021**, *11* (24), 14819-14828.

(14) Cyriac, A.; Lee, S. H.; Varghese, J. K.; Park, E. S.; Park, J. H.; Lee, B. Y. Immortal CO2/Propylene Oxide Copolymerization: Precise Control of Molecular Weight and Architecture of Various Block Copolymers. *Macromolecules* **2010**, *43* (18), 7398-7401.

(15) Nakano, K.; Kamada, T.; Nozaki, K. Selective Formation of Polycarbonate over Cyclic Carbonate: Copolymerization of Epoxides with Carbon Dioxide Catalyzed by a Cobalt(III) Complex with a Piperidinium End-Capping Arm. *Angew. Chem. Int. Ed.* **2006**, *45* (43), 7274-7277.

(16) Cohen, C. T.; Chu, T.; Coates, G. W. Cobalt Catalysts for the Alternating Copolymerization of Propylene Oxide and Carbon Dioxide: Combining High Activity and Selectivity. *J. Am. Chem. Soc.* **2005**, *127* (31), 10869-10878.

(17) S, S.; Min, J. K.; Seong, J. E.; Na, S. J.; Lee, B. Y. A Highly Active and Recyclable Catalytic System for CO_2/Propylene Oxide Copolymerization. *Angew. Chem. Int. Ed.* **2008**, *47* (38), 7306-7309.

(18) Hu, L.-F.; Zhang, C.-J.; Wu, H.-L.; Yang, J.-L.; Liu, B.; Duan, H.-Y.; Zhang, X.-H. Highly Active Organic Lewis Pairs for the Copolymerization of Epoxides with Cyclic Anhydrides: Metal-Free Access to Well-Defined Aliphatic Polyesters. *Macromolecules* **2018**, *51* (8), 3126-3134.

(19) Abel, B. A.; Lidston, C. A. L.; Coates, G. W. Mechanism-Inspired Design of Bifunctional Catalysts for the Alternating Ring-Opening Copolymerization of Epoxides and Cyclic Anhydrides. *J. Am. Chem. Soc.* **2019**, *141* (32), 12760-12769.
